# CSmetaPred: a consensus method for prediction of catalytic residues

**DOI:** 10.1186/s12859-017-1987-z

**Published:** 2017-12-22

**Authors:** Preeti Choudhary, Shailesh Kumar, Anand Kumar Bachhawat, Shashi Bhushan Pandit

**Affiliations:** 1 Department of Biological Sciences, Indian Institute of Science Education and Research, Mohali, Knowledge City, Sector 81, SAS Nagar, Manuali PO 140306 India; 2 Laboratory of Biochemistry and Genetics, National Institute of Diabetes and Digestive and Kidney Diseases, National Institutes of Health, Bethesda, MD 20892 USA

**Keywords:** Catalytic residue prediction, Meta-approach, Active site residues

## Abstract

**Background:**

Knowledge of catalytic residues can play an essential role in elucidating mechanistic details of an enzyme. However, experimental identification of catalytic residues is a tedious and time-consuming task, which can be expedited by computational predictions. Despite significant development in active-site prediction methods, one of the remaining issues is ranked positions of putative catalytic residues among all ranked residues. In order to improve ranking of catalytic residues and their prediction accuracy, we have developed a meta-approach based method CSmetaPred. In this approach, residues are ranked based on the mean of normalized residue scores derived from four well-known catalytic residue predictors. The mean residue score of CSmetaPred is combined with predicted pocket information to improve prediction performance in meta-predictor, CSmetaPred_poc.

**Results:**

Both meta-predictors are evaluated on two comprehensive benchmark datasets and three legacy datasets using Receiver Operating Characteristic (ROC) and Precision Recall (PR) curves. The visual and quantitative analysis of ROC and PR curves shows that meta-predictors outperform their constituent methods and CSmetaPred_poc is the best of evaluated methods. For instance, on CSAMAC dataset CSmetaPred_poc (CSmetaPred) achieves highest Mean Average Specificity (MAS), a scalar measure for ROC curve, of 0.97 (0.96). Importantly, median predicted rank of catalytic residues is the lowest (best) for CSmetaPred_poc. Considering residues ranked ≤20 classified as true positive in binary classification, CSmetaPred_poc achieves prediction accuracy of 0.94 on CSAMAC dataset. Moreover, on the same dataset CSmetaPred_poc predicts all catalytic residues within top 20 ranks for ~73% of enzymes. Furthermore, benchmarking of prediction on comparative modelled structures showed that models result in better prediction than only sequence based predictions. These analyses suggest that CSmetaPred_poc is able to rank putative catalytic residues at lower (better) ranked positions, which can facilitate and expedite their experimental characterization.

**Conclusions:**

The benchmarking studies showed that employing meta-approach in combining residue-level scores derived from well-known catalytic residue predictors can improve prediction accuracy as well as provide improved ranked positions of known catalytic residues. Hence, such predictions can assist experimentalist to prioritize residues for mutational studies in their efforts to characterize catalytic residues. Both meta-predictors are available as webserver at: http://14.139.227.206/csmetapred/.

**Electronic supplementary material:**

The online version of this article (10.1186/s12859-017-1987-z) contains supplementary material, which is available to authorized users.

## Background

In the post genomic era, one of the challenges is accurate protein function annotation as these could provide clues to insights into molecular details of biological processes [1, 2]. The task of protein function annotation combines cumbersome experimental studies with automated computational prediction methods, which usually allow molecular function annotation based on: establishing sequence/structure relationship between proteins of unknown function to proteins of known function, predicting putative binding sites for metals/chemical compounds/DNA/RNA/protein and prediction of catalytic residues of enzymes [2]. The knowledge of catalytic residues can also assist in elucidation of reaction mechanism apart from providing enhanced function annotation of enzymes.

In the past decade, many sequence and/or structure-based catalytic residue prediction methods have been developed that rely on remote homology recognition, statistical and machine-learning algorithms. The sequence based prediction methods used sequence homology information or/and conserved family patterns/motifs [3–5], sensitive sequence-based scoring functions, amino acid stereochemical features [6, 7], conservation scores such as Von Neumann entropy, relative entropy, Jensen-Shannon divergence and sum-of-pairs measure [3, 8] to predict catalytic residues. Other prediction methods used phylogenetic motifs and phylogenetic trees [9, 10]. CRpred is one of the best sequence based methods that uses various sequence features such as residue type, hydrophobicity, and PSI-BLAST profiles [11] in a Support Vector Machine (SVM) based binary classification of residues into catalytic and non-catalytic residues. With the availability of tertiary structures, many methods were developed that used structure similarity searches with pre-calculated active site structural motif/template library [12–14], such as CATSID [13]. Many other structure-based methods used structural features such as hydrophobicity distribution in protein [15], electrostatics [16], chemical properties [17], network centrality measures [18, 19], distribution of catalytic residues with centroid of structure [20], unusual central atomic distances [21], geometry based [22], contact density [23], structural neighbourhood [24] and side-chain orientation of catalytic residues [25, 26]. Many of these methods combine sequence and structural features to improve prediction accuracy [27–33]. For example, EXIA2 employs side-chain orientation of polar/charged residues and sequence features [25]; and DISCERN uses statistical models based on phylogenomic conservation score of sequence and several structural features [31] to predict catalytic residues.

Despite significant development in catalytic residue prediction methods, the ranked positions of known catalytic residues are on an average high among the list of all ranked residues. Improving the ranked positions of putative catalytic residues will facilitate and expedite their experimental identification and characterization. Moreover, an improved ranking of catalytic residues will also increase their prediction accuracy. To address these issues, we have developed methods based on meta-approach to predict active site residues that combine results from four well-known predictors to generate a consensus ranked list of residues. In the meta-predictor CSmetaPred, the residues are ranked based on the mean of normalized residue scores (meta-score) obtained from four predictors. Next, we included the predicted pocket information with the mean residue score or meta-score to further improve prediction performance in another meta-predictor, CSmetaPred_poc. Previously, meta-approaches have been shown to improve accuracy for protein structure and binding site predictions [34–36].

## Methods

### Benchmark datasets

We used five benchmark datasets to evaluate meta-predictors that include three datasets from previous studies, which we primarily used as a legacy dataset to compare predictions from previous prediction methods. In the present work, we compiled 2 datasets macie-254 and csalit-688. Macie-254 is derived from the MACiE (mechanism, annotation and classification in enzymes) database [37], which provides manually curated list of catalytic residues with their putative roles in mechanistic steps of an enzymatic reaction. From 335 MACiE entries, enzymes having catalytic site defined in single pdb chain were used to prepare a non-redundant set of 254 proteins at 60% sequence identity using CD-HIT [38]. Similarly, a non-redundant csalit-688 dataset (60% sequence identity) was generated from only literature annotated catalytic residues of pdb entries in Catalytic Site Atlas (CSA) database [39] and those not present in MACiE dataset. CSA database may annotate more than one catalytic site for a given single pdb chain depending on its reference source. Here, we merged two or more catalytic sites in a single pdb chain that have at least one common residue between them. The two datasets macie-254 and csalit-688 are combined to form a non-redundant CSAMAC dataset at 60% sequence identity using CD-HIT. Additionally, an unbound non-redundant (60% sequence identity) dataset, UB-137, was prepared from CSAMAC pdb entries, which are not bound to any ligand (pdb entries without HETATM record). The Table S1 in Additional file 1 provides list of datasets with pdb entries and their known catalytic residues. From earlier works, we took EF-Fold, POOL-160, and PW-79 datasets along with their respective catalytic residues definition [17, 30, 33] and pruned them to construct POOL-148, EF-Fold-164 and PW-79 (for details, see Additional file 2: S1 Text). Three datasets are pooled to construct a non-redundant (at 60% sequence identity) EF_POOL_PW dataset. Since pdb entries of EF_POOL_PW datasets are redundant with CSAMAC, we have described evaluation on CSAMAC dataset in the main text, whereas results from legacy datasets are provided in the supplementary material (Additional file 2). The average (standard deviation) number of catalytic residues in CSAMAC and EF_POOL_PW datasets is 3.3 (1.9) and 3.2 (1.9) respectively.

### Overview of method

We have chosen four well-known active site prediction methods viz. CRpred, CATSID, DISCERN and EXIA2 for implementing in meta-predictors. These methods are selected primarily based on their prediction performances and their availability either as source code or easily automatable webservers. Moreover, these also are representative of different input features (sequence or/and structure) used for catalytic residue prediction. Among these, CRpred rely on only sequence derived features, CATSID uses only structural features, whereas, DISCERN and EXIA2 employ both sequence and structural properties for prediction of catalytic residues.

**Fig. 1:**
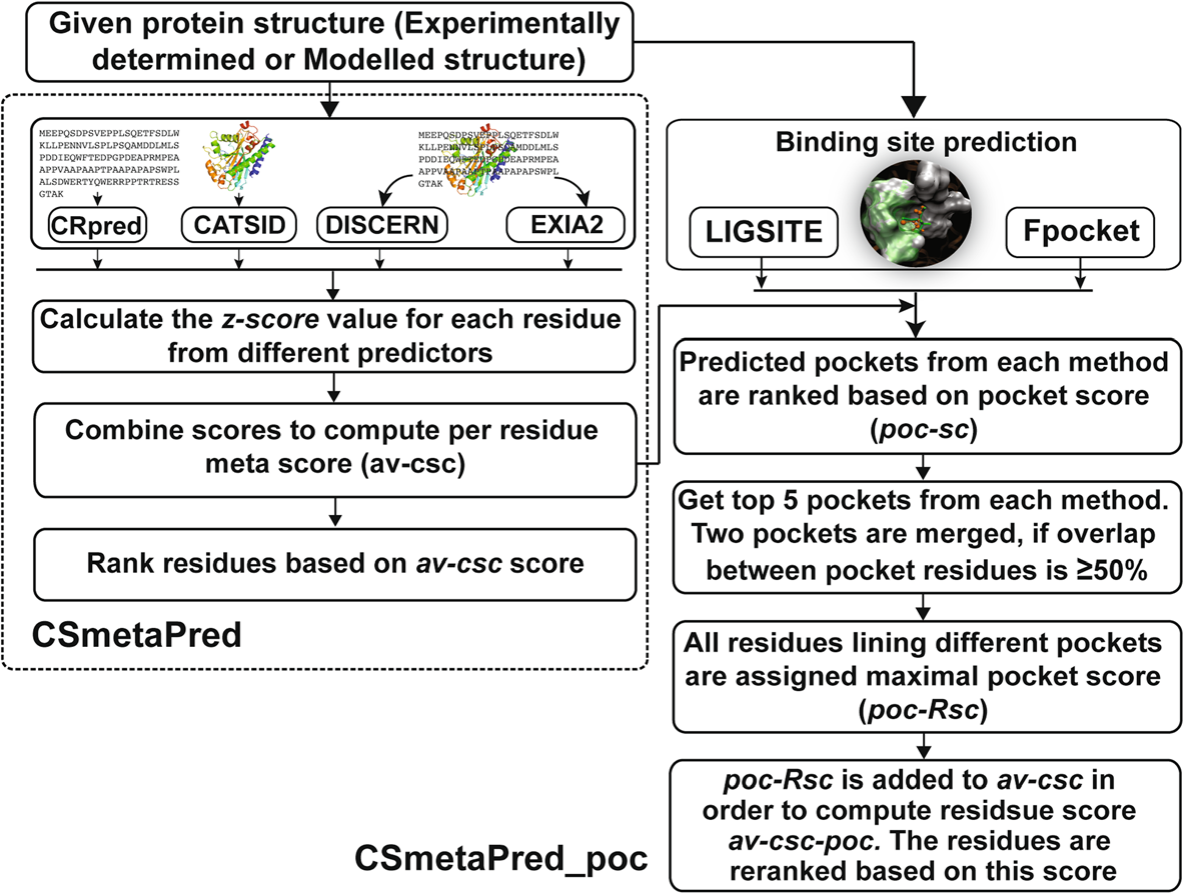
Overview of methodology. Flowchart showing important steps in CSmetaPred and CSmetaPred_poc methods

In order to combine varied prediction output types such as binary prediction from CRpred, structurally similar active-site templates from CATSID and residues scores from EXIA2/DISCERN, first, we obtain or assign a score possibly for every residue from each method and then normalize residue scores to calculate mean normalized residue score or meta-score. This meta-score is used for ranking of residues in CSmetaPred. The rationale behind this is that a residue having high score consistently from several predictors is most likely to be the catalytic residue. In CSmetaPred_poc, we combine meta-score with predicted pocket information to predict catalytic residues. Overview of both methods is shown in Fig. 1.

The webservers of CATSID (http://catsid.llnl.gov/) and EXIA2 (http://203.64.84.196/) were used for catalytic residues predictions. We have used EXIA2 webserver for prediction of proteins in benchmark studies. However, due to temporary unavailability of EXIA2 webserver, we have recoded EXIA2 and implemented in CSmetaPred webserver. We locally executed CRpred and DISCERN suites of program to predict catalytic residues using the packages obtained from developers’ websites (http://biomine.ece.ualberta.ca/CRpred/CRpred.htm) and (http://phylogenomics.berkeley.edu/software/) respectively. The procedure to derive residue score for various predictors is described below.

The score assigned to each residue from DISCERN and CRpred outputs are taken for meta-score calculation. These DISCERN and CRpred scores are referred to as S_di_ and S_cr_ respectively. From EXIA2 webserver parsed outputs, we took rank score and WCN assigned to residues as two independent scores for computing meta-score. The residue rank score, combines score for average side chain vector directions of its neighboring residues, amino acid combinations, structural flexibility and sequence conservation [25]. WCN score is a measure of structural flexibility that is either obtained from EXIA2 output or is calculated using previously described algorithm [40]. The rank score (S_rs_) is defined only for 12 amino acids (R, N, D, C, Q, E, H, K, S, T, Y and W), whereas WCN score (S_wcn_) is derived for all residues. Unlike other predictors, the CATSID outputs a list of hit templates and their associated template score, which is a measure of likelihood that a query protein shares catalytic function with the template. CATSID also provides alignment between the query and catalytic residues of template. To obtain residue score (S_ca_), we assign a template score to the aligned query residues in the alignment between query and template. If a residue is present in more than one alignment, we sum the score from each query template alignment and assign this summed score to the residue. Here, we have used all templates irrespective of any previously suggested score cut-off.

To compute meta-score for each protein residue, first we normalize residue score obtained from each method with their respective mean and standard deviation. The normalized residue score *zSc(ij)* is defined as:

$$ zSc(ij)=\left(S(ij)-\mu (j)\right)/\sigma (j) $$

where, *zSc(ij)* and *S(ij)* are normalized and raw scores of residue *i* for method *j* respectively; μ(j) and σ(j) are mean and standard deviation for method *j* scores respectively. Then, we calculate mean of normalized residue scores for each residue referred to as meta-score or *av-csc* score, which is defined as:

$$ av-\csc (i)=\frac{\sum \limits_{j=1}^5 zSc(ij)\ast p(j)}{\sum \limits_{j=1}^5p(j)} $$

where, *zSc(ij)* is z-score of residue *i* for method *j* and *p(j)* is binary function with *p(j)* = 1 for residue having a assigned score, or 0 otherwise. The *av-csc* score is used in CSmetaPred to rank residues for every protein, wherein high score represents a greater chance for it to be a catalytic residue.

Exploiting the fact that most catalytic residues are either part of substrate binding sites or spatially proximal to these sites [24], we have developed another version of CSmetaPred referred as CSmetaPred_poc. In this approach, we combine residue meta-score with pockets/clefts predicted from Fpocket [41] and LIGSITE [42]. For this, first we select predicted pockets from Fpocket and LIGSITE and then merge these pockets to generate a combined list of pockets. To select pockets for merging, we rank pockets based on pocket score (*poc_sc*). For each pocket *i, poc_sc(i)* is defined as:

$$ poc\_ sc(i)=\left({\sum}_{j=1}^{Nres(i)} av-\mathit{\csc}(j)\right)/ Nres(i) $$

where, *av-csc(j)* is meta-score of pocket residue *j*, *Nres(i)* is number of residues in a given pocket *i.*

We selected top 5 ranked pockets from both methods and merge two pockets having ≥50% number of common residues between them. Thus, we generate a combined list of predicted pockets from both LIGSITE and Fpocket. The parameters for pocket ranking and merging were optimized using macie-254 dataset (Additional file 2: Figure S1).

Next, each residue lining the pocket is assigned a pocket residue score (*poc_Rsc*), which is essentially pocket score (*poc_sc*) of the pocket. If a residue is present in more than one pocket, the maximum of *poc_Rsc* from all pockets is computed and assigned to that residue. A *poc_Rsc* score of 0 is assigned to residues, which are not part of any pocket. The *poc_Rsc* is linearly combined with *av-csc* to calculate residue *av-csc-poc* score, defined as:

$$ av-\mathit{\csc}- poc(i)= av-\mathit{\csc}(i)+ poc\_ Rsc(i) $$

In CSmetaPred_poc, residues are ranked based on *av-csc-poc* score.

### Generation of homology models

To improve catalytic residues prediction of enzymes, without known tertiary structure, we have evaluated meta-predictor performance on homology modelled protein structures built using MODELLER [43]. The protein models are built based on a single template structure with sequence identities ranging from 40% to 90% between query and template sequences. Details of dataset and construction of template library are given in supporting information (Additional file 2: S1 Text).

Each full-length protein sequence from CSAMAC dataset is queried against template library (LIB_TEMP) using *profile_build()* module of MODELLER to select a set of 335 proteins, which have sequence identity from 40 to 90% and coverage ≥70% to template sequences. The templates for 335 protein sequence is identified by searching these sequence against template library (LIB_TEMP) using *profile_build()* module of MODELLER. The templates with sequence identity <40% and >90% or query coverage <70% are discarded from the list of possible templates. Next, each query and template alignment having sequence identity ranging 40%–90% is grouped into sequence identities bins of 40–50%, 50–60%, 60–70%, 70–80% and 80–90% (see Additional file 2: S1 Text) with each bin having 235, 135, 53, 22 and 23 query-template alignments respectively. For each query-template alignment, we generated 10 models using *align2d()* module and the best model (having the lowest DOPE energy score) was used for prediction. Thus, we generated a total of 468 models for 335 query protein sequences.

### Evaluation of method

The predictors are primarily evaluated using Receiver Operating Characteristic (ROC) and Precision Recall (PR) curves, which are frequently used in assessment of binary classifiers. As most of methods used in this study provide ranked list of residues, we create a binary classification by selecting top *n* ranked list as predicted catalytic residues and rest as non-catalytic residues. Hence, true positives (TP) are correctly predicted catalytic residues; false negatives (FN) are catalytic residues predicted as non-catalytic; false positives (FP) are non-catalytic residues predicted as catalytic; true negatives (TN) are correctly predicted non-catalytic residues. The precision, True Positive Rate (TPR) and False Positive Rate (FPR) are defined as:

Precision = TP / (TP + FP)

TPR (recall) = TP / (TP + FN)

FPR (1-specificity) = FP/ (FP + TN)

We have used vertical average ROC curves to represent and compare prediction performance that is generated by averaging recall values at all FPR values (0–1) [44] for all proteins in the dataset. TPR is linearly interpolated, in case it is not computed at a given FPR. As a scalar measure to assess performance and compare ROC curves, we calculate Area Under Curve of ROC curve (AUCROC) and Mean Average Specificity (MAS) [17], which is mean of Average Specificity (AveS):

$$ AveS=\frac{\sum_{r=1}^NS(r)\ast pos(r)}{Npos} $$

where, *r* is rank, *N* is number of residues in a protein, *pos(r)* is binary function with *pos(r)* = 1 for known catalytic residue or 0 otherwise and *S(r)* is the specificity at a given cutoff rank *r*, *Npos* is the total number of positive examples (catalytic residues in this case).

It has been shown that PR curves can show differences among classifiers not apparent in ROC curves in datasets having a skew in the total number of positives with negative counts [45, 46]. As the number of catalytic residues (positives) is far less than non-catalytic residues, we have employed PR curves to evaluate performance in the present analysis. The average PR curve for all proteins in a dataset is generated by averaging precision for every recall value. If a protein does not have recall value, it is interpolated using local skew [45]. We have used AUCCalculator to generate data for PR curves [45]. To compare average PR curves using a single measure, we calculate Area Under PR Curve (AUCPR) and Mean Average Precision (MAP) [47], which is frequently used in information retrieval. MAP is mean of average precision (AP), which is defined as the arithmetic mean of precisions for a set of top *n* residues after each true positive (catalytic residue) is retrieved. This measure of quality across recall levels has been suggested to have good discrimination and stability [47]. MAP is defined as:

$$ MAP=\frac{1}{N}{\sum}_{i=1}^N\frac{1}{n_i}{\sum}_{j=1}^{n_i} Precision\left({R}_{ij}\right) $$

where, *N* is total number of proteins in the dataset; for protein *i, n*_*i*_ is the number of true positives and Precision (*R*_*ij*_) is precision calculated at the rank *R*_*ij*_ at which true positive *j* for protein *i* is retrieved in the ranked list.

The measures mentioned above are used to compare prediction performances of CSmetaPred, CSmetaPred_poc, CRpred, EXIA2, DISCERN and WCN. The procedure adopted to rank residues from CRpred, EXIA2, DISCERN and WCN is given in supporting information (Additional file 2: S1 Text).

## Results and discussions

As mentioned before, we have evaluated meta-predictors and compared them with their constituent methods on five benchmark datasets using average ROC and PR curves. In the present work, we have not included CATSID for comparison because using our approach of assigning score to residues from the template match score obtained from CATSID could provide score/rank only for subset of residues, whereas other methods rank all residues.

### CSmetaPred prediction performance

**Fig. 2:**
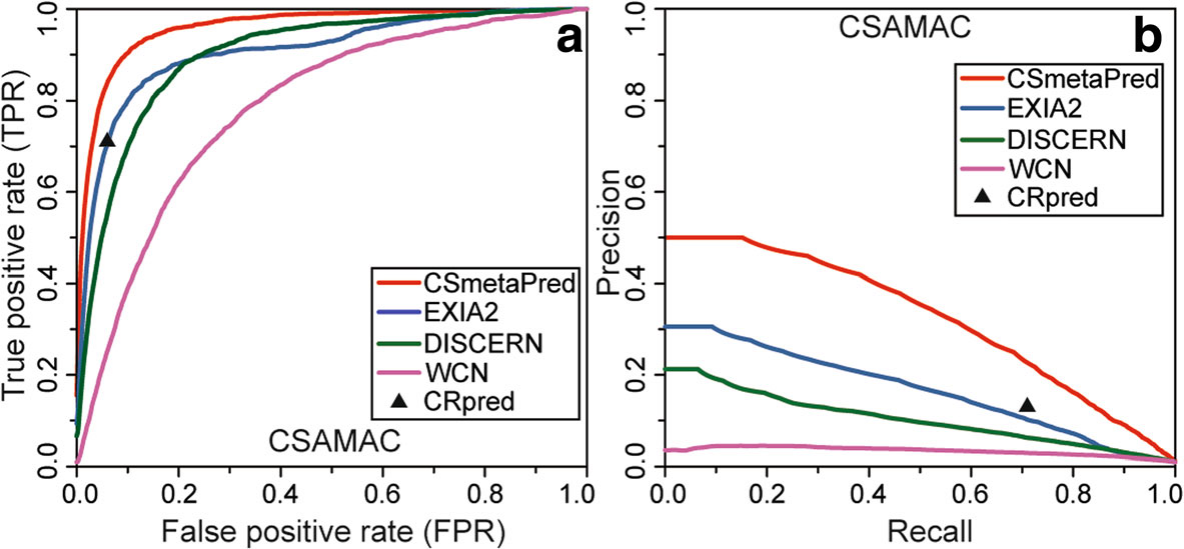
Average ROC and PR curves for CSAMAC dataset. Figure showing comparison of CSmetaPred with its constituent methods for CSAMAC dataset using (**a**) Average ROC and (**b**) Average PR curves. CRpred SVM performance is shown as filled triangle

First, we have used average ROC curves to evaluate and compare prediction performance of CSmetaPred with other methods used in meta-score calculation. A visual comparison of average ROC curves for CSmetaPred and its constituent methods (DISCERN, EXIA2 and WCN; CRpred SVM performance) on CSAMAC dataset (Fig. 2a) clearly shows that CSmetaPred outperforms other methods. Importantly, at any given FPR, CSmetaPred has higher recall in comparison to other methods. Furthermore, comparison of ROC curves using MAS (Table 1) and AUCROC (Additional file 2: Table S2) shows that CSmetaPred is the best among evaluated methods. For instance, on CSAMAC dataset MAS values for CSmetaPred, EXIA2, DISCERN, and WCN score are 0.961, 0.910, 0.901, and 0.786 respectively (Table 1). Since MAS enables comparison of average performance among methods, it is important to find whether improvement of CSmetaPred over its constituent methods is statistically significant. So, we estimated the statistical significance of performance difference between CSmetaPred and other methods by considering pairwise comparison of *AveS* (see Methods section), calculated for each pdb entry in a given dataset, from two methods. Using Wilcoxon signed-rank test, the performance difference between CSmetaPred and other methods is found to be statistically significant (*p*-value <0.0001; see Additional file 2: Table S3). Moreover, this statistical test on *AveS* also indicates consistent performance of a method on per protein basis. We observed similar CSmetaPred performance on EF_POOL_PW and individual datasets (Additional file 2: Figure S2; Tables S2 and S3). To decipher contribution of each method in improving prediction of CSmetaPred, we re-calculated meta-score by taking only four scores at a time. As evaluated by average ROC curves (Additional file 2: Figure S3), all methods seem to contribute in improving CSmetaPred prediction.

**Table 1:** Summary of MAS, MAP and catalytic residues median rank

Method	MAS	MAP	Median rank
CSAMAC dataset (884 protein)			
CSmetaPred_poc	0.968	0.514	6.0
CSmetaPred	0.961	0.489	7.0
EXIA2	0.910	0.317	14.5
CRpred	–	–	14.0
DISCERN	0.901	0.226	23.0
WCN	0.786	0.081	53.4

Next, we have used PR curve to compare CSmetaPred performance with a motive to evaluate how well it classifies positives unlike ROC, which also consider misclassification of negatives. As is evident from the visual inspection of average PR curves (Fig. 2b) that CSmetaPred has better performance than EXIA2, WCN, DISCERN and CRpred SVM performance. This is also apparent from MAP (Table 1) and AUCPR (Additional file 2: Table S2) measures, which are used as a scalar value for comparison of PR curves. On CSAMAC dataset, MAP for CSmetaPred is highest (0.489) among its constituent methods (Table 1). Similarly, AUCPR of CSmetaPred is highest with a value of 0.324 followed by EXIA2, DISCERN and WCN having values of 0.167, 0.103 and 0.034 respectively (Additional file 2: Table S2). Importantly, evaluation of differences in prediction performances are found to be statistically significant (Wilcoxon signed-rank test with *p*-value <0.0001, see Additional file 2: Table S3) when we compared AP (described in Methods) calculated for each protein between CSmetaPred and other methods in a pairwise comparison (per protein basis). Both visual and quantitative analysis of PR curves show that CSmetaPred prediction is better than its constituent methods on EF_POOL_PW and five individual datasets (Additional file 2: Figure S4 for PR curves; MAP and AUCPR are summarized in Table S2). As CSmetaPred has comparatively lower median and average rank for catalytic residues (Table 1), this also suggests that CSmetaPred is able to improve catalytic residues ranks in comparison to other methods.

Further, we compared CSmetaPred predicted ranks of catalytic residues to their best possible ranks derived from CRpred, DISCERN and EXIA2. Here, the best possible rank of a residue is the minimum rank assigned by scores from above mentioned methods. It is important to note that in this analysis we have excluded CATSID because we could rank only subset of residues for which template was identified by CATSID (see Methods section). This inability to rank all residues will lead to lower ranks and which may not imply a necessarily better performance. The ‘best possible rank’ is a theoretical best scenario for selecting ranks for catalytic residue and this provides an upper bound of meta-approach performance. The comparison of ranks is performed on CSAMAC dataset having 2912 catalytic residues. Since lower ranked catalytic residues will largely affect CSmetaPred performance, we analyzed catalytic residues having the best possible rank less than 20. Most (86.9%) of catalytic residues have the best possible rank ≤20. Of these, for ~51% of catalytic residues CSmetaPred predicted ranks are either unchanged or improved marginally having median and mean decrease in rank of 2 and 3.4 respectively. Further, CSmetaPred predicted ranks are higher (poorer) for ~49% of catalytic residues compared to the best possible rank. Importantly, the increase in CSmetaPred predicted rank is not large as evident from median and mean rank increases of 3 and 7.6 respectively. The detailed analysis of catalytic residues with increase in CSmetaPred ranks showed that in most instances these residues are predicted only by one or two methods, which is exhibited in their higher normalized scores, whereas other methods assign lower residue scores as predictions from other methods are not good. This indicates that even though meta-predictor is not able to achieve the best possible scenario in meta-approach, it does not decrease ranks of catalytic residues drastically from the best possible scenario.

**Fig. 3:**
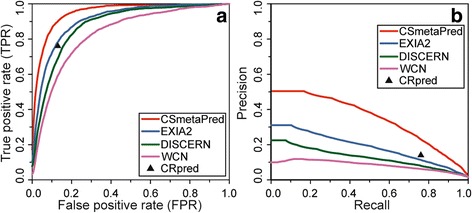
Comparison of prediction performances on polar/charged residues. Figure showing average ROC (**a**) and average PR curves (**b**) on CSAMAC dataset, when only polar or charged residues are ranked. CRpred SVM performance is shown as filled triangle

Since catalytic residues are mostly polar or charged amino acids (>90%) [48], we have evaluated and compared CSmetaPred to its constituent methods when polar/charged and non-polar residues are ranked separately. Here, we consider any amino acid having functional side chain with a role in catalytic activity as polar/charged set, which consists of 12 amino acids as defined previously in EXIA [25] (see Methods). Rest 8 amino acids (P, F, A, V, I, L, M and G) are considered as non-polar set. Average ROC and PR curves for polar/charged amino acids on CSAMAC dataset are shown in Fig. 3a and b respectively. It is apparent from the curves that CSmetaPred prediction performance is consistently better than other methods. Moreover, this is supported by quantitative comparison of average ROC curves using MAS and PR curves using MAP (Table 2). Importantly, similar performances are observed on other datasets (Additional file 2: Figure S5; Table A in Table S4). The evaluation of performance differences between CSmetaPred and its constituent methods by pairwise comparison of AveS/AP on per protein basis is found to be statistically significant (*p*-value <0.0001 using Wilcoxon signed-rank sum test). In case of non-polar amino acids, visual and quantitative analysis of ROC and PR curves show that CSmetaPred is the best performing method (Additional file 2: Figure S6 and Table B in Table S4). These analyses suggest CSmetaPred has ability to improve ranks for both polar/charged as well non-polar residues.

**Table 2:** Summary of MAS, MAP, and median ranks of known catalytic residues when only polar/charged residues are ranked

Method	MAS	MAP	Median rank
CSAMAC Polar dataset (873 protein)			
CSmetaPred_poc	0.961	0.545	5.0
CSmetaPred	0.953	0.519	5.5
EXIA2	0.911	0.343	10.5
CRpred	–	–	11.0
DISCERN	0.883	0.265	15.0
WCN	0.832	0.186	20.3

### Comparison of CSmetaPred_poc with CSmetaPred

**Fig. 4:**
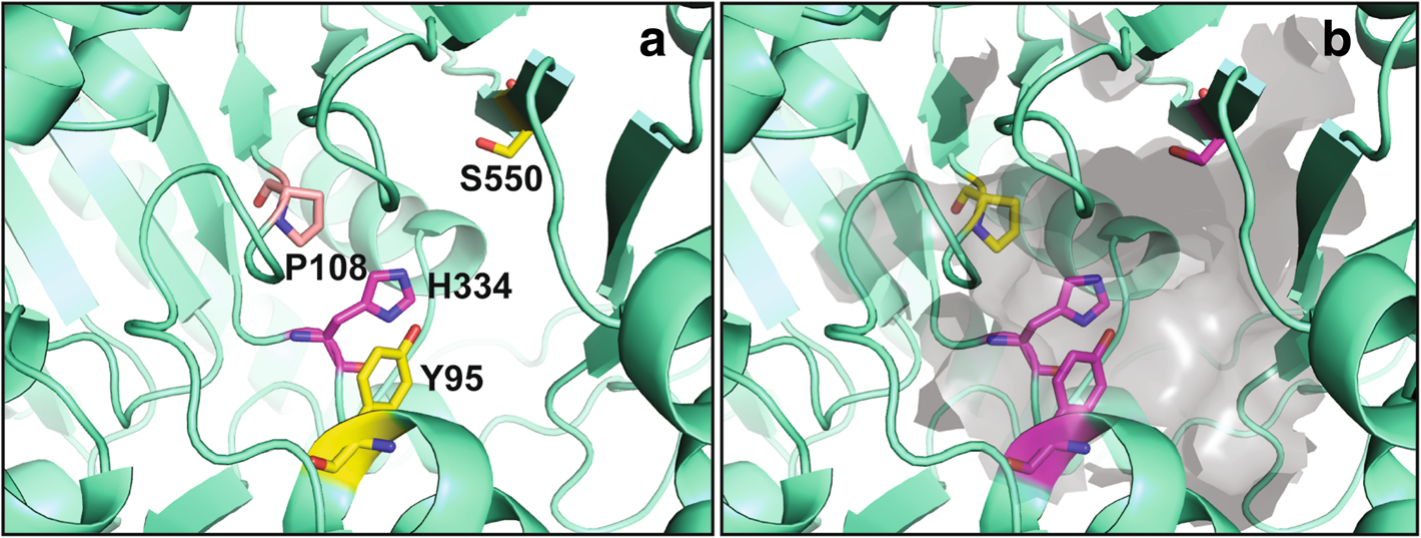
An example of catalytic residue predictions from CSmetaPred and CSmetaPred_poc. Comparison of prediction results for enzyme rat choline acetyltransferase (PDB: 1q6xB) from CSmetaPred (**a**) and CSmetaPred_poc (**b**) after including pocket information. Tertiary structure and known catalytic residues are shown in cartoon and licorice representations respectively. Catalytic residues are colored based on their meta-predictor predicted ranks: magenta for residues with rank ≤5, yellow for rank >5 and ≤10 and salmon color for rank >20. Top pocket ranked by pocket score is shown in gray transparent surface representation

As catalytic residues are known to be spatially proximal to substrate/cofactor binding sites, we evaluated whether including pocket information can increase the accuracy of catalytic residue prediction. For this, we have developed CSmetaPred_poc, which combines meta-score with a score (poc-Rsc) harboring information of combined predicted binding pockets from Fpocket and LIGSITE (see Methods). The visual inspection and quantitative analysis (MAS and MAP shown in Table 1) of both average ROC and PR curves (Additional file 2: Figure S7 and S8) show that CSmetaPred_poc has better prediction performance than CSmetaPred. For instance, on CSAMAC dataset MAP of CSmetaPred_poc and CSmetaPred is 0.514 and 0.489 respectively (Table 1). Moreover, performance of CSmetaPred_poc is found to be statistically significantly (*p*-values <0.0001 using paired Wilcoxon signed-rank test) better than CSmetaPred, when we considered the statistical significance of pairwise differences in AveS/AP calculated for each protein between these two meta-predictors. This suggests that CSmetaPred_poc is able to exploit predicted pocket information to improve catalytic residue prediction. For instance, CSmetaPred predicted catalytic residues viz. H334, Y95, S550, and P108 of rat choline acetyltransferase (pdb id: 1q6x chain B) at ranked positions of 1, 6, 8, and 27 respectively. These four residues are present in the top ranked predicted pocket (Fig. 4) of CSmetaPred_poc and adding the pocket residue score to residue meta-score leads to improved ranking of catalytic residues. Thus, CSmetaPred_poc results in improved ranking of H334, Y95, S550, and P108 at positions 1, 2, 3, and 11 respectively.

### Performance of CSmetaPred_poc on ligand unbound structures

Since CSmetaPred_poc prediction relies on predicted pockets information to improve its prediction, we assessed any bias in pocket prediction due to ligand/s (substrate/product/cofactor) bound to proteins in pdb structures. For this assessment, we performed prediction using non-redundant pdb entries without any ligand bound pdb dataset (UB-137). The detailed analysis of average ROC and PR curves show that CSmetaPred_poc is still the best performing method (Additional file 2: Figure S9). CSmetaPred_poc achieves MAS and MAP values on UB-137 dataset of 0.976 and 0.620 respectively (Additional file 2: Table S2).

### Catalytic residues rank analysis

Having shown that meta-predictors are the best among evaluated methods, next we have analyzed ranked position of known catalytic residues from all methods. As a metric to compare methods, we have used median rank of catalytic residues. Both meta-predictors achieve lower (better) median rank in comparison to other methods across all datasets (Table 1 and see Additional file 2: Table S2). In fact, CSmetaPred_poc achieves the lowest catalytic residue median rank of 6. The same is observed when either polar/charged or non-polar residues (Table 2 and Additional file 2: Table S4) are ranked separately.

**Fig. 5:**
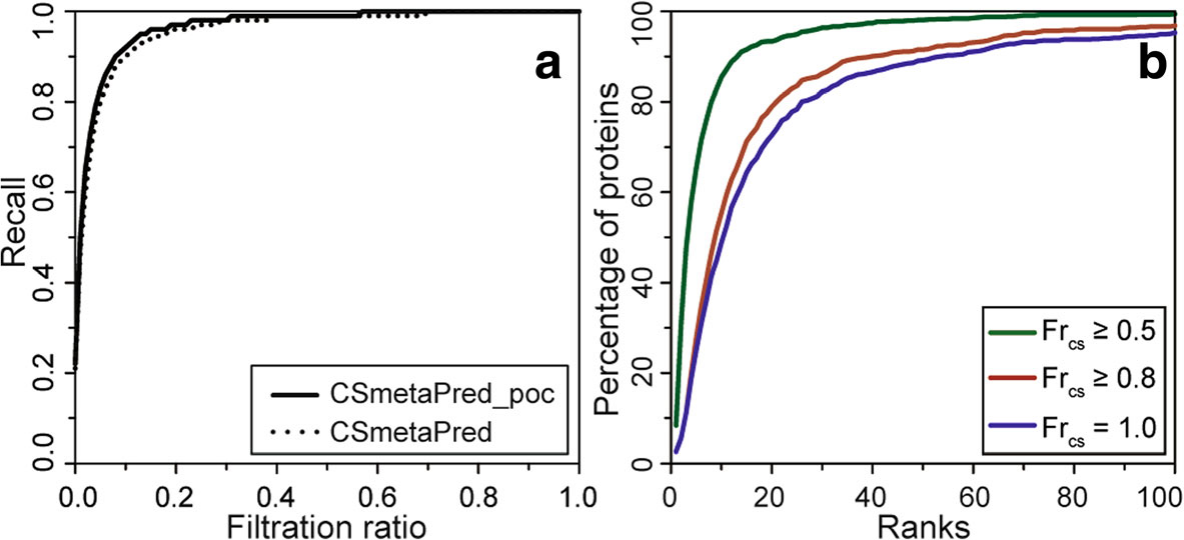
Catalytic residues rank analysis. Figure summarizing **a**) Average recall as a function of filtration ratio. **b**) Cumulative fraction of proteins (shown in percentage) having catalytic residue coverage of at least 0.5, 0.8 and 1.0 calculated at ranks ≤100

CSmetaPred/CSmetaPred_poc provides rank for all residues. However, there is no particular rank or score cut-off to select active site residues. To choose most likely catalytic residues, we have analyzed 2 criteria: a) select top *k* percent of residues from ranked list; and b) select top *p* ranked residues. In the first criterion, at various top *k* percent of residues predicted as positives, referred to as filtration ratio, we calculated mean recall and represent this graphically as Recall Filtration Ratio (RFR) curve. The average RFR curves show that CSmetaPred_poc, mostly, achieve higher recall than CSmetaPred at any given filtration ratio on CSAMAC dataset (Fig. 5a). For instance, taking 5% of residues from the ranked list give an average recall of 0.83 and 0.80 for CSmetaPred_poc and CSmetaPred respectively. The same is also observed for other dataset (Additional file 2: Figure S10). In the second criterion, we calculate percentage of proteins having at least 0.5, 0.8 and 1.0 catalytic residue coverage (fraction of known catalytic residues predicted by CSmetaPred_poc) at all ranks. The plot for fraction of proteins (shown in percentage) having specified catalytic residues coverage at various ranks (Fig. 5b) on CSAMAC dataset shows that there is rapid increase in number of enzymes at lower ranks that reach a plateau at higher ranks, typically around 30. Interestingly, at rank ~30 all catalytic residues are identified in ~82% of enzymes. Moreover, at lower ranks, such as within rank 20 CSmetaPred_poc correctly predicts ≥50% of catalytic residues for ~93% of proteins and all catalytic residues for ~73% of proteins. Importantly, this is consistently observed with other datasets (Additional file 2: Figure S11). These analyses suggest that meta-predictors are able to rank putative catalytic residues at lower (better) ranked positions, which is also observed in median ranks. Moreover, in most enzymes greater than half of their catalytic residues are within top 20 ranks in CSmetaPred_poc. This is important because it can help experimentalist to prioritize residues for mutational studies in their efforts to identify and characterize catalytic residues.

We are not committed to any specific cut-off to select active site residues. However, to prioritize residues for experimental studies we analyzed our results to find a rank or filtration ratio cut-off to select active site residues. Based on average precision, average recall, and average accuracy from all datasets, we suggest residues with ranks ≤20 or ranks ≤4% filtration ratio cut-off as catalytic residues. On CSAMAC dataset, with 4% filtration ratio cut-off CSmetaPred_poc achieves the average precision, recall, and accuracy of 0.2, 0.79, and 0.96 respectively. Using same dataset and method the average precision, recall and accuracy with rank 20 are 0.14, 0.87, and 0.94 respectively. Similar average values are observed in other datasets. These cut-off values are to be used as an indicator rather than a rule to predict catalytic residues. Using these criteria, CSmetaPred_poc is able to rank ~87% and ~76% of known catalytic residues within top 20 ranks and 4% filtration ratio respectively.

As mentioned before (see Methods section) the binary classified residues generated by varying ranks in CSmetaPred_poc ranked list of residues is used for comparison of CSmetaPred_poc with other classifiers. On PW-79 dataset, Cilia and Passerini method achieves average recall and precision of 0.46 and 0.28 respectively [24]. With the same dataset, at a recall of 0.46 CSmetaPred_poc has precision of 0.54 and at a precision 0.28 it has recall of 0.87. CRpred achieves average recall of 0.54 and precision of 0.175 on PW-79 dataset [11]. CSmetaPred_poc achieves a precision of 0.50 at same recall of 0.54 and a recall of 0.94 at same precision of 0.175. A similar comparison with other datasets (POOL-148 and EF-Fold-164) is difficult, as we have excluded some pdb entries from these datasets (see Methods).

### Catalytic residue prediction using protein models

Since experimental tertiary structures of many enzymes are not yet known, we evaluated whether modelled structures could be used for reliable prediction using CSmetaPred_poc. Moreover, this also provides comparison of prediction performance between modelled structures and prediction based only on sequence information. In this analysis, we build full-length homology models for sequences, with known tertiary structure, using single template having sequence identity ranging between 40 to 90% between query and template sequences (see Methods section).

First, we assessed model quality using Root Mean Square Deviation (RMSD) between the model and native structure. The average RMSD is 3.0 Å between models and native structures. The analysis of models with high RMSD showed that it is mostly due to either a long N/C terminal region or part of query sequences without any template aligned regions. Moreover, in some cases there was large conformational change between template and native structure that lead to large RMSD between model and native structure. Most high RMSD between model and native structure was from lowest sequence identity bin of 40–50%. Next, we used average ROC and PR curves to compare CSmetaPred_poc performance on models and native structures. Based on qualitative (visual inspection) and quantitative comparisons of ROC/PR curves, prediction based on models does not show comparable performance with their respective native structures (Additional file 2: Figure S12 and Table S5). However, considering median rank of catalytic residues, models result in slightly higher (poor) rank of 8.4 than native structures (rank 6.3). The detail analysis showed that most of poor performance is from models in 40–50% sequence identity category. In comparison to CRpred, which used only sequence information for prediction, models perform better as assessed by visual inspection of ROC/PR curves (Additional file 2: Figure S12) and comparison of median/average rank. CRpred results in median and average rank of catalytic residues of 11 and 23.2 respectively. This shows that CSmetaPred_poc will result in better prediction than using only sequence information as in CRpred. A study performed on usefulness of modelled structures has also suggested that low quality predicted structures could be used for catalytic residues prediction [49].

### Case studies

We compared residue level prediction results from CSmetaPred_poc with other methods on a list of pdb entries obtained from previous work or generated in the present work, which were mostly part of benchmark datasets. CSmetaPred_poc is able to get similar or better rank in most of the cases (Additional file 2: Table S6). Further, we analyzed prediction results for structures, with known catalytic residues, deposited in RCSB PDB database [50] subsequent to development of our method. For this analysis, we manually searched PDB database for recently determined tertiary structures of enzymes having curated list of catalytic residues. Interestingly, CSmetaPred_poc is able to predict most catalytic residues within top 20 ranks (Additional file 2: Table S7) as we observed in benchmark datasets. Below, we discuss some examples having the best results from CSmetaPred_poc.

The experimental site-directed mutagenesis in thioesterase enzyme YbdB from *Escherichia coli* has identified H89, E63, S67, H54, and Q48 as putative catalytic residues [51]. Using YbdB structure (pdb id: 4k4c), CSmetaPred_poc is able to predict residues H89, E63, S67, H54, and Q48 residues at ranks 1, 2, 4, 5, and 19 respectively. A recent study on β-keto-acid cleavage enzyme family KCE (DUF849) has identified H46, H48, E143, R226, D231 as crucial catalytic residues and S82, T106, and E230 as important residues [52]. Interestingly, CSmetaPred_poc ranks H46, H48, E143, R226, D231, S82, T106, and E230 residues (pdb id: 2y7f), at 2, 4, 6, 1, 5, 14, 17, and 3 ranked positions respectively.

The catalytic residues of *Escherichia coli* γ-glutamylcysteine synthetase (GshA) are not yet characterized. Using *Escherichia coli* GshA tertiary structure (pdb id: 1v4gA [53]) we predicted catalytic residues for this enzyme. From the subset of top 20 predicted residues (Additional file 2: Table S8), we mutated R330 (rank 1), R235 (rank 11), Y131, (rank 16) and R132 (rank 20) to investigate their role in catalysis using previously described in vivo and in vitro assays [54]. Preliminary studies show no enzymatic activity for R330A mutant and reduced activity for mutants of R235, R132, and Y131 (Additional file 2: Figure S13 and Table S9) suggesting these could play a role in catalysis. Interestingly, R330 structural equivalent in GshA homologue from *Saccharomyces cerevisiae* (Sc-γ-GCS) (pdb id: 3ig5) is R472, which has also been suggested to be a catalytic residue [55]. Further detailed study is required to investigate specific role of R330 in catalysis.

CSmetaPred and CSmetaPred_poc are provided as a webserver, which is accessible at http://14.139.227.206/csmetapred/. This server can take sequence or structure as an input. In CSmetaPred server we use our in-house recoded EXIA2. The comparison of residue ranks between original EXIA2 server result and our coded program is shown in Additional file 2: Figure S14.

## Conclusions

We have developed meta-approach based catalytic residue prediction methods viz. CSmetaPred and CSmetaPred_poc that combine residue scores from four well known catalytic residue prediction methods. Based on visual and quantitative analysis of ROC and PR curves on benchmark datasets (including 3 legacy datasets), CSmetaPred shows improved prediction over its constituent methods. This approach of combining residues is simple yet effective in ranking catalytic residues. CSmetaPred_poc further improves prediction performance by including predicted pockets from LIGSITE and Fpocket. The assessment based on both ROC and PR curves shows that CSmetaPred_poc is the best of evaluated approaches. Importantly, known catalytic residues are at lower ranked (better) positions in prediction by both meta-predictors. This is also evident from the lowest median predicted rank of catalytic residues from CSmetaPred_poc in all datasets. CSmetaPred_poc achieves prediction accuracy of 0.94 on CSAMAC dataset taking residues below rank 20 as true positives. Moreover, on the same dataset CSmetaPred_poc predicted all catalytic residues for ~73% of enzymes within top 20 ranks. The benchmarking of CSmetaPred_poc on comparative modelled structures showed that predicted tertiary structures could be used reliably for catalytic residue predictions in absence of experimentally determined structures. These analyses suggest that meta-predictors could assist experimentalists in their efforts to experimentally identify and characterize catalytic residues by prioritizing residues for mutational studies.

## Additional files

Additional file 1: Table S1. Datasets used in present work. List of pdb entries along with known catalytic residues from six datasets. (PDF 576 kb)

Additional file 2: Text S1. Extended Methods and Results sections. **Table S2.** Table summarizing quantitative measures for ROC and PR curves. Quantitative comparison of average ROC curves using AUCROC and MAS as single value measures of ROC and AUCPR and MAP are used to quantitatively compare average PR curves (see Methods section). Median and average ranks of catalytic residues are also summarized. **Table S3.** Summary of *p*-values obtained from Wilcoxon signed ranked statistical test. Summary of p-values from Wilcoxon signed-rank test computed on AveS (MAS) and AP (MAP) measures to estimate statistical significance of performance difference between CSmetaPred and its constituent methods (EXIA2, DISCERN, and WCN). **Table S4.** Quantitative comparison of average PR and ROC curves for various methods when either polar/charged or non-polar residues are ranked separately. Comparison of ROC/PR curves quantitative measures when only (A) polar/charged amino acids and (B) non-polar amino acids are ranked. Quantitative measure of ROC is AUCROC and MAS, whereas PR curves are compared using AUCPR and MAP. Median and average ranks of catalytic residues are also summarized. **Table S5.** Comparison of CSmetaPred_poc prediction performance on modelled and their respective native structures. Summary of quantitative analysis of ROC and PR curves using AUCROC/MAS and AUCPR/MAP respectively, for CSmetaPred_poc prediction on model and their respective native structures. Median and average ranks of catalytic residues are also summarized. **Table S6.** Comparison of catalytic residues rank obtained from various methods on set of pdb entries mostly from benchmark dataset. Summary of known catalytic residues ranks given by various predictors for pdb entries mostly from previous and present works. **Table S7.** Catalytic residue prediction for protein structures deposited in PDB after development of CSmetaPred. Meta-predictor prediction performance on pdb entries, with experimentally known catalytic residues, submitted in RCSB PDB database subsequent to development of meta-approach method. Catalytic residue ranks from CSmetaPred_poc is summarized in the table. **Table S8.** Predicted catalytic residues of γ-glutamylcysteine synthetase. List of top 20 predicted catalytic residues of γ-glutamylcysteine synthetase from *E. coli* (pdbid: 1v4gA) by CSmetaPred_poc. **Table S9.** Summary of relative enzymatic activity of GshA mutants. Table showing enzyme activity (in vitro) of GshA mutants calculated with respect to wild type activity of enzyme. **Table S10.** Primers used for cloning. List of primers sets used in site overlap extension PCR in cloning mutant GshA. **Figure S1.** Cumulative distribution of catalytic residues present in predicted pockets. Plot showing cumulative distribution of catalytic residues within a given pocket rank on macie-254 dataset for: a) Pockets output from LIGSITE/Fpocket, b) re-ranked pockets using poc_sc score and, c) merged top 5 re-ranked pockets. The vertical line shows that at pocket rank 5 both LIGSITE and Fpocket have achieved close to the maximum catalytic residues identified within predicted pockets. The drastic increase in catalytic residues fraction after re-ranking in LIGSITE could also be due to merging of pockets within LIGSITE. **Figure S2.** Average ROC plots for all datasets. Average ROC plots to show comparison among various predictors (CSmetaPred, EXIA2, DISCERN and WCN) on POOL-148, PW-79, EF-Fold-164, csalit-688, macie-254 and EF_POOL_PW datasets. CRpred SVM performance is shown as filled triangle. **Figure S3.** Average ROC plots for modified CSmetaPred, wherein one score is excluded from meta-score computation. Average ROC curves showing effect of individual method on the performance of CSmetaPred using CSAMAC (A) and EF_POOL_PW (B) datasets. All four methods contribute to different extent towards improving meta-score based ranking in CSmetaPred. It is apparent from ROC curves that excluding CRpred or CATSID residue score has maximum effect on prediction performance. This suggests that these 2 methods have major contribution in meta-score. **Figure S4.** Average PR curves for benchmarking datasets. The figure showing average PR curves for CSmetaPred and other predictors (EXIA2, DISCERN and WCN) on EF_POOL_PW and five individual datasets. CRpred SVM performance is shown as filled triangle. **Figure S5.** Average ROC and PR curves for predictors considering ranked list of only polar/charged residues. Average ROC (A-F) and average PR (G-L) curves on EF_POOL_PW and 5 datasets, when only polar/charged amino acids are ranked. CRpred SVM performance is shown as filled triangle. **Figure S6.** Average ROC and PR curves for predictors considering ranked list of only non-polar residues. Figure showing average ROC (A, B) and average PR (C, D) curves for various predictors (CSmetaPred, EXIA2, DISCERN and WCN) when only non-polar amino acids are ranked from CSAMAC and EF_POOL_PW datasets. CRpred SVM performance is shown as filled triangle. **Figure S7.** CSmetaPred_poc comparison with CSmetaPred using average ROC plots. Average ROC plots showing comparison of prediction performance between CSmetaPred and CSmetaPred_poc on all datasets. **Figure S8.** CSmetaPred_poc comparison with CSmetaPred using average PR curves. Average PR curves showing comparison of prediction performance between CSmetaPred_poc and CSmetaPred on all datasets. **Figure S9.** Average ROC and PR curves for various predictors on UB-137 datasets. Average ROC (A) and average PR (B) curves for meta-predictors and other predictors (EXIA2, DISCERN and WCN) on UB-137 dataset. CRpred SVM performance is shown as filled triangle. **Figure S10.** Filtration ratio plotted as a function of average recall. Average recall plotted as a function of filtration ratio for proteins in EF_POOL_PW dataset. **Figure S11.** Fraction of proteins with catalytic residue coverage plotted as a function of ranks. Cumulative fraction of proteins (in percent) having at least 0.5, 0.8 and 1.0 catalytic residue coverage at various ranks ≤100. **Figure S12.** Comparison of CSmetaPred_poc prediction performance on models and their respective native structures. Figure showing prediction performances of CSmetaPred_poc on models and their respective native structures assessed using average ROC (A) and PR (B) curves. CRpred SVM performance is shown with filled triangle. **Figure S13.** In vivo complementation assay of GshA wild type and mutant enzymes. Figure showing in vivo complementation assay of predicted catalytic residues mutants of GshA enzyme. *Saccharomyces cerevisiae* strain ABC1195 plasmids bearing WT gshA or the different cysteine binding residues gshA mutant gene cloned under TEF promoter. The transformants were grown overnight in SD + GSH medium and used to re-inoculate secondary culture. Cells were harvested at OD_600_ = 0.6 and serially diluted (0.2 to 0.0002 OD_600_). 10 μl was spotted on SD medium with or without GSH as sole source of organic sulphur. The vector pTEF416 and EcGshA were used as negative and positive control respectively. **Figure S14.** Residues rank comparison between EXIA2 server and in-house recoded EXIA2. Plot showing residues rank comparison from EXIA2 server output and in-house recoded EXIA2 for (A) all residues, and (B) catalytic residues. The Pearson correlation coefficient between ranks for all residues obtained from EXIA2 and in-house program is 0.86, and the same for catalytic residues is 0.55. (PDF 5965 kb)
