# Supplementary material for: CSmetaPred: a consensus method for prediction of catalytic residues
Source: BMC Bioinformatics. 2017 Dec 22;18:583. doi: 10.1186/s12859-017-1987-z (PMC5741869; doi:10.1186/s12859-017-1987-z)
Supplement: Supplementary file 1 — Datasets used in present work. List of pdb entries along with known catalytic residues from six datasets. (PDF 576 kb) [file 12859_2017_1987_MOESM1_ESM.pdf]

Table S1: Datasets used in present work. List of pdb entries along with known catalytic residues for 6 datasets

POOL-148 dataset (148 proteins)

S.No.	PDB	Chain	Catalytic site residues
1	12as	A	ASP46, ARG100, GLN116
2	13pk	A	ARG39, LYS219, GLY376, GLY399
3	1a26	A	TYR907, GLU988
4	1a4i	A	LYS56
5	1a4s	A	ASN166, GLU263, CYS297
6	1ae7	A	GLY30, HIS48, ASP99
7	1afw	A	CYS125, HIS375, CYS403, GLY405
8	1ah7	A	ASP55
9	1akm	A	ARG106, HIS133, GLN136, ASP231, CYS273, ARG319
10	1alk	A	SER102, ARG166
11	1aop	A	ARG83, ARG153, LYS215, LYS217, CYS483
12	1apx	A	ARG38, HIS42, ASN71
13	1apy	B	THR183, THR201, THR234, GLY235
14	1aq2	A	HIS232, LYS254, ARG333
15	1aw8	B	TYR58
16	1b3r	A	ASP130, LYS185, ASP189, ASN190, CYS194
17	1b6b	A	SER97, LEU111, HIS122, LEU124, TYR168
18	1b73	A	ASP7, SER8, CYS70, CYS178
19	1b93	A	HIS19, GLY66, ASP71, ASP91, HIS98, ASP101
20	1bg0	A	ARG126, GLU225, ARG229, ARG280, ARG309
21	1bml	A	HIS603, ASP646
22	1bol	A	HIS46, GLU105, HIS109
23	1brm	A	CYS135, GLN162, HIS274
24	1brw	A	HIS82, ARG168, SER183, LYS187
25	1bs4	A	GLY45, GLN50, LEU91, GLU133
26	1btl	A	SER70, LYS73, SER130, GLU166
27	1bwd	A	ASP108, ARG127, ASP179, HIS227, ASP229, HIS331, CYS332
28	1bwp	A	SER47, GLY74, ASN104, ASP192, HIS195
29	1bzy	A	GLU133, ASP134, ASP137, LYS165, ARG169
30	1c3j	A	GLU22, ASP100
31	1cb8	A	HIS225, TYR234, ARG288
32	1cd5	A	ASP72, ASP141, HIS143, GLU148
33	1chd	A	SER164, THR165, HIS190, MET283, ASP286
34	1chk	A	GLU22, ASP40
35	1chm	A	HIS232, GLU262, GLU358
36	1coy	A	GLU361, HIS447, ASN485
37	1cqq	A	HIS40, GLU71, GLY145, CYS147
38	1ctt	A	GLU104
39	1d0s	A	GLU317
40	1d4a	A	GLY149, TYR155, HIS161
41	1d4c	A	HIS364, ARG401, HIS503, ARG544
42	1daa	A	LYS145, GLU177, LEU201
43	1dae	A	THR11, LYS15, LYS37, SER41
44	1db3	A	THR132, GLU134, TYR156, LYS160
45	1dbt	A	ASP60, LYS62
46	1dco	C	HIS62, HIS63, HIS80, ASP89

47	1dgs	A	LYS116, ASP118, ARG196, LYS312
48	1dii	A	TYR95, GLU380, GLU427, HIS436, ARG474
49	1diz	A	TYR222, TRP272, ASP238
50	1dli	A	THR118, GLU145, LYS204, ASN208, CYS260, ASP264
51	1dnk	A	GLU78, HIS134, ASP212, HIS252
52	1do8	A	TYR112, LYS183, ASP278
53	1dqs	A	HIS275
54	1dzr	A	HIS63, ASP170
55	1e2a	A	HIS78, GLN80, ASP81, HIS82
56	1ebf	A	ASP219, LYS223
57	1ef8	A	HIS66, GLY110, TYR140
58	1eug	A	ASP64, HIS187
59	1eyi	A	ASP68, ASP74, GLU98
60	1fgh	A	ASP100, HIS101, HIS147, ASP165, HIS167, GLU262, SER642
61	1foh	A	ASP54, ARG281, TYR289
62	1fro	A	GLU172
63	1fua	A	GLU73
64	1fui	A	GLU337, ASP361
65	1g72	A	ASP297
66	1gim	A	ASP13, HIS41, GLN224
67	1gog	A	CYS228, TYR272, TRP290, TYR495
68	1gpm	A	GLY59, CYS86, TYR87, HIS181, GLU183, ASP239
69	1gpr	A	THR66, HIS68, HIS83, GLY85
70	1gtp	A	HIS112, HIS179
71	1hfs	A	GLU202, MET219
72	1hxq	A	CYS160, HIS164, HIS166, GLN168
73	1i7d	A	GLU7, LYS8, PHE328, ARG330
74	1ivh	A	GLU254
75	1jdw	A	ASP254, HIS303, CYS407
76	1kas	A	CYS163, HIS303, HIS340, PHE400
77	1kfu	L	GLN99, CYS105, HIS262, ASN286
78	1kra	C	HIS219, ASP221, HIS320, ARG336
79	1lba	A	LYS128
80	1lcb	A	GLU60, CYS198, SER219, ASP221, ASP257, HIS259
81	1lxa	A	HIS125
82	1mas	A	ASN168, HIS241
83	1mbb	A	ARG159, SER229, GLU325
84	1mhy	D	CYS151, THR213
85	1mla	A	SER92, HIS201, GLN250
86	1moq	A	GLU481, LYS485, GLU488, HIS504, LYS603
87	1mpy	A	HIS199, HIS246, TYR255
88	1nba	A	ASP51, LYS144, ALA172, THR173, CYS177
89	1nid	A	ASP98, HIS255
90	1nsp	A	ASN119, LYS16
91	1nzy	A	PHE64, HIS90, GLY114, TRP137, ASP145
92	1ofg	A	LYS129, TYR217
93	1pfk	A	GLY11, ARG72, THR125, ASP127, ARG171
94	1pjb	A	LYS74, HIS95, GLU117, ASP269
95	1ps1	A	PHE77, HIS309
96	1pud	A	ASP102
97	1pym	A	GLY47, LEU48, ASP58, LYS120
98	1qfe	A	GLU86, HIS143, LYS170

99	1qq5	A	ASP8, THR12, ARG39, ASN115, LYS147, SER171, ASN173, PHE175, ASP176
100	1qum	A	GLU261
101	1ra2	A	ILE5, MET20, ASP27, LEU28, PHE31, LEU54, ILE94
102	1rbl	A	LYS175, LYS177, LYS201, ASP203, HIS294, HIS327
103	1req	A	TYR89, HIS244, LYS604, ASP608, HIS610
104	1rpt	A	ARG11, HIS12, ARG15, ARG79, HIS257, ASP258
105	1tyf	A	GLY68, SER97, MET98, HIS122, ASP171
106	1uae	A	ASN23, CYS115, ASP305, ARG397
107	1uag	A	LYS115, ASN138, HIS183
108	1ula	A	HIS86, GLU89, ASN243
109	1uok	A	ASP199, GLU255, ASP329
110	1vao	A	TYR108, ASP170, HIS422, TYR503, ARG504
111	1vnc	A	LYS353, HIS404
112	1xva	A	GLU15
113	1ytw	A	GLU290, ASP356, HIS402, CYS403, ARG409, THR410
114	1zio	A	LYS13, ARG127, ARG160, ASP162, ASP163, ARG171
115	2acy	A	ARG23, ASN41
116	2adm	A	ASN105, PRO106, TYR108
117	2alr	A	TYR49, LYS79
118	2bbk	L	TYR119, ASP76, THR122, TRP108, ASP32
119	2cpo	A	HIS105, GLU183
120	2hdh	A	SER137, HIS158, GLU170, ASN208
121	2hgs	A	ARG125, SER151, GLY369, ARG450
122	2jcw	A	HIS63, ARG143
123	2pda	A	GLU64
124	2pfl	A	TRP333, CYS418, CYS419, GLY734
125	2phk	A	ASP149, LYS151
126	2plc	A	HIS45, ASP46, ARG84, HIS93, ASP278
127	2thi	A	CYS113, GLU241
128	3csm	A	ARG16, ARG157, LYS168, GLU246
129	3eca	A	THR12, TYR25, THR89, ASP90, LYS162
130	3pca	M	TYR447, ARG457
131	4kbp	A	HIS202, HIS295, HIS296
132	5cox	A	GLN203, HIS207, TYR385
133	5enl	A	GLU168, GLU211, LYS345, HIS373
134	5fit	A	GLN83, HIS94, HIS96
135	8tln	E	GLU143, HIS231
136	9pap	A	ASN175, GLN19, HIS159
137	1dl2	A	GLU132, ARG136, ASP275, GLU435
138	1r51	A	ARG176, GLN228
139	1dos	A	ASP109, GLU182, ASN286
140	2jqx	A	ASP270, GLU272, ARG338, ASP631
141	3kyh	A	ARG393, GLU433, LYS456, ARG458
142	1cdd	A	ASN106, HIS108, SER135, ASP144
143	3pep	A	ASP32, SER35, TYR75, ASP215
144	1pnf	A	ASP60, GLU206
145	1aqf	A	ARG72, ARG119, LYS269, THR327, SER361, GLU363
146	1qae	A	ARG87, HIS89, ASN119
147	1huk	A	ASP117
148	1kr3	A	ASP103, ASN193

Average (Standard deviation) number of catalytic residues = 3.38 (1.56)

PW79 dataset (79 proteins)

S.No.	PDB	Chain	Catalytic site residues
1	1a26	A	TYR907, GLU988
2	1a4i	A	LYS56
3	1a4s	A	ASN166, GLU263, CYS297
4	1ae7	A	GLY30, HIS48, ASP99
5	1afw	A	CYS125, HIS375, CYS403, GLY405
6	1ah7	A	ASP55
7	1akm	A	ARG106, HIS133, GLN136, ASP231, CYS273, ARG319
8	1aop	A	ARG83, ARG153, LYS215, LYS217, CYS483
9	1apx	A	ARG38, HIS42, ASN71
10	1apy	B	THR183, THR201, THR234, GLY235
11	1aq2	A	HIS232, LYS254, ARG333
12	1aw8	B	TYR58
13	1b3r	A	ASP130, LYS185, ASP189, ASN190, CYS194
14	1b93	A	HIS19, GLY66, ASP71, ASP91, HIS98, ASP101
15	1bol	A	HIS46, GLU105, HIS109
16	1brm	A	CYS135, GLN162, HIS274
17	1bs4	A	GLY45, GLN50, LEU91, GLU133
18	1btl	A	SER70, LYS73, SER130, GLU166
19	1bzy	A	GLU133, ASP134, ASP137, LYS165, ARG169
20	1cd5	A	ASP72, ASP141, HIS143, GLU148
21	1chd	A	SER164, THR165, HIS190, MET283, ASP286
22	1ctt	A	GLU104
23	1d4a	A	GLY149, TYR155, HIS161
24	1daa	A	LYS145, GLU177, LEU201
25	1dae	A	THR11, LYS15, LYS37, SER41
26	1db3	A	THR132, GLU134, TYR156, LYS160
27	1dbt	A	ASP60, LYS62
28	1dco	C	HIS62, HIS63, HIS80, ASP89
29	1diz	A	TYR222, ASP238, TRP272
30	1dj0	A	ASP60
31	1dnk	A	GLU78, HIS134, ASP212, HIS252
32	1dnp	A	TRP306, TRP359, TRP382
33	1dqs	A	HIS275
34	1dzr	A	HIS63, ASP170
35	1e2a	A	HIS78, GLN80, ASP81, HIS82
36	1ef8	A	HIS66, GLY110, TYR140
37	1eyi	A	ASP68, ASP74, GLU98
38	1fua	A	GLU73
39	1gim	A	ASP13, HIS41, GLN224
40	1gpm	A	GLY59, CYS86, TYR87, HIS181, GLU183, ASP239
41	1gpr	A	THR66, HIS68, HIS83, GLY85
42	1hxq	A	CYS160, HIS164, HIS166, GLN168
43	1iph	A	HIS128, SER167, ASN201
44	1jdw	A	ASP254, HIS303, CYS407
45	1kas	A	CYS163, HIS303, HIS340, PHE400
46	1kra	C	HIS219, ASP221, HIS320, ARG336
47	1lba	A	TYR46, LYS128
48	1lxa	A	HIS125
49	1mbb	A	ARG159, SER229, GLU325

50	1mek	A	CYS36, GLY37, HIS38, CYS39
51	1mla	A	SER92, HIS201, GLN250
52	1moq	A	GLU481, LYS485, GLU488, HIS504, LYS603
53	1mpy	A	HIS199, HIS246, TYR255
54	1nba	A	ASP51, LYS144, ALA172, THR173, CYS177
55	1nsp	A	ASN119, LYS16
56	1pfk	A	GLY11, ARG72, THR125, ASP127, ARG171
57	1pjb	A	LYS74, HIS95, GLU117, ASP269
58	1pnl	B	SER1, ALA69, ASN241
59	1pud	A	ASP102
60	1qfe	A	GLU86, HIS143, LYS170
61	1uae	A	ASN23, CYS115, ASP305, ARG397
62	1ula	A	HIS86, GLU89, ASN243
63	1uok	A	ASP199, GLU255, ASP329
64	1r51	A	ARG176, GLN228
65	1xva	A	GLU15
66	2acy	A	ARG23, ASN41
67	2alr	A	TYR49, LYS79
68	2bbk	L	TYR119, ASP76, THR122, TRP108, ASP32
69	2cpo	A	HIS105, GLU183
70	2hgs	A	ARG125, SER151, GLY369, ARG450
71	2jcw	A	HIS63, ARG143
72	2pfl	A	TRP333, CYS418, CYS419, GLY734
73	2plc	A	HIS45, ASP46, ARG84, HIS93, ASP278
74	3eca	A	THR12, TYR25, THR89, ASP90, LYS162
75	1cul	B	ARG1029
76	1dos	A	ASP109, GLU182, ASN286
77	1cdd	A	ASN106, HIS108, SER135, ASP144
78	1qae	A	ARG87, HIS89, ASN119
79	1huk	A	ASP117

Average (Standard deviation) number of catalytic residues = 3.19 (1.34)

EF-fold-164 dataset (164 proteins)

S.No.	PDB	Chain	Catalytic site residues
1	1a26	A	TYR907, GLU988
2	1a41	A	TYR274, ARG223, HIS265, LYS167
3	1a4i	A	LYS56
4	1a4y	B	HIS13, LYS40, HIS114
5	1a6d	A	THR97, ASP390, THR96, ASP63
6	1a79	A	LYS156, TYR115, HIS125
7	1aa6	A	ARG333, HIS141, LYS44
8	1ab4	A	TYR122, ARG32, HIS78
9	1adn	A	CYS69
10	1af7	A	ARG98, ASP154
11	1afr	A	THR199, ASP228, TRP62, HIS146
12	1afw	A	HIS375, CYS403, CYS125, GLY405
13	1ak0	A	ARG48
14	1ako	A	HIS259, ASP229, ASN7, ASP151, ASN153
15	1am2	A	HIS75, ASN198, ASN74, SER1, HIS197, THR72
16	1aop	A	LYS215, ARG83, CYS483, LYS217, ARG153
17	1aui	A	ASP121, HIS151
18	1auk	A	LYS302, LYS123, HIS229, HIS125, ASP281, SER150
19	1b5q	A	GLU62
20	1b65	A	ASN218, GLY289, TYR146, SER288, SER250
21	1b93	A	HIS98, ASP71, HIS19, ASP101, ASP91, GLY66
22	1bd3	A	THR141, ARG137, ASP235
23	1bfd	A	GLU28, HIS281, HIS70
24	1bg6	A	ASP297, HIS202
25	1bmt	A	SER810, ASP757, HIS759
26	1bo1	A	ASP278, LYS150
27	1bob	A	GLU255
28	1bol	A	HIS109, HIS46, GLU105
29	1bou	B	HIS195
30	1brw	A	LYS187, SER183, HIS82, ARG168
31	1bwd	A	ASP179, ASP229, HIS331, ARG127, CYS332, ASP108, HIS227
32	1c4x	A	HIS263, SER110, ASP235
33	1c4z	A	GLU550, ARG506, CYS820, HIS818, GLU539, ASP607
34	1cb8	A	TYR234, HIS225, ARG288
35	1cf2	O	CYS140, HIS219
36	1cfr	A	LYS190
37	1cg2	A	GLU175, GLU200, HIS112, ASP141, HIS385, GLU176
38	1chd	A	ASP286, SER164, MET283, THR165, HIS190
39	1chk	B	GLU22, ASP40
40	1chm	A	GLU262, GLU358, HIS232
41	1cjy	A	SER228, GLY198, GLY197, ASP549
42	1cqq	A	GLY145, HIS40, GLU71, CYS147
43	1d1q	A	ALA13, ARG19, SER20, ASP132
44	1d4a	A	TYR155, HIS161, GLY149
45	1d5r	A	ASP92, ARG130, CYS124
46	1dci	A	ASP204, GLU196
47	1dgs	A	ASP118, LYS116, LYS312, ARG196
48	1di1	A	TYR92, PHE112, TRP333, PHE178
49	1dj0	A	ASP60

50	1dki	A	SER47, HIS195
51	1dl5	A	SER59
52	1dnp	A	TRP306, TRP359, TRP382
53	1dqs	A	HIS275
54	1e0c	A	HIS234, GLN231, HIS233, ARG235, THR232
55	1el9	A	HIS269, LYS348, LYS265, CYS315, THR48
56	1ecf	A	GLY102, CYS1, ASN101
57	1eej	A	CYS98, TYR100, CYS101, ARG125
58	1eg7	A	LYS1074
59	1et0	A	LYS159, THR38, GLU193
60	1eu1	A	TYR114, TRP116
61	1su4	A	ASP351
62	1f6d	A	GLU131, ASP95, GLU117, HIS213
63	1f75	A	ARG42, ARG33, ARG197, ARG203
64	1f7l	A	LYS62, HIS105
65	1f7u	A	LYS156, HIS162, HIS159
66	1fft	F	HIS419, LYS362, ASP135, THR211, THR359, SER315, SER299, ASN142, TYR61, THR201, SER145, MET79, ASN124, PHE420, PHE103, GLU286, THR149, HIS284, HIS421, THR204, ASP75, ARG71, TYR288
67	1foh	A	ASP54, ARG281, TYR289
68	1fr2	B	HIS102, HIS103, GLU100, ARG5
69	1fui	A	GLU337, ASP361
70	1g0d	A	CYS272, ASP355, HIS332, TYR515
71	1g24	A	GLU214
72	1g72	A	ASP297
73	1g8f	A	ARG290
74	1ga8	A	GLN189, ASP130, ASN153
75	1gpj	A	SER48, HIS84
76	1gpm	A	HIS181, GLU183, TYR87, CYS86, GLY59, ASP239
77	1gpr	A	GLY85, HIS68, HIS83, THR66
78	1gq8	A	ASP136, GLN113, GLN135, ASP157
79	1gsa	A	ARG225, LYS160, ARG210
80	1gt7	A	GLU117, GLU171
81	1h3i	A	TYR335, HIS293
82	1h4g	B	GLU184, GLU94
83	1h54	A	GLU487
84	1hfe	M	LYS237, CYS178
85	1hr6	B	GLU73
86	1hrk	A	GLU343, HIS263, HIS341
87	1i19	A	GLU311, GLU475, ARG477
88	1i6p	A	ASP44, ARG46
89	1i78	A	HIS212, ASP85, ASP83, ALA99, ASP210
90	1i8d	A	HIS102, CYS48, MET64, PHE2, SER41
91	1i8t	B	ASP348, ARG278, GLU298, ARG170
92	1inp	A	LYS37, THR158
93	1itx	A	GLU204, ASP200
94	1j09	A	LYS246
95	1jch	A	ASP510, HIS513, ARG545, GLU517
96	1jfl	A	CYS82, CYS194
97	1jh6	A	HIS42, SER121, MET117, HIS119, TYR124
98	1jhf	A	MET118, GLU152, LYS156, SER119, ASP127

99	1jm6	A	HIS1239, GLU1243
100	1jms	A	ASP434
101	1k30	A	HIS139, ASP144
102	1k32	A	SER965, HIS746, ASP966, GLY918
103	1k4t	A	HIS632, ARG488, ARG590
104	1k82	A	GLU2, LYS56, PRO1, ARG258
105	1kl7	A	LYS124
106	1kny	A	GLU145, LYS149
107	1kyq	A	ASP141
108	1kzh	A	THR204, ASP206, GLY82, LYS203, ARG146
109	1l0o	A	GLU46, ARG105
110	1l1d	A	HIS480, ARG493, CYS495, CYS440, ASP484
111	1lam	A	LYS262, ARG336, ASP255
112	1lba	A	TYR46, LYS128
113	1lbu	A	HIS192
114	1lci	A	HIS245, ARG218, LYS529, THR343
115	1ldm	A	HIS193, ASP166, ARG169
116	1m6k	A	SER67
117	1moq	A	LYS603, LYS485, GLU488, GLU481
118	1mpx	A	TYR175, HIS340, SER174, TYR82, ASP307
119	1mpy	A	HIS199, HIS246, TYR255
120	1muc	A	LYS169, LYS167, GLU327
121	1mug	A	ASN18
122	1nf9	A	ASP38
123	1nvm	A	TYR291, HIS21
124	1nww	A	TYR53, ARG99, ASP101, ASN55, ASP132
125	1o04	A	SER302, LYS192, GLU268, GLU399
126	1o8a	A	GLU384, HIS353, HIS513, ALA354, TYR523
127	1o98	A	SER62, ASP154, ARG261
128	1oe8	A	TYR10
129	1otg	A	PRO2, PHE35, ARG41
130	1p3d	A	LYS129
131	1pmi	A	GLN111, ARG304, GLU294
132	1pvd	A	ASP28, HIS114, HIS115, GLU477
133	1pym	A	ASP58, LEU48, GLY47, LYS120
134	1qba	A	GLU540, ASP539
135	1qf6	A	ARG363
136	1qfm	A	ASP641, SER554, HIS680
137	1qh5	A	ASP58
138	1qho	A	ASP228, ASP329, GLU256, HIS132
139	1qi9	A	HIS411, HIS418, HIS486, LYS341
140	1qmh	A	HIS309
141	1qq5	A	ARG39, THR12, ASP176, PHE175, ASP8, ASN173, LYS147, ASN115, SER171
142	1qtn	A	GLY350, HIS317, CYS360, ARG258
143	1rtu	A	GLU62, HIS101, HIS41
144	1tml	A	ASP117, ASP265
145	1uaq	A	GLU64, CYS91, SER89
146	1ush	A	ARG375, ASP120, ARG410, HIS117, ARG379, ASN116
147	1xva	A	GLU15
148	1zym	A	HIS189, THR168
149	2abk	A	ASP138, LYS120

150	2apr	A	THR221, SER38, ASP35, ASP218
151	2bbk	L	TYR119, ASP76, THR122, TRP108, ASP32
152	2pgd	A	ASN187, LYS183, GLY130, GLU190
153	2pia	A	CYS199, ASN44, SER58, GLU223
154	3mdd	A	GLU376
155	7odc	A	GLU274, LYS69, HIS197
156	1dl2	A	ARG136, GLU435, ASP275, GLU132
157	1sox	A	ARG138, TYR322
158	1cef	A	ASN161, SER62, LYS65, TYR159, HIS298
159	2w0w	A	ARG18
160	1xje	A	CYS322, CYS333, ASN320, GLU324, LYS40
161	2vya	A	LYS142, SER218, ILE238, SER241, GLY240, GLY239, SER217
162	1dyu	A	ASP383
163	1pnf	A	ASP60, GLU206
164	1q23	A	ASP197, HIS193, ARG18

Average (Standard deviation) number of catalytic residues = 3.14 (2.21)

Macie-254 dataset (254 proteins)

S.No.	PDB	Chain	Catalytic site residues
1	12as	A	ASP46, ARG100, GLN116
2	13pk	A	ARG39, LYS219, GLY376, GLY399
3	1a0i	A	LYS34, LYS238, LYS240
4	1a26	A	TYR907, GLU988
5	1a4s	A	ASN166, GLU263, CYS297
6	1a7u	A	ASP228, HIS257, SER98, MET99, PHE32
7	1adn	A	CYS38
8	1afr	A	TRP62, HIS146, THR199, ASP228
9	1afw	A	CYS125, HIS375, CYS403, GLY405
10	1ah7	A	ASP55
11	1ako	A	ASN7, ASP151, ASN153, ASP229, HIS259
12	1al6	A	SER244, HIS274, HIS320, ASP375
13	1alk	A	SER102, ARG166
14	1amo	A	SER457, CYS630, ASP675
15	1aq2	A	HIS232, LYS254, ARG333
16	1auk	A	ASP281, SER150, LYS123, LYS302, HIS125, HIS229, ARG73
17	1b3r	A	HIS54, ASP130, LYS185, ASP189, ASN190, CYS194, HIS300, SER360
18	1b57	A	ASP109, GLU182, ASN286
19	1b6b	A	SER97, LEU111, HIS122, LEU124, TYR168
20	1b6t	A	ARG91, LYS42, HIS18, SER129
21	1ba3	A	THR343, LYS443, LYS529
22	1bc2	A	ASP90, ASN180
23	1bg0	A	ARG126, GLU225, ARG229, CYS271, THR273, ARG280, ARG309
24	1bmt	A	HIS759, ASP757, SER810
25	1bob	A	GLU255
26	1brw	A	HIS82, ARG168, SER183, LYS187
27	1bs1	A	LYS15, LYS37, SER41
28	1bsz	A	GLY45, GLN50, LEU91, GLU133
29	1bt3	A	PHE261
30	1btl	A	SER70, LYS73, SER130, GLU166, ALA237
31	1bwp	A	SER47, GLY74, ASN104, ASP192, HIS195
32	1bxk	A	TYR160, LYS164, THR134, ASP135, GLU136
33	1bzy	A	GLU133, ASP134, ASP137
34	1c0p	A	SER1335, ASN1054, GLN1339
35	1c9u	A	HIS144, ASP163, ARG228
36	1ca2	A	HIS64, GLU106, THR199
37	1cdg	A	ARG227, ASP229, GLU257, HIS327, ASP328
38	1cg6	A	ASP220
39	1chm	A	HIS232, GLU262, GLU358
40	1cju	B	ARG1029
41	1ct9	A	ALA1, GLY75, LEU50, ASN74, THR322, ALA325
42	1ctt	A	GLU104
43	1d0s	A	GLU317
44	1d4a	A	GLY149, TYR155, HIS161
45	1d6m	A	GLU7, LYS8, TYR328, ARG330
46	1d8c	A	ASP270, GLU272, ARG338, ASP631
47	1daa	A	TYR31, LYS145, GLU177, LEU201
48	1dci	A	ASP176, GLU196, ASP204
49	1dd8	A	CYS163, HIS298, LYS328, HIS333, PHE390, PHE392

50	1dea	A	ASP72, ASP141, HIS143, GLU148
51	1dhp	A	LYS161, TYR133, ILE203
52	1di1	A	TYR92, PHE112, PHE178, LYS206, TRP333
53	1djx	A	HIS311, ASN312, HIS356, GLU390
54	1dl2	A	GLU132, ARG136, ASP275
55	1dli	A	THR118, GLU145, LYS204, ASN208, CYS260, ASP264
56	1dnk	A	TYR76, GLU78, HIS134, ASP212, HIS252
57	1dnp	A	GLU274, TRP277, ASN341
58	1do8	A	TYR112, ARG165, LYS183, ASP278, ASN421
59	1doc	A	HIS72, TYR201, PRO293, LYS297, TYR385
60	1dpx	A	ASP52, GLU35
61	1dqs	A	LYS152, LYS250, GLU260, ARG264, ASN268, HIS275
62	1dty	A	ASP245, TYR17, TYR144, LYS274
63	1e4e	A	HIS99, VAL19, GLY311, ARG290, TYR315, LYS22, GLU250, HIS244
64	1e6e	A	HIS55, ASP159, ILE376, THR377
65	1e6u	A	TYR136, LYS140, SER107, SER108, CYS109, HIS179
66	1ea0	A	CYS1, ASN231, GLY232
67	1ecf	A	CYS1, ASN101, GLY102, GLY27, TYR258
68	1ef8	A	HIS66, GLY110, TYR140
69	1eh6	A	GLU172, HIS146, CYS145, TYR114, ASN137
70	1et0	A	THR38, LYS159, GLU193
71	1eug	A	PHE77, TYR66, HIS187
72	1ey3	A	GLU144, GLU164, GLY141, ALA98
73	1eyq	A	THR48, TYR106
74	1ezf	A	TYR171, ARG228, ARG218, PHE288
75	1f2v	A	HIS43
76	1f61	A	SER315, SER317, HIS193, CYS191, ARG228, HIS180
77	1f7u	A	HIS159, LYS156, HIS162
78	1fbx	A	HIS112, HIS179
79	1fcb	A	TYR254, ASP282, HIS373
80	1fua	A	GLU73
81	1fui	A	GLU337, ASP361
82	1fva	A	CYS72, TYR103, GLU115, TYR155, CYS218, CYS227
83	1fwj	C	ASP221, ASP360, HIS219, HIS320, ARG336
84	1g72	A	ASP297
85	1g8f	A	ARG197, THR196, HIS201
86	1ggt	A	TRP279, CYS314, HIS373, ASP396, TYR560
87	1gim	A	ASP13, HIS41, GLN224, LYS16
88	1gog	A	TYR495, HIS581, TYR272, HIS496, CYS228
89	1gpb	A	LYS680, HIS377
90	1gpm	B	LYS381, ASP239, GLU183, HIS181, CYS86, GLY59, TYR87
91	1gsa	A	LYS160, ARG210, ARG225
92	1gtk	A	ASP84, LYS83, ARG131, ARG132, CYS242
93	1gu1	A	PRO15, ASN16, ARG23, TYR28, ASN79, ALA82, GLU104, HIS106, ARG113
94	1gzg	A	LYS205, LYS260
95	1h1z	A	ASP178, ASP38, SER11
96	1h3b	A	ASP376, HIS451, TYR495, PHE365, TYR420, TRP489, TYR609, PHE601, PHE605, TRP169, GLU45
97	1h3i	A	TYR335, TYR245, HIS297, HIS293, TYR305
98	1hfe	L	GLU156, GLU159, CYS178, SER198, LYS237, GLU240, GLU245
99	1hpl	A	SER152, HIS263, ASP176, PHE77, LEU153

100	1hs6	A	GLU271, ASP375
101	1hxq	A	ASN153, SER161, HIS164, HIS166, GLN168
102	1hyo	A	HIS133, GLU199, ARG237, GLN240, LYS253, GLU364
103	1hzd	A	ALA141, GLY186, GLU189, GLU209
104	1hzy	A	ASP233, HIS254, ASP301
105	1idt	A	LYS14, LYS74
106	1igs	A	LYS53, GLU51, LYS110, GLU159, ASN180, SER211
107	1ile	A	LYS591, LYS594, PRO46, TRP518, TRP558, ASP85, GLN554
108	1ir3	A	ASP1132, ARG1136
109	1itq	A	HIS152, ASP288
110	1ivh	A	MET135, SER136, GLU254
111	1j09	A	LYS246
112	1j21	A	SER173, ARG92
113	1jc5	A	GLU48, GLU141
114	1jdw	A	ASP170, ASP254, HIS303, ASP305, CYS407
115	1jfg	B	TYR93, ARG182, LYS232, ARG304, TYR305, THR96, LEU97, ASP100
116	1kc7	A	ARG92, LYS22, HIS455, ARG337, MET103, GLY101, TYR851, SER764, CYS831
117	1kei	A	GLU143, TYR157, ASP226, HIS231
118	1kq3	A	HIS255
119	1kzl	A	THR50, SER41, CYS48, THR148, HIS102
120	1l8s	A	GLY30, HIS48, TYR52, TYR73, ASP99
121	1lcb	A	GLU60, TYR146, CYS198, ARG218, ASP221, TRP82
122	1lij	A	ARG136, ASP318
123	1ljl	A	CYS10, THR11, GLY12, ASN13, SER14, CYS15, ARG16, SER17, CYS82, CYS89, ASP105
124	1lok	A	GLU151
125	1luc	A	HIS44, HIS45
126	1lwy	A	LYS249, ASP268
127	1lxa	A	HIS125, GLY143, ASP126
128	1m4l	A	ARG127, GLU270
129	1m6k	A	TRP160, ALA215, SER67, SER115, SER120
130	1m9c	A	ARG55, PHE60, GLN63, ASN102, PHE113, LEU122, HIS126
131	1mb9	A	TYR348, GLU382, LYS443
132	1mek	A	CYS36
133	1mg5	A	TYR152, LYS156, SER139
134	1mht	A	CYS81, ARG163, ARG165, GLU119
135	1mki	A	LYS77, TYR253, TYR201, VAL271
136	1mla	A	SER92, LEU93, HIS201, ARG117, GLN250, GLN11
137	1mns	A	LYS164, LYS166, ASP270, HIS297, GLU317
138	1mpy	A	HIS199, HIS246, TYR255
139	1muc	A	LYS167, LYS169, GLU327
140	1mum	A	ASP58, CYS123, ARG158, GLU188, ASN210
141	1n1d	A	LYS44, LYS46
142	1n20	A	TRP323, PHE578, ILE344, VAL452
143	1n62	B	CYS388, GLU763
144	1nba	A	ASP51, LYS144, ALA172, THR173, CYS177
145	1ndp	B	HIS122, LYS16, TYR56, ASN119, GLU133
146	1nfs	A	CYS67, GLU116, TRP161, TYR104
147	1nzy	A	PHE64, ALA86, HIS90, GLY114, TRP137, ASP145
148	1o8a	A	HIS353, ALA354, GLU384, HIS513, TYR523
149	1odm	A	PHE211

150	1ofd	A	CYS1, ARG31, PHE207, ASN227, GLY228, GLU903, GLN969, LYS972, GLN978
151	1onr	A	ASP17, GLU96, LYS132, THR156
152	1onw	A	ASP285
153	1os7	A	ARG270
154	1oth	A	ARG141, HIS168, GLN171, ASP263, CYS303, ARG330
155	1oya	A	HIS191, ASN194, ASN251, TYR196, THR37
156	1p5d	X	LYS118, HIS109, HIS329, ARG20, ARG247
157	1pfq	A	TYR547, SER630, TYR631, ASP708, HIS740
158	1pjb	A	LYS74, TYR93, HIS95, GLU117
159	1pkn	A	ARG119, ARG72, LYS269, THR327
160	1pq5	A	HIS56, ASP99, GLN192, GLY193, ASP194, SER195
161	1ps1	A	PHE76, PHE77, ASN219, TRP308, HIS309
162	1ps9	A	GLU164, TYR166, ARG214, HIS252, GLN339
163	1ptd	A	HIS32, ASP33, ARG69, HIS82, ASP274
164	1pwh	A	LYS41, ALA222, CYS303
165	1pym	A	ARG159, SER46, LEU48, SER123, ASN122, HIS190
166	1q0n	A	ARG82, ARG92
167	1q3s	A	ASP64, THR97, THR98, ASP393
168	1qfe	A	GLU86, HIS143, LYS170
169	1qh5	A	ASP58
170	1qjd	A	HIS365, GLU378, ARG381, ARG402, HIS504, ARG544
171	1ql0	A	ARG57, HIS89, ASN119, GLU127
172	1qlh	A	SER48, HIS51
173	1qq5	A	ASP8, THR12, ARG39, ASN115, LYS147, SER171, ASN173, PHE175, ASP176
174	1qrr	A	TYR182, LYS186, THR145, HIS183, SER180
175	1qum	A	GLU261
176	1rdd	A	HIS124
177	1req	A	TYR89, HIS244, LYS604, ASP608, HIS610
178	1rhs	A	SER274, VAL251, LYS249, THR252, GLY250, ARG186, ARG248
179	1rql	A	ASP12, ALA45, MET49, LYS53, HIS56, ARG160
180	1ru4	A	ASN268, LYS273
181	1ruv	A	HIS12, LYS41, HIS119, PHE120, ASP121
182	1ryp	I	THR1, ASP17, ARG19, LYS33, GLY47, SER129, ASP166, SER169
183	1s6l	A	CYS96, ASP99, CYS159
184	1s9i	A	ASP194, ASP221
185	1sc9	A	ASP207, HIS235, SER80, LYS236, THR11
186	1sdw	A	HIS108, GLN170
187	1sg4	A	LEU66, GLY111, GLU136
188	1sml	A	ASP120, TYR191
189	1sox	A	TYR322
190	1szd	A	HIS135, ARG45, PHE44, ASP43, PRO42, ASP118, ASN116
191	1ti6	A	HIS144, ASP174, TYR404
192	1uag	A	LYS115, ASN138, HIS183
193	1ula	A	HIS86, GLU89, ASN243
194	1uw8	A	GLU162, ARG92
195	1v25	A	LYS439, TRP444
196	1v54	A	ASP91, TRP126, SER156, SER157, HIS240, GLU242, TYR244, SER255, HIS291, THR316, LYS319, ARG438
197	1v97	A	GLU802, ARG880, GLU1261
198	1vao	A	TYR108, ASP170, TYR503, ARG504, HIS422

199	1vas	A	THR2, ARG22, GLN23, ARG26
200	1vlb	A	GLU869
201	1vr7	A	SER63, GLU62, HIS68, CYS83
202	1w1o	A	HIS105, ASP169, GLU288
203	1wpo	A	SER132, HIS63, HIS157, SER134, ARG165, ARG166
204	1xel	A	SER124, TYR149, LYS153
205	1xva	B	GLU15
206	1xx2	A	SER64, TYR150, SER318, GLU272, LYS67, LYS315
207	1yq4	A	PHE130, LEU263, GLU266, GLN251, HIS253, HIS364, ARG408, ARG297
208	1yrc	A	ARG186, ASP251, THR252, CYS357, LEU358, GLY359
209	1yt3	A	ASP155, TYR151
210	1ytw	A	GLU290, ASP356, HIS402, CYS403, ARG409, THR410
211	1z9h	A	TYR107, CYS110, PHE112, CYS113
212	1ze1	A	ASP39, TYR67
213	1znb	A	ASN193
214	1zp3	A	SER26, GLU28, ASP120, HIS273
215	2a84	A	HIS44, HIS47, SER196, SER197, ARG198
216	2adm	A	ASN105, PRO106, TYR108
217	2ahj	B	ARG56
218	2b3i	A	HIS39, HIS85, MET90, CYS82
219	2bbk	L	TRP108, ASP32, ASP76
220	2c3m	A	THR31, GLU64, ARG114, ASN996
221	2c7v	A	ARG14, ASP161, TYR174
222	2cpo	A	CYS29, ASP106, HIS105, GLU183
223	2dd5	C	CYS128, SER132, SER108
224	2eua	A	LYS420, GLU416, GLU284, LYS190, GLU240
225	2f9r	A	HIS47, HIS12, GLY48, ASP34, ASN252, ASP233, ASP52, TRP230, LYS93
226	2frv	B	CYS530, ARG463, GLU18, CYS65, CYS533, CYS68, SER486, HIS72
227	2gb0	A	THR48, LYS265, HIS269, CYS315, LYS348
228	2gmh	A	ARG331, THR367, SER82, CYS553, CYS559, CYS556, CYS528
229	2gsa	A	ASP245, LYS273
230	2gtr	A	LEU66, LEU115, ASP146
231	2jcw	A	HIS63, ARG143
232	2mas	A	ASP10, ASN168, HIS241
233	2pan	A	VAL51, LEU421, LEU476, ILE479, VAL25
234	2pfl	A	TRP333, CYS418, CYS419, GLY734
235	2pz0	A	ASP239, HIS17, HIS59, ARG18, LYS121
236	2qf7	A	ASP549, ASP655
237	2qut	A	LYS146, SER300, TYR363, ASP33, LYS229, GLU187, GLU189
238	2rjr	A	TYR63, GLY70
239	2sns	A	ARG35, GLU43, ARG87
240	2tmd	A	TYR169, HIS172, CYS30
241	2toh	A	HIS331, SER395
242	2ts1	A	THR40, HIS45, HIS48, LYS82, ARG86, GLN173, LYS230, LYS233, THR234, ASP194
243	2vck	A	ASP105, ASP206
244	3bpt	A	GLY98, GLY146, GLU169, ASP177
245	3csm	A	ARG16, ARG157, LYS168, GLU198, GLU246
246	3dfr	A	TRP21, ASP26, THR116
247	3kb9	A	PHE198, PHE95, PHE96, ARG338

248	3pva	A	CYS1, ASN175, TYR82, ARG228
249	5cox	A	GLN203, HIS207, TYR385
250	5eat	A	ARG264, ARG441, THR401, THR402, THR403, TYR527, ASP525, TYR520, ASP444, TRP273
251	5fit	A	GLN83, HIS96, HIS98
252	7atj	A	HIS42, ARG38, ASN70
253	7enl	A	GLU211, LYS345, GLU168, LYS396
254	9pap	A	HIS159, ASN175, GLN19

Average (Standard deviation) number of catalytic residues = 3.96 (2.09)

csalit-688 dataset (688 proteins)

S.No.	PDB	Chain	Catalytic site residues
1	135l	A	ASP52, GLU35
2	1a0j	A	ASP102, GLY193, HIS57, SER195
3	1a16	A	GLU383, HIS243, HIS350, HIS361
4	1a2t	A	ARG35, ARG87
5	1a41	A	ARG223, HIS265, LYS167, TYR274
6	1a4i	A	GLN100, LYS56
7	1a4l	A	ASP295, GLU217, HIS238
8	1a4y	B	HIS114, HIS13, LYS40
9	1a65	A	CYS452, HIS451
10	1a69	A	ARG217, ASP204
11	1a6d	B	ASP391, ASP62, THR95, THR96
12	1a79	D	HIS125, LYS156, TYR115
13	1a8h	A	LYS297, LYS300
14	1a8q	A	ASP223, HIS252, MET95, SER94, TRP28
15	1a8s	A	ASP224, HIS253, SER94, THR95, TRP28
16	1a95	A	ASP88, ASP89, ASP92
17	1aam	A	ASP223, LYS258, TRP142
18	1ab4	A	ARG32, HIS78, TYR122
19	1ab8	A	ARG1029
20	1abr	A	ARG167, GLU164
21	1agm	A	GLU179, GLU400, TRP120
22	1agy	A	ASP175, GLN121, HIS188, SER120, SER42
23	1aj0	A	ARG255, LYS221
24	1aj8	A	ASP312, HIS223, HIS262, SER193
25	1ak0	A	ARG48
26	1akd	A	ASP251, THR252
27	1akm	A	ARG106, ARG319, ASP231, CYS273, GLN136, HIS133
28	1ald	A	ASP33, GLU187, GLU189, LYS146, LYS229, SER300, TYR363
29	1am2	A	ASN198, ASN74, HIS197, HIS75, SER1, THR72
30	1amp	A	GLU151
31	1amy	A	ASP179, ASP289, GLU204
32	1aop	A	ARG153, ARG83, CYS483, LYS215, LYS217
33	1apx	D	ARG38, ASN71, HIS42
34	1aq0	B	GLU232, GLU280, GLU288, LYS283
35	1aql	A	ALA108, ALA195, ASP320, GLY107, HIS435, SER194
36	1ar1	A	GLU278, LYS354
37	1arz	A	HIS159, LYS163
38	1ast	A	GLU93, TYR149
39	1asy	A	ARG325, ARG531, ASP342
40	1at1	A	ARG105, ARG54, HIS134, LYS84, THR55
41	1aug	A	ARG91, CYS144, GLU81, HIS168
42	1aui	A	ARG122, ARG254, ASP121, HIS151
43	1auo	A	ASP168, GLN115, HIS199, LEU23, SER114
44	1avq	B	LYS131
45	1aw8	E	TYR58
46	1ax4	A	ASP133, HIS458, TYR72
47	1ay4	B	ASP222, LYS258, TRP140
48	1azw	A	ASP266, GLY43, HIS294, SER110, TRP111
49	1azy	B	ARG171, HIS85, LYS190

50	1b04	B	ALA114
51	1b2m	A	ARG77, GLU58, HIS40, HIS92, PHE100, TYR38
52	1b2r	A	ALA301, SER80
53	1b5d	A	ASP179, CYS148, GLU60
54	1b5q	A	GLU62
55	1b65	E	ASN218, GLY289, SER250, SER288, TYR146
56	1b66	B	CYS42, GLU133
57	1b6g	A	ASP124, ASP260, HIS289
58	1b73	A	ASP7, CYS178, CYS70, SER8
59	1b7y	A	ALA314, ARG204
60	1b8f	A	GLU414, TYR280
61	1b8g	A	ASP230, LYS273, TYR145
62	1b93	B	ASP101, ASP71, ASP91, GLY66, HIS19, HIS98
63	1b9h	A	ASP159, LYS188, PHE88
64	1bbs	B	ASP215, ASP32, SER35
65	1bd3	A	ARG137, ASP235, THR141
66	1be1	A	ASP14, HIS16
67	1bf2	A	ASP375, ASP510, GLU435
68	1bfd	A	GLU28, HIS281, HIS70
69	1bg6	A	ASP297, HIS202
70	1bgl	H	GLU461, GLU537
71	1bh2	A	ARG178, GLN204, GLU43, THR181
72	1bhg	A	GLU451, GLU540, TYR504
73	1bib	A	ARG118, ARG317, LYS183
74	1bix	A	ASP210, ASP283, HIS309, TYR171
75	1bjo	A	ASP174, LYS198, TRP102
76	1bjp	B	PHE50, PRO1
77	1bo1	B	ASP278, LYS150
78	1bol	A	GLU105, HIS109, HIS46
79	1boo	A	ASP96, PRO54, SER53
80	1bou	B	HIS195
81	1bp2	A	ASP99, GLY30, HIS48
82	1bqc	A	ASN127, GLU128, GLU225, HIS196, TYR198
83	1brm	A	CYS135, GLN162, HIS274
84	1bs0	A	ASP204, GLU175, HIS133, LYS236
85	1bs4	C	GLN1050, GLU1133, GLY1045, LEU1091
86	1bs9	A	ASP175, GLN91, HIS187, SER90, THR13
87	1bt1	B	GLU236
88	1bu7	B	CYS400, PHE393, THR268
89	1bvv	A	GLU172, GLU78
90	1bvz	B	ASP325, ASP421, GLU354, TRP356
91	1bwd	B	ARG127, ASP108, ASP179, ASP229, CYS332, HIS227, HIS331
92	1bwl	A	ASN191, HIS194, THR37, TYR196
93	1bwz	A	CYS217, CYS73, GLU208, HIS159
94	1bxr	D	CYS269, HIS353
95	1bya	A	ASP101, GLU186
96	1bzc	A	ARG221, ASP181, CYS215, SER222
97	1c0k	A	SER1335
98	1c17	L	ASP61
99	1c2t	A	ASN106, ASP144, HIS108, SER135
100	1c3j	A	ASP100, GLU22
101	1c4t	C	HIS375, THR323

102	1c4x	A	ASP235, HIS263, SER110
103	1c4z	A	ARG506, ASP607, CYS820, GLU539, GLU550, HIS818
104	1c54	A	ARG65, GLU54, HIS85
105	1c82	A	ASN349, HIS399, TYR408
106	1cb7	B	GLU171
107	1cb8	A	ARG288, HIS225, TYR234
108	1cbg	A	ASN324, GLU183, GLU397
109	1cbx	A	ARG127, GLU270
110	1cd5	A	ASP141, ASP72, GLU148, HIS143
111	1cel	A	ASP214, GLU212, GLU217, HIS228
112	1cev	B	ASP126, GLU271
113	1cf2	R	CYS140, HIS219
114	1cfr	A	LYS190
115	1cg2	A	ASP141, GLU175, GLU176, GLU200, HIS112, HIS385
116	1cgk	A	ASN336, CYS164, HIS303, PHE215
117	1chd	A	ASP286, HIS190, MET283, SER164, THR165
118	1chk	A	ASP40, GLU22, THR45
119	1ci8	A	LYS78, SER75, TRP348, TYR181, VAL351
120	1cjy	A	ASP549, GLY197, GLY198, SER228
121	1ck7	A	ALA404
122	1cl1	A	TYR111
123	1cm0	A	CYS574, GLU570, ILE571, ILE637, LEU606, PHE563, PHE568, TYR640, VAL572
124	1cmx	A	ASP181, CYS90, GLN84, HIS166
125	1cns	A	GLU67, GLU89
126	1coy	A	ASN485, GLU361, HIS447
127	1cqg	A	ALA35, CYS32
128	1cqq	A	CYS147, GLU71, GLY145, HIS40
129	1ctn	A	GLU315
130	1cv2	A	ASP108, GLU132, HIS272, TRP109
131	1cvr	A	CYS244, GLU152, GLY212, HIS211
132	1cw0	A	ASP97, HIS69
133	1cwy	A	ASP293, ASP395, GLU340
134	1cz0	A	ARG61, CYS105, HIS98
135	1cz1	A	GLU192, GLU292
136	1czf	A	ASP180, ASP201, ASP202, HIS223
137	1d1q	B	ALA13, ARG19, ASP132, SER20
138	1d2r	A	LYS192, LYS195
139	1d2t	A	ARG183, ASP193, HIS150, HIS189
140	1d3g	A	LYS255, PHE149, SER215, THR218
141	1d4c	A	ARG401, ARG544, GLU377, HIS503, HIS504
142	1d5r	A	ARG130, ASP92, CYS124
143	1d6o	A	ASP37, ILE56, TYR82
144	1d8h	A	ARG393, LYS409, LYS456
145	1d8t	A	ASP21
146	1dak	A	LYS15, LYS37, SER41, THR11
147	1db3	A	GLU134, LYS160, THR132, TYR156
148	1dbf	A	ARG90
149	1dco	A	GLU58, GLU81, HIS62, HIS63, HIS80
150	1ddj	B	ALA741, ASP646, HIS603
151	1de3	A	GLU96, HIS137, HIS50
152	1de6	A	ASP302, HIS270, LYS236

153	1dek	A	ARG68, HIS206
154	1dgk	N	ASP657
155	1dhf	A	GLU30, LEU22
156	1dhr	A	LYS150, TYR146
157	1dii	A	ARG474, GLU380, HIS436, TYR473
158	1din	A	ASP171, CYS123, HIS202, ILE37, LEU124
159	1dio	L	ASP335, GLN296, GLU170, HIS143
160	1diz	A	ASP238, TRP272, TYR222
161	1dj0	B	ASP60
162	1dj1	A	ALA48, HIS52, TRP191
163	1djl	B	ARG925, TYR1006, TYR890
164	1dki	D	HIS195, SER47
165	1dl5	A	SER59
166	1dmu	A	LYS144
167	1do6	B	GLU14, LYS15
168	1dod	A	TYR201, TYR385
169	1dpg	B	ASP177, HIS240
170	1ds2	E	ASP102, GLY193, HIS57, SER195
171	1dub	E	ALA98, GLU144, GLU164, GLY141
172	1dup	A	ASP90, ASP92
173	1dve	A	ARG136, ASP140, GLY139, GLY143, HIS25, THR135, TYR58
174	1dwo	B	ASP208, HIS236, SER80, THR11
175	1dxe	A	ARG75, HIS50
176	1dzr	A	ASP170, HIS63
177	1e0c	A	ARG235, GLN231, HIS233, HIS234, SER236, THR232
178	1e19	B	LYS131, LYS215, LYS277
179	1e1a	A	GLU37, HIS287
180	1e2a	A	GLN80, HIS78, HIS82
181	1e2t	A	ARG64, CYS69, GLU39
182	1e3v	A	ASP100, ASP40, TYR16
183	1e5q	H	ASP126
184	1e7l	B	GLU65, HIS41, HIS43
185	1e7q	A	LYS140, ALA107, TYR136, HIS179, CYS109
186	1e94	D	GLY45, LYS33, SER124, THR1
187	1eb6	A	GLU129, TYR106
188	1ebf	A	ASP219, LYS223
189	1ec9	A	ASN237, HIS339, LYS205, LYS207
190	1ecl	A	ARG321, GLU9, TYR319
191	1eej	A	ARG125, CYS101, CYS98, TYR100
192	1eh5	A	ASP233, GLN116, HIS289, MET41, SER115
193	1ei5	A	ASN155, HIS287, LYS65, SER62, TYR153
194	1els	A	HIS159, LYS396
195	1emd	A	ASP150, HIS177
196	1eq2	A	LYS178, SER116, TYR140
197	1esc	A	ASN106, GLY66, HIS283, SER14, TRP280
198	1eso	A	HIS61
199	1eu1	A	TRP116, TYR114
200	1euu	A	ASP92, GLU260, TYR370
201	1euy	A	ARG260, GLU34, LYS270
202	1evy	A	LYS210, THR267
203	1ex1	A	ASP285, GLU491
204	1exn	A	ARG86, LYS83

205	1exp	A	ASN169, ASP235, GLU127, GLU233, HIS205
206	1eyi	A	ASP68, ASP74, GLU98
207	1eyp	A	LYS97, THR48, TYR106
208	1ez1	B	ARG362, ASP286, GLY162, SER160, THR287
209	1ez2	A	ASP233, ASP301, HIS254
210	1f2d	A	LYS51, TYR295, TYR269
211	1f48	A	GLY18, LYS21
212	1f6d	A	ARG215, ASP95, GLU117, GLU131, HIS213, HIS246
213	1f75	A	ASP29, HIS46
214	1f7l	A	HIS105, LYS62
215	1f8m	A	ARG228, CYS191, HIS180, HIS193
216	1f8r	A	HIS223, LYS326
217	1f8x	A	ASP72, ASP92, GLU98, TYR7
218	1fa0	A	LYS215
219	1fc4	B	HIS213, LYS244
220	1fcq	A	ASP111, GLU113, TRP301, TYR184, TYR227
221	1ff3	A	CYS198, CYS206, GLU94
222	1fft	A	ARG71, ASN124, ASN142, ASP135, ASP75, GLU286, HIS284, HIS419, HIS421, LYS362, MET79, PHE103, PHE420, SER145, SER299, SER315, THR149, THR201, THR204, THR211, THR359, TYR288, TYR61
223	1fgh	A	ARG447, ARG644, ASP100, ASP165, HIS101, SER642
224	1fgj	A	ASP267, HIS233, HIS268, TYR334, TYR467
225	1fhl	A	ARG45, GLU136, GLU246
226	1fiq	C	ARG880, GLU1261, GLU802
227	1fnb	A	CYS272, GLU312, SER96
228	1foa	A	ASP291
229	1foh	A	ARG281, ASP54, PRO364, TYR289
230	1fps	A	ARG126, PHE253
231	1fq0	A	ARG49, GLU45, LYS133
232	1fr2	B	ARG5, GLU100, HIS102, HIS103
233	1fr8	A	ASP252, ASP318, GLU317
234	1fro	A	GLU172, GLU99
235	1fwk	D	THR183
236	1fy2	A	ALA121, GLU192, GLY88, HIS157, SER120
237	1g0d	A	ASP355, CYS272, HIS332, TYR515
238	1g24	A	GLU214
239	1g64	A	LYS41, THR43
240	1g6t	A	ASP313, GLU341, HIS385, LYS411
241	1g79	A	ARG197
242	1g8o	A	GLU317
243	1g8p	A	ARG289
244	1g99	A	ARG241, ARG91, ASP148, GLY212, SER10
245	1ga8	A	ASP130, ASP188, GLN189
246	1gal	A	HIS516
247	1gcb	A	ASN392, CYS73, GLN67, HIS369
248	1gcu	A	ARG171, GLU123, GLU126, GLU96, SER170, TYR97
249	1gdh	A	GLU269, HIS287
250	1ge7	B	GLU118, TYR133
251	1geq	A	ASP47, GLU36, TYR161
252	1glo	A	ASN184, GLN19, HIS164, SER25
253	1goj	A	GLY238
254	1gp1	A	GLN80, TRP158

255	1gp5	A	LYS213
256	1gpa	D	ARG569, LYS568, LYS574, THR676
257	1gpj	A	HIS84, SER48
258	1gpr	A	HIS68, HIS83, THR66
259	1gq8	A	ASP136, ASP157, GLN113, GLN135
260	1gqg	C	GLU73
261	1gt7	P	GLU117, GLU171
262	1gtp	S	HIS112, HIS179
263	1guf	B	TYR79
264	1gxs	A	SER158, TRP270
265	1gz6	D	LYS168, TYR164
266	1h19	A	GLN271, GLU296, TYR383
267	1h4g	B	GLU184, GLU94
268	1h54	B	GLU487
269	1h7a	A	ALA580, ASN311, CYS290, CYS79, GLU446
270	1h7o	A	LYS210, LYS263
271	1h7x	D	ALA671
272	1hdh	A	ARG55, ASP317, HIS115, HIS211, LYS113, LYS375
273	1hka	A	ARG82, ARG92
274	1hpm	A	LYS71
275	1hqc	A	ARG205, THR146
276	1hr6	A	ASP65
277	1hr7	H	GLN73
278	1hrd	A	ASP165, LYS125
279	1hrk	A	ARG164, ASP340, GLU343, GLU347, HIS263, TYR165
280	1hti	A	ASN11, GLU165, HIS95, LYS13
281	1hto	X	ARG339, ASP50, GLU327
282	1hv9	B	ARG18
283	1hy3	A	HIS107, LYS47, SER137
284	1hzf	A	CYS991, GLN994
285	1i19	B	ARG477, GLU311, GLU475
286	1i1e	A	ARG369, GLU267, TYR372
287	1i1i	P	GLU503, TYR613
288	1i6p	A	ARG46, ASP44
289	1i78	A	ALA99, ASP210, ASP83, ASP85, HIS212
290	1i8d	A	CYS48, HIS102, MET64, PHE2, SER41
291	1i8t	A	ARG170, ARG278, ASP348, GLU298
292	1i9a	B	CYS1067, GLU1087, GLU1116, TRP1161
293	1idj	A	ARG176, ARG236, ASP154, LYS239
294	1ig8	A	ARG173, ASP211, SER158
295	1im5	A	ALA129, ASP10, CYS133, LYS94
296	1ima	A	GLU70, THR95
297	1iph	A	ASN201, HIS128, HIS392
298	1it4	A	ASP85, HIS64
299	1itx	A	ASP200, GLU204
300	1iu4	D	ASP255, CYS64, HIS274, LYS269, TRP272
301	1iyd	B	LYS159
302	1j00	A	ASN73, ASP154, GLY44, HIS157
303	1j2u	A	GLU122, HIS178
304	1j49	A	ARG236, ASP260, GLU265, HIS297
305	1j53	A	GLU14, GLU61, HIS162
306	1j70	C	ARG197, HIS201, HIS204

307	1j79	B	ASP250
308	1j7g	A	GLN78, PHE79, THR80
309	1jch	C	ARG545, ASP510, GLU517, HIS513
310	1jen	A	CYS82
311	1jfl	A	CYS194, CYS82
312	1jh6	A	HIS119, HIS42, MET117, SER121, TYR124
313	1jhf	A	ASP127, GLU152, LYS156, MET118, SER119
314	1jkm	A	ASP308, HIS338, SER202
315	1jm6	B	GLU1243, HIS1239
316	1jms	A	ASP434
317	1jnr	C	ARG265, ASN74, ASP361, GLU141, HIS398, SER449, TRP234
318	1joa	A	ARG303, HIS10
319	1jof	E	ARG196, ARG274, GLU212, HIS148
320	1jrp	D	ARG310, GLU730
321	1js4	B	ASP55, ASP58, GLU424
322	1jxh	B	GLY210, LYS176
323	1k0w	A	ASN120
324	1k30	A	ASP144, HIS139
325	1k32	D	ASP966, GLY918, HIS746, SER965
326	1k4l	A	ASP41, ASP99, CYS66, GLU174, HIS136, TYR94
327	1k4t	A	ARG488, ARG590, HIS632, LYS532
328	1k82	D	ARG258, GLU2, LYS56, PRO1
329	1kae	A	GLU326, HIS327
330	1kas	A	CYS163, GLU314, HIS303, HIS340, LYS335, PHE400
331	1kcz	A	HIS194, LYS331
332	1kdg	A	ASN732, HIS689
333	1kfu	L	ASN286, CYS105, GLN99, HIS262, TRP288
334	1kim	A	ARG163, ARG220, ARG222, GLU225, GLU83, LYS62
335	1kl7	A	LYS124
336	1knp	A	ARG290, HIS351, LEU386
337	1kny	A	GLU145, LYS149
338	1kp2	A	ARG106, ASP135
339	1kqc	C	LYS14, LYS74
340	1kra	C	ARG336, ASP221, HIS219, HIS320
341	1ksj	A	GLN70
342	1kws	A	GLU281
343	1kyq	A	ASP141
344	1kyw	F	HIS269
345	1kzh	A	ARG146, ASP206, GLY82, LYS203, THR204
346	1l0o	B	ARG105, GLU46
347	1l1d	B	ARG493, ASP484, CYS440, CYS495, HIS480
348	1l1l	A	CYS119, CYS408, CYS419, GLU410
349	1l1r	A	ARG63, GLU100
350	1l6p	A	ASP68, CYS103, CYS109, PHE70, TYR42, TYR71
351	1l7d	D	ARG1327, ASP1335, GLN1332, SER1338
352	1l7n	B	ASP511, ASP513, ASP671, GLY600, LYS644, PHE512
353	1l7q	A	ALA117, ASP259, HIS287, TRP166, TYR118, TYR44
354	1l8t	A	ASP190, LYS44
355	1l9x	D	CYS110, HIS220
356	1lam	A	ARG336, LYS262
357	1lba	A	LYS128, TYR46
358	1lbu	A	ASP194, HIS192, HIS195, TYR189

359	1lci	A	ARG218, HIS245, LYS529, THR343
360	1lio	A	ARG136, ASP318
361	1lml	A	GLU265
362	1lnh	A	ASN713
363	1ltq	A	ARG126, ASP165, ASP35, LYS15
364	1lvh	A	LYS145, SER114
365	1lws	A	LYS301, LYS403
366	1lz1	A	ASP53, GLU35
367	1m21	B	LYS123, SER202, SER226
368	1m54	B	LYS119
369	1mas	A	ASN168, ASP10, HIS241
370	1mbb	A	ARG159, GLU325, SER229
371	1mdr	A	ASP270, GLU317, HIS297, LYS166
372	1mfp	B	LYS1163, TYR1156
373	1mhy	D	CYS151, THR213
374	1mj9	A	GLU338, SER304
375	1mka	A	ASP84, CYS80, GLY79, HIS70, VAL76
376	1mlv	C	TYR287
377	1mok	D	CYS82, CYS87, HIS137, LEU78, PHE501
378	1moq	A	GLU481, GLU488, LYS485, LYS603
379	1mpx	A	ASP307, HIS340, SER174, TYR175, TYR82
380	1mqw	A	ARG64, GLU142
381	1mrq	A	ASP50, HIS117, LYS84, TYR55
382	1mt1	L	GLU109
383	1mt5	A	GLY239, GLY240, ILE238, LYS142, SER217, SER218, SER241
384	1mud	A	ASN138, GLU37
385	1mug	A	ASN18
386	1mvn	A	HIS90, SER175
387	1myr	A	ARG95, ASN328, GLN187, GLU409, SER190, TYR330
388	1n29	A	ASP91, GLY29, HIS47
389	1n2c	F	ASP129, LYS10
390	1n2t	A	ALA199, ALA223, ARG360, ASP197, GLN200, HIS114
391	1n5w	B	ARG387, CYS388, GLN240
392	1nbf	B	ASN218, ASP481, CYS223, HIS464
393	1ndh	A	TYR65
394	1ndi	B	HIS343, SER554
395	1nf9	A	ASP38
396	1nhx	A	ARG405, HIS264
397	1nid	A	ASP98, HIS255
398	1nir	B	HIS327, HIS369
399	1nkk	A	ARG165, HIS157, HIS63, SER132
400	1nln	A	CYS122, GLN115, GLU71, HIS54
401	1nlu	A	ASP170, ASP84, GLU80, SER287
402	1nml	A	GLU114
403	1nmw	A	CYS113, HIS157, HIS59
404	1nn4	A	ARG137, ASP9, CYS66, HIS10
405	1nsf	A	LYS549, LYS631, LYS708
406	1nsj	A	ASP126, CYS7
407	1nsp	A	ASN119, LYS16
408	1nvm	A	HIS21, TYR291
409	1nvt	A	ASP102
410	1nw9	B	ARG178, CYS287, GLY238, HIS237

411	1nww	A	ARG99, ASN55, ASP101, ASP132, TYR53
412	1o04	C	GLU268, GLU399, LYS192, SER302
413	1o98	A	ARG261, ASP154, SER62
414	1o9i	A	GLU178
415	1oac	A	ASP383
416	1oas	B	LYS41, SER272
417	1oba	A	ASP10, ASP182, ASP92, GLU94
418	1odt	C	ALA181, ASP269, GLN182, HIS298
419	1oe8	A	TYR10
420	1ofg	B	LYS129, TYR217
421	1og1	A	ARG184, GLU159, GLU189, SER147
422	1ogo	X	ASP376, ASP395
423	1oh9	A	GLY11, GLY45, LYS217, LYS8
424	1ohh	C	GLN208, LYS175, LYS209
425	1ohv	D	ASP298, LYS329, PHE189
426	1oj4	A	ASP141, LYS10
427	1ok4	J	ASP24, LYS177, TYR146
428	1okg	A	ASP61, HIS75, SER255
429	1opm	A	GLN170, HIS108, HIS242
430	1or8	A	GLU144, GLU153
431	1ord	A	ASP316, HIS223, LYS355
432	1oro	A	LYS103
433	1otg	A	ARG41, PHE35, PRO2
434	1oxa	A	GLU360, SER246
435	1oyg	A	ASP247, ASP86, GLU342
436	1ozh	D	MET394, MET422
437	1p1x	B	ASP1102, LYS1167, LYS1201
438	1p3d	A	LYS129
439	1p4n	A	ARG211, ASP108, GLU319, LYS36
440	1p7m	A	GLU38, TRP46, TYR16
441	1pa9	A	ARG247, ASP194, CYS241, THR248
442	1pad	A	ASN175, CYS25, HIS159
443	1pbg	A	GLU160, GLU375
444	1pd2	2	ARG14, TRP104, TYR8
445	1peg	A	TYR178, TYR283
446	1pfk	A	ARG171, ARG72, ASP127, GLY11, THR125
447	1pgs	A	ASP60, GLU206
448	1pii	A	ASN184, GLU163, GLU53, LYS114, LYS55, SER215
449	1pix	B	ALA457, GLY194, ILE417, VAL151
450	1pj5	A	ASP552, HIS225, TYR259
451	1pja	A	ASP228, GLN112, HIS283, LEU45, SER111
452	1pjh	B	ALA70, GLU158, LEU126
453	1pjq	B	ASP248, LYS270, MET382
454	1pma	N	GLY80, LYS66, SER167, SER35
455	1pmi	A	ARG304, GLN111, GLU294
456	1pnl	B	ALA69, ASN241, SER1
457	1pnt	A	ARG18, ASP129, CYS12, CYS17
458	1pow	B	ARG264, GLU483, PHE479
459	1pp4	A	ASN74, ASP192, GLY42, HIS195, SER9
460	1psd	A	GLU269, HIS292
461	1pud	A	ASP102
462	1pvd	B	ASP28, GLU477, HIS114, HIS115

463	1pvi	B	LYS70
464	1pwv	B	GLU687, TYR728
465	1pyl	B	ARG67, GLU56, HIS86
466	1pz3	A	GLU175
467	1q18	B	ASP100
468	1q3n	A	HIS202
469	1q3q	A	ASP393, ASP64, THR97, THR98
470	1q6l	B	ARG139, GLU112, HIS136, ILE37, LYS64, THR36
471	1q6x	B	HIS334, PRO108, SER550, TYR95
472	1q91	A	ASP41, ASP43
473	1qam	A	ASN101, GLU59, GLY38
474	1qaz	A	ARG239, ASN191, TYR246
475	1qb4	A	ARG396, ARG581, ARG713
476	1qba	A	ASP539, GLU540
477	1qcn	A	ARG237, GLN240, GLU199, GLU364, HIS133, LYS253
478	1qd1	A	HIS82
479	1qe3	A	GLU310, HIS399, SER189
480	1qf6	A	ARG363
481	1qfl	A	CYS378, HIS348
482	1qfm	A	ASP641, HIS680, SER554
483	1qfn	A	ARG8, GLY10, LYS18, TYR13, TYR72
484	1qgn	H	ASP236, LYS261, TYR163
485	1qgx	A	ASP294, ASP49, THR147
486	1qh9	A	ARG41, ASP10, ASP180, SER118
487	1qhf	B	ARG59, GLU86, HIS181, HIS8
488	1qhg	A	ARG610, GLU224, LYS37
489	1qho	A	ASP228, ASP329, GLU256, HIS132
490	1qi9	B	HIS411, HIS418, HIS486, LYS341
491	1qje	A	PHE211
492	1qk2	B	ARG174, ASP175, ASP221, TYR169
493	1qmh	B	HIS309
494	1qol	H	ALA51, ASP163, HIS148
495	1qrg	A	ASN202, GLN75, GLU62, GLU84
496	1qtn	A	ARG258, CYS360, GLY350, HIS317
497	1qv0	A	HIS175, HIS22, TRP92, TYR138, TYR190
498	1qwn	A	ASP204, ASP341
499	1qx3	A	CYS163, GLY122, HIS121
500	1qz9	A	ASP201, LYS227, PHE129
501	1r16	B	GLU179, GLU98
502	1r1j	A	ARG717, ASP650, GLU584, HIS711
503	1r30	B	ARG260, CYS53, CYS57, CYS60
504	1r44	B	ARG71, GLU181
505	1r4f	A	ALA260, ASN186, ASP10, TRP83
506	1r4z	A	ASP133, HIS156, ILE12, MET78, SER77
507	1r6w	A	ARG133, LYS235
508	1r76	A	ARG307, ASN172
509	1ra0	A	GLN156, GLU217
510	1ra2	A	ASP27, ILE5, ILE94, LEU28, LEU54, MET20, PHE31
511	1rba	A	ASN192, HIS287, HIS321, LYS166, LYS191
512	1rbl	A	ASP203, HIS294, HIS327, LYS175, LYS177, LYS201
513	1rbn	A	HIS119, HIS12, LYS41, PHE120
514	1rgq	B	ASP84, GLY140, HIS60, SER142

515	1rhc	A	GLU108, HIS39, TRP43
516	1rk2	D	ALA252, ALA253, ASP255, GLY254
517	1ro7	A	ARG129, HIS188, TYR156, TYR162
518	1roz	A	GLU137, HIS288, LYS329
519	1rpt	A	ARG11, ARG15, ARG79, ASP258, HIS12, HIS257
520	1rpx	B	ASP185, ASP43, HIS41, HIS74
521	1rtf	B	ASP102, GLY193, HIS57, SER195
522	1rtu	A	GLU62, HIS101, HIS41
523	1rvv	J	HIS88
524	1s20	A	HIS44
525	1s2k	A	GLN53, GLU136
526	1s3i	A	ASP142, HIS106
527	1s76	D	LYS631
528	1s95	A	ARG275, ARG400, ASN303, ASP274, HIS304, HIS427
529	1s9c	C	ASP193, GLY216, HIS198, ILE213
530	1sca	A	ASP32, HIS64, SER221
531	1ses	B	ARG256, ARG271, ASP265, GLU258, SER261
532	1sll	A	ASP318, GLU595, TYR713
533	1slm	A	GLU202
534	1sme	A	ASP214, ASP34, SER37, THR217
535	1smn	B	ARG87, ASN119, HIS89
536	1snn	B	ASN106, ASP30, CYS55, GLU185, SER147, TYR95
537	1snz	B	GLU307, HIS176
538	1ssx	A	ASP102, GLY193, HIS57, SER195, SER214
539	1stc	E	ASP166, LYS168, THR201
540	1std	A	ASP31, HIS110, HIS85, TYR30, TYR50
541	1su4	A	ASP351
542	1szj	G	CYS149, HIS176
543	1t0u	A	ARG223, GLU80, HIS8
544	1t7d	A	LYS145, SER88, SER90
545	1tde	A	ASP139, CYS135, CYS138
546	1tdj	A	LYS62, SER315
547	1teh	B	HIS47, THR48
548	1thg	A	ALA132, ALA218, GLU354, HIS463, SER217
549	1tht	A	HIS241, SER114
550	1tlp	E	GLU143, HIS231
551	1tml	A	ASP117, ASP265
552	1tmo	A	SER149
553	1tox	A	GLU148
554	1trk	A	HIS263, HIS30
555	1tyf	M	ASP171, GLY68, HIS122, MET98, SER97
556	1tys	A	ARG166, ASP169, GLU58, SER146, TYR94
557	1tz3	A	ALA250, ASP252, GLY249, GLY251
558	1u3f	B	ARG115
559	1u5u	B	ASN137, HIS67, THR66, TYR193
560	1u7u	A	ASP210
561	1u8v	C	HIS292
562	1uae	A	ARG397, ASN23, ASP305, CYS115
563	1uam	A	ASP169
564	1uaq	B	CYS91, GLU64, SER89
565	1uas	A	ASP130, ASP185
566	1uch	A	ASP184, CYS95, GLN89, HIS169

567	1uf7	A	ALA171, GLU46, LYS126
568	1ujn	B	ARG224, ASN228, GLU220, HIS235, LYS131, LYS210
569	1uk7	A	ALA103, ASP224, HIS252
570	1un1	B	ASP87, GLU85, GLU89
571	1uok	A	ASP199, ASP329, GLU255
572	1uqr	J	ARG107, ARG18, ASN11, GLU98, HIS100, TYR23
573	1uqt	B	ASP361, HIS154
574	1uro	A	ASP86, TYR164
575	1ush	A	ARG375, ARG379, ARG410, ASN116, ASP120, HIS117
576	1v04	A	HIS115, HIS134
577	1v0e	C	ARG596, ARG647, GLU581
578	1v0y	A	ASP202, ASP473, HIS170, HIS448
579	1vid	A	GLU199, LYS144
580	1vie	A	GLN67, ILE68, LYS32, TYR69
581	1vnc	A	HIS404, LYS353
582	1vom	A	ASN233, GLU459, GLY182, GLY457
583	1vq1	A	ASN197, PHE100, PRO198
584	1w0h	A	GLU136, HIS293
585	1w27	B	PHE400
586	1w2n	A	ARG74
587	1wd8	A	ASP350, ASP473, CYS645, HIS471
588	1wgi	B	ASP117
589	1wnw	B	ASN136, GLY140
590	1x7d	A	ASP228, GLU56
591	1x9h	B	ARG135, GLU203, HIS219, LYS298
592	1x9y	B	ASN360, CYS243, GLN237, HIS340
593	1xa8	C	CYS56
594	1xff	A	ASN98, CYS1, GLY99
595	1xgm	A	GLU187
596	1xik	A	TYR122
597	1xqd	A	ASP393, SER286
598	1xqw	A	ALA105, ASP244, GLY37, HIS271, TYR106
599	1xrs	B	LYS144
600	1xs1	C	ALA124, ARG126, GLU138
601	1xtc	A	ARG7, GLU110, GLU112, SER61
602	1xvt	A	ASP169
603	1xyz	A	ASP756, GLU645, GLU754, HIS723
604	1y9m	A	ASP41, GLU241
605	1ybq	B	ASN285
606	1ybv	A	ASN138, LYS182, SER164, TYR178
607	1ycf	B	HIS25, TYR195
608	1ygh	A	GLU173
609	1yon	A	LYS176
610	1ysc	A	ASP338, HIS397, SER146
611	1yve	L	GLU496
612	1zio	A	ARG127, ARG160, ARG171, ASP162, ASP163, LYS13
613	1zm2	B	GLU553
614	1zoi	B	ALA122, ASP227, HIS256, SER97, THR98, TRP31
615	1zrz	A	ASN374, ASP369, LYS371
616	1zym	A	HIS189, THR168
617	206l	A	ASP20, GLU11
618	2a0n	A	ASP11, ASP130

619	2a86	B	ARG198, HIS44, HIS47, LYS160, MET40, SER196, SER197
620	2abk	A	ASP138, LYS120
621	2ace	A	GLU327, HIS440, SER200
622	2acu	A	ASP43, HIS48, LYS77
623	2acy	A	ARG23, ASN41
624	2ag0	A	GLN113, GLU50, HIS29
625	2alr	A	LYS79, TYR49
626	2amg	A	ASP193, ASP294, GLU219
627	2apr	A	ASP218, ASP35, SER38, THR221
628	2ayh	A	ASP107, GLU105, GLU109
629	2bhg	A	ALA163, ASP84, HIS46, SER182
630	2bif	B	ALA256, ARG255, ARG305, ASN262, GLU325, HIS390
631	2bkr	A	ASP119, CYS163, HIS102, TRP103, TRP26
632	2bmi	A	ASN176, ASP86
633	2bsx	A	ASP206
634	2bx4	A	CYS145, HIS41
635	2cnd	A	CYS242, PHE270, THR65
636	2cpu	A	ASN300, ASP197, GLU233
637	2dbt	C	ASN194, GLU147, GLU156
638	2dhn	A	GLU22, LYS100
639	2dln	A	ARG255, GLU15, GLY276, SER150, TYR216
640	2dor	B	CYS130, LYS43
641	2dw7	O	ASN55, ASP292, GLU341, HIS322, LYS182, LYS184
642	2ebn	A	ASP130, GLU132
643	2eng	A	ASP10, ASP121
644	2eql	A	ASP53, GLU35
645	2esd	D	ALA250, ASN154, CYS284
646	2f61	A	CYS342, GLU235, GLU340
647	2f9z	C	CYS27, HIS44, THR21
648	2fmn	B	ASP120, GLU28
649	2fok	A	LYS469
650	2hdh	A	ASN208, GLU170, HIS158, SER137
651	2hgs	A	ARG125, ARG450, GLY369, SER151
652	2hi7	B	ARG48, CYS104, CYS41, CYS44
653	2hsa	A	HIS185, HIS188, TYR190
654	2isd	A	GLU341, HIS311, HIS356
655	2jxr	A	ASP215, ASP32, THR218, THR33
656	2lip	A	ASP264, GLN88, HIS286, LEU17, SER87
657	2nac	B	ARG284, ASN146, GLN313, HIS332
658	2nlr	A	GLU120
659	2nmt	A	ASN169, LEU171, LEU455, PHE170
660	2oat	A	ASP263, LYS292, PHE177
661	2ocp	C	ARG142, GLU70
662	2pda	A	ARG114, ASN996, GLU64, THR31
663	2pec	A	ARG218, ASP131
664	2pgd	A	ASN187, GLU190, GLY130, LYS183
665	2phk	A	ASP149, LYS151
666	2pia	A	ASN44, CYS199, GLU223, SER58
667	2plc	A	ARG84, ASP278, ASP46, HIS45, HIS93
668	2pth	A	ASN10, ASP93, HIS20
669	2rnf	B	HIS116, HIS12, LYS40

670	2sqc	B	ARG127, ASP374, ASP377, CYS376, GLN262, GLU45, GLU93, HIS451, PHE365, PHE601, PHE605, TRP169, TRP312, TRP489, TYR495
671	2tdt	A	ASP141, GLU169, GLY166
672	2thi	A	CYS113, GLU241
673	2tpl	A	ARG381, ASP214, PHE123, TYR71
674	2tps	B	ARG59, LYS159, SER130
675	2xis	A	ASP57, GLU181, HIS54, LYS183
676	2ypn	A	ARG131, ARG132, ARG149, ARG155, ASP84, CYS242
677	3cla	A	ARG18, ASP199, HIS195
678	3eca	D	ASP90, LYS162, THR12, THR89, TYR25
679	3mdd	B	GLU376
680	3nos	B	ARG187, CYS184, GLU361, TRP356
681	3pca	N	ARG457, TYR447
682	3r1r	A	ASN437, CYS225, CYS439, CYS462, GLU441
683	3s8r	B	ALA170, GLU624, VAL239, HIS192, ASN413
684	4kbp	D	HIS202, HIS295, HIS296
685	4mdh	B	ASP158, HIS186
686	7nn9	A	ARG220, ARG371, ASP151, GLU277, TYR412A
687	7odc	A	GLU274, HIS197, LYS69
688	8pch	A	CYS25, GLN19, HIS159

Average (Standard deviation) number of catalytic residues = 3.06 (1.7)

EF_POOL_PW dataset (286 proteins)

S.No.	PDB	Chain	Catalytic site residues
1	12as	A	ASP46, ARG100, GLN116
2	13pk	A	ARG39, LYS219, GLY376, GLY399
3	1a26	A	TYR907, GLU988
4	1a41	A	TYR274, ARG223, HIS265, LYS167
5	1a4i	A	LYS56
6	1a4s	A	ASN166, GLU263, CYS297
7	1a4y	B	HIS13, LYS40, HIS114
8	1a6d	A	THR97, ASP390, THR96, ASP63
9	1a79	A	LYS156, TYR115, HIS125
10	1aa6	A	ARG333, HIS141, LYS44
11	1ab4	A	TYR122, ARG32, HIS78
12	1adn	A	CYS69
13	1ae7	A	GLY30, HIS48, ASP99
14	1af7	A	ARG98, ASP154
15	1afr	A	THR199, ASP228, TRP62, HIS146
16	1afw	A	CYS125, HIS375, CYS403, GLY405
17	1ah7	A	ASP55
18	1ak0	A	ARG48
19	1akm	A	ARG106, HIS133, GLN136, ASP231, CYS273, ARG319
20	1ako	A	HIS259, ASP229, ASN7, ASP151, ASN153
21	1alk	A	SER102, ARG166
22	1am2	A	HIS75, ASN198, ASN74, SER1, HIS197, THR72
23	1aop	A	ARG83, ARG153, LYS215, LYS217, CYS483
24	1apx	A	ARG38, HIS42, ASN71
25	1apy	B	THR183, THR201, THR234, GLY235
26	1aq2	A	HIS232, LYS254, ARG333
27	1aqf	A	ARG72, ARG119, LYS269, THR327, SER361, GLU363
28	1aui	A	ASP121, HIS151
29	1auk	A	LYS302, LYS123, HIS229, HIS125, ASP281, SER150
30	1aw8	B	TYR58
31	1b3r	A	ASP130, LYS185, ASP189, ASN190, CYS194
32	1b5q	A	GLU62
33	1b65	A	ASN218, GLY289, TYR146, SER288, SER250
34	1b6b	A	SER97, LEU111, HIS122, LEU124, TYR168
35	1b73	A	ASP7, SER8, CYS70, CYS178
36	1b93	A	HIS19, GLY66, ASP71, ASP91, HIS98, ASP101
37	1bd3	A	THR141, ARG137, ASP235
38	1bfd	A	GLU28, HIS281, HIS70
39	1bg0	A	ARG126, GLU225, ARG229, ARG280, ARG309
40	1bg6	A	ASP297, HIS202
41	1bml	A	HIS603, ASP646
42	1bmt	A	SER810, ASP757, HIS759
43	1bo1	A	ASP278, LYS150
44	1bob	A	GLU255
45	1bol	A	HIS46, GLU105, HIS109
46	1bou	B	HIS195
47	1brm	A	CYS135, GLN162, HIS274
48	1brw	A	HIS82, ARG168, SER183, LYS187
49	1bs4	A	GLY45, GLN50, LEU91, GLU133

50	1btl	A	SER70, LYS73, SER130, GLU166
51	1bwd	A	ASP108, ARG127, ASP179, HIS227, ASP229, HIS331, CYS332
52	1bwp	A	SER47, GLY74, ASN104, ASP192, HIS195
53	1bzy	A	GLU133, ASP134, ASP137, LYS165, ARG169
54	1c3j	A	GLU22, ASP100
55	1c4x	A	HIS263, SER110, ASP235
56	1c4z	A	GLU550, ARG506, CYS820, HIS818, GLU539, ASP607
57	1cb8	A	HIS225, TYR234, ARG288
58	1cd5	A	ASP72, ASP141, HIS143, GLU148
59	1cdd	A	ASN106, HIS108, SER135, ASP144
60	1cef	A	ASN161, SER62, LYS65, TYR159, HIS298
61	1cf2	O	CYS140, HIS219
62	1cfr	A	LYS190
63	1cg2	A	GLU175, GLU200, HIS112, ASP141, HIS385, GLU176
64	1chd	A	SER164, THR165, HIS190, MET283, ASP286
65	1chk	A	GLU22, ASP40
66	1chm	A	HIS232, GLU262, GLU358
67	1cjy	A	SER228, GLY198, GLY197, ASP549
68	1coy	A	GLU361, HIS447, ASN485
69	1cqq	A	HIS40, GLU71, GLY145, CYS147
70	1ctt	A	GLU104
71	1cul	B	ARG1029
72	1d0s	A	GLU317
73	1d1q	A	ALA13, ARG19, SER20, ASP132
74	1d4a	A	GLY149, TYR155, HIS161
75	1d4c	A	HIS364, ARG401, HIS503, ARG544
76	1d5r	A	ASP92, ARG130, CYS124
77	1daa	A	LYS145, GLU177, LEU201
78	1dae	A	THR11, LYS15, LYS37, SER41
79	1db3	A	THR132, GLU134, TYR156, LYS160
80	1dbt	A	ASP60, LYS62
81	1dci	A	ASP204, GLU196
82	1dco	C	HIS62, HIS63, HIS80, ASP89
83	1dgs	A	LYS116, ASP118, ARG196, LYS312
84	1di1	A	TYR92, PHE112, TRP333, PHE178
85	1dii	A	TYR95, GLU380, GLU427, HIS436, ARG474
86	1diz	A	TYR222, TRP272, ASP238
87	1dj0	A	ASP60
88	1dki	A	SER47, HIS195
89	1dl2	A	GLU132, ARG136, ASP275, GLU435
90	1dl5	A	SER59
91	1dli	A	THR118, GLU145, LYS204, ASN208, CYS260, ASP264
92	1dnk	A	GLU78, HIS134, ASP212, HIS252
93	1dnp	A	TRP306, TRP359, TRP382
94	1do8	A	TYR112, LYS183, ASP278
95	1dos	A	ASP109, GLU182, ASN286
96	1dqs	A	HIS275
97	1dyu	A	ASP383
98	1dzr	A	HIS63, ASP170
99	1e0c	A	HIS234, GLN231, HIS233, ARG235, THR232
100	1e2a	A	HIS78, GLN80, ASP81, HIS82

101	1ebf	A	ASP219, LYS223
102	1ecf	A	GLY102, CYS1, ASN101
103	1eej	A	CYS98, TYR100, CYS101, ARG125
104	1ef8	A	HIS66, GLY110, TYR140
105	1eg7	A	LYS1074
106	1el9	A	HIS269, LYS348, LYS265, CYS315, THR48
107	1et0	A	LYS159, THR38, GLU193
108	1eu1	A	TYR114, TRP116
109	1eug	A	ASP64, HIS187
110	1eyi	A	ASP68, ASP74, GLU98
111	1f6d	A	GLU131, ASP95, GLU117, HIS213
112	1f75	A	ARG42, ARG33, ARG197, ARG203
113	1f7l	A	LYS62, HIS105
114	1f7u	A	LYS156, HIS162, HIS159
115	1fft	F	HIS419, LYS362, ASP135, THR211, THR359, SER315, SER299, ASN142, TYR61, THR201, SER145, MET79, ASN124, PHE420, PHE103, GLU286, THR149, HIS284, HIS421, THR204, ASP75, ARG71, TYR288
116	1fgh	A	ASP100, HIS101, HIS147, ASP165, HIS167, GLU262, SER642
117	1foh	A	ASP54, ARG281, TYR289
118	1fr2	B	HIS102, HIS103, GLU100, ARG5
119	1fro	A	GLU172
120	1fua	A	GLU73
121	1fui	A	GLU337, ASP361
122	1g0d	A	CYS272, ASP355, HIS332, TYR515
123	1g24	A	GLU214
124	1g72	A	ASP297
125	1g8f	A	ARG290
126	1ga8	A	GLN189, ASP130, ASN153
127	1gim	A	ASP13, HIS41, GLN224
128	1gog	A	CYS228, TYR272, TRP290, TYR495
129	1gpj	A	SER48, HIS84
130	1gpm	A	GLY59, CYS86, TYR87, HIS181, GLU183, ASP239
131	1gpr	A	THR66, HIS68, HIS83, GLY85
132	1gq8	A	ASP136, GLN113, GLN135, ASP157
133	1gsa	A	ARG225, LYS160, ARG210
134	1gt7	A	GLU117, GLU171
135	1gtp	A	HIS112, HIS179
136	1h3i	A	TYR335, HIS293
137	1h4g	B	GLU184, GLU94
138	1h54	A	GLU487
139	1hfe	M	LYS237, CYS178
140	1hfs	A	GLU202, MET219
141	1hr6	B	GLU73
142	1hrk	A	GLU343, HIS263, HIS341
143	1huk	A	ASP117
144	1hxq	A	CYS160, HIS164, HIS166, GLN168
145	1i19	A	GLU311, GLU475, ARG477
146	1i6p	A	ASP44, ARG46
147	1i78	A	HIS212, ASP85, ASP83, ALA99, ASP210
148	1i7d	A	GLU7, LYS8, PHE328, ARG330

149	1i8d	A	HIS102, CYS48, MET64, PHE2, SER41
150	1i8t	B	ASP348, ARG278, GLU298, ARG170
151	1inp	A	LYS37, THR158
152	1iph	A	HIS128, SER167, ASN201
153	1itx	A	GLU204, ASP200
154	1ivh	A	GLU254
155	1j09	A	LYS246
156	1jch	A	ASP510, HIS513, ARG545, GLU517
157	1jdw	A	ASP254, HIS303, CYS407
158	1jfl	A	CYS82, CYS194
159	1jh6	A	HIS42, SER121, MET117, HIS119, TYR124
160	1jhf	A	MET118, GLU152, LYS156, SER119, ASP127
161	1jm6	A	HIS1239, GLU1243
162	1jms	A	ASP434
163	1k30	A	HIS139, ASP144
164	1k32	A	SER965, HIS746, ASP966, GLY918
165	1k4t	A	HIS632, ARG488, ARG590
166	1k82	A	GLU2, LYS56, PRO1, ARG258
167	1kas	A	CYS163, HIS303, HIS340, PHE400
168	1kfu	L	GLN99, CYS105, HIS262, ASN286
169	1kl7	A	LYS124
170	1kny	A	GLU145, LYS149
171	1kr3	A	ASP103, ASN193
172	1kra	C	HIS219, ASP221, HIS320, ARG336
173	1kyq	A	ASP141
174	1kzh	A	THR204, ASP206, GLY82, LYS203, ARG146
175	1l0o	A	GLU46, ARG105
176	1l1d	A	HIS480, ARG493, CYS495, CYS440, ASP484
177	1lam	A	LYS262, ARG336, ASP255
178	1lba	A	LYS128
179	1lbu	A	HIS192
180	1lcb	A	GLU60, CYS198, SER219, ASP221, ASP257, HIS259
181	1lci	A	HIS245, ARG218, LYS529, THR343
182	1ldm	A	HIS193, ASP166, ARG169
183	1lxa	A	HIS125
184	1m6k	A	SER67
185	1mas	A	ASN168, HIS241
186	1mbb	A	ARG159, SER229, GLU325
187	1mek	A	CYS36, GLY37, HIS38, CYS39
188	1mhy	D	CYS151, THR213
189	1mla	A	SER92, HIS201, GLN250
190	1moq	A	GLU481, LYS485, GLU488, HIS504, LYS603
191	1mpx	A	TYR175, HIS340, SER174, TYR82, ASP307
192	1mpy	A	HIS199, HIS246, TYR255
193	1muc	A	LYS169, LYS167, GLU327
194	1mug	A	ASN18
195	1nba	A	ASP51, LYS144, ALA172, THR173, CYS177
196	1nf9	A	ASP38
197	1nid	A	ASP98, HIS255
198	1nsp	A	ASN119, LYS16
199	1nvm	A	TYR291, HIS21
200	1nww	A	TYR53, ARG99, ASP101, ASN55, ASP132

201	1nzy	A	PHE64, HIS90, GLY114, TRP137, ASP145
202	1o04	A	SER302, LYS192, GLU268, GLU399
203	1o8a	A	GLU384, HIS353, HIS513, ALA354, TYR523
204	1o98	A	SER62, ASP154, ARG261
205	1oe8	A	TYR10
206	1ofg	A	LYS129, TYR217
207	1otg	A	PRO2, PHE35, ARG41
208	1p3d	A	LYS129
209	1pfk	A	GLY11, ARG72, THR125, ASP127, ARG171
210	1pjb	A	LYS74, HIS95, GLU117, ASP269
211	1pmi	A	GLN111, ARG304, GLU294
212	1pnf	A	ASP60, GLU206
213	1pnl	B	SER1, ALA69, ASN241
214	1ps1	A	PHE77, HIS309
215	1pud	A	ASP102
216	1pvd	A	ASP28, HIS114, HIS115, GLU477
217	1pym	A	GLY47, LEU48, ASP58, LYS120
218	1q23	A	ASP197, HIS193, ARG18
219	1qae	A	ARG87, HIS89, ASN119
220	1qba	A	GLU540, ASP539
221	1qf6	A	ARG363
222	1qfe	A	GLU86, HIS143, LYS170
223	1qfm	A	ASP641, SER554, HIS680
224	1qh5	A	ASP58
225	1qho	A	ASP228, ASP329, GLU256, HIS132
226	1qi9	A	HIS411, HIS418, HIS486, LYS341
227	1qmh	A	HIS309
228	1qq5	A	ASP8, THR12, ARG39, ASN115, LYS147, SER171, ASN173, PHE175, ASP176
229	1qtn	A	GLY350, HIS317, CYS360, ARG258
230	1qum	A	GLU261
231	1r51	A	ARG176, GLN228
232	1ra2	A	ILE5, MET20, ASP27, LEU28, PHE31, LEU54, ILE94
233	1rbl	A	LYS175, LYS177, LYS201, ASP203, HIS294, HIS327
234	1req	A	TYR89, HIS244, LYS604, ASP608, HIS610
235	1rpt	A	ARG11, HIS12, ARG15, ARG79, HIS257, ASP258
236	1rtu	A	GLU62, HIS101, HIS41
237	1sox	A	ARG138, TYR322
238	1su4	A	ASP351
239	1tml	A	ASP117, ASP265
240	1tyf	A	GLY68, SER97, MET98, HIS122, ASP171
241	1uae	A	ASN23, CYS115, ASP305, ARG397
242	1uag	A	LYS115, ASN138, HIS183
243	1uaq	A	GLU64, CYS91, SER89
244	1ula	A	HIS86, GLU89, ASN243
245	1uok	A	ASP199, GLU255, ASP329
246	1ush	A	ARG375, ASP120, ARG410, HIS117, ARG379, ASN116
247	1vao	A	TYR108, ASP170, HIS422, TYR503, ARG504
248	1vnc	A	LYS353, HIS404
249	1xje	A	CYS322, CYS333, ASN320, GLU324, LYS40
250	1xva	A	GLU15
251	1ytw	A	GLU290, ASP356, HIS402, CYS403, ARG409, THR410

252	1zio	A	LYS13, ARG127, ARG160, ASP162, ASP163, ARG171
253	1zym	A	HIS189, THR168
254	2abk	A	ASP138, LYS120
255	2acy	A	ARG23, ASN41
256	2adm	A	ASN105, PRO106, TYR108
257	2alr	A	TYR49, LYS79
258	2apr	A	THR221, SER38, ASP35, ASP218
259	2bbk	L	TYR119, ASP76, THR122, TRP108, ASP32
260	2cpo	A	HIS105, GLU183
261	2hdh	A	SER137, HIS158, GLU170, ASN208
262	2hgs	A	ARG125, SER151, GLY369, ARG450
263	2jcw	A	HIS63, ARG143
264	2jqx	A	ASP270, GLU272, ARG338, ASP631
265	2pda	A	GLU64
266	2pfl	A	TRP333, CYS418, CYS419, GLY734
267	2pgd	A	ASN187, LYS183, GLY130, GLU190
268	2phk	A	ASP149, LYS151
269	2pia	A	CYS199, ASN44, SER58, GLU223
270	2plc	A	HIS45, ASP46, ARG84, HIS93, ASP278
271	2thi	A	CYS113, GLU241
272	2vya	A	LYS142, SER218, ILE238, SER241, GLY240, GLY239, SER217
273	2w0w	A	ARG18
274	3csm	A	ARG16, ARG157, LYS168, GLU246
275	3eca	A	THR12, TYR25, THR89, ASP90, LYS162
276	3kyh	A	ARG393, GLU433, LYS456, ARG458
277	3mdd	A	GLU376
278	3pca	M	TYR447, ARG457
279	3pep	A	ASP32, SER35, TYR75, ASP215
280	4kbp	A	HIS202, HIS295, HIS296
281	5cox	A	GLN203, HIS207, TYR385
282	5enl	A	GLU168, GLU211, LYS345, HIS373
283	5fit	A	GLN83, HIS94, HIS96
284	7odc	A	GLU274, LYS69, HIS197
285	8tln	E	GLU143, HIS231
286	9pap	A	ASN175, GLN19, HIS159

Average (Standard deviation) number of catalytic residues = 3.21 (1.93)

CSAMAC dataset (884 proteins)

S.No.	PDB	Chain	Catalytic site residues
1	12as	A	ASP46, ARG100, GLN116
2	13pk	A	ARG39, LYS219, GLY376, GLY399
3	1a0i	A	LYS34, LYS238, LYS240
4	1a26	A	TYR907, GLU988
5	1a4s	A	ASN166, GLU263, CYS297
6	1a7u	A	ASP228, HIS257, SER98, MET99, PHE32
7	1adn	A	CYS38
8	1afr	A	TRP62, HIS146, THR199, ASP228
9	1afw	A	CYS125, HIS375, CYS403, GLY405
10	1ah7	A	ASP55
11	1ako	A	ASN7, ASP151, ASN153, ASP229, HIS259
12	1al6	A	SER244, HIS274, HIS320, ASP375
13	1alk	A	SER102, ARG166
14	1amo	A	SER457, CYS630, ASP675
15	1aq2	A	HIS232, LYS254, ARG333
16	1auk	A	ASP281, SER150, LYS123, LYS302, HIS125, HIS229, ARG73
17	1b3r	A	HIS54, ASP130, LYS185, ASP189, ASN190, CYS194, HIS300, SER360
18	1b57	A	ASP109, GLU182, ASN286
19	1b6b	A	SER97, LEU111, HIS122, LEU124, TYR168
20	1b6t	A	ARG91, LYS42, HIS18, SER129
21	1ba3	A	THR343, LYS443, LYS529
22	1bc2	A	ASP90, ASN180
23	1bg0	A	ARG126, GLU225, ARG229, CYS271, THR273, ARG280, ARG309
24	1bmt	A	HIS759, ASP757, SER810
25	1bob	A	GLU255
26	1brw	A	HIS82, ARG168, SER183, LYS187
27	1bs1	A	LYS15, LYS37, SER41
28	1bsz	A	GLY45, GLN50, LEU91, GLU133
29	1bt3	A	PHE261
30	1btl	A	SER70, LYS73, SER130, GLU166, ALA237
31	1bwp	A	SER47, GLY74, ASN104, ASP192, HIS195
32	1bxk	A	TYR160, LYS164, THR134, ASP135, GLU136
33	1bzy	A	GLU133, ASP134, ASP137
34	1c0p	A	SER1335, ASN1054, GLN1339
35	1c9u	A	HIS144, ASP163, ARG228
36	1ca2	A	HIS64, GLU106, THR199
37	1cdg	A	ARG227, ASP229, GLU257, HIS327, ASP328
38	1cg6	A	ASP220
39	1chm	A	HIS232, GLU262, GLU358
40	1cju	B	ARG1029
41	1ct9	A	ALA1, GLY75, LEU50, ASN74, THR322, ALA325
42	1ctt	A	GLU104
43	1d0s	A	GLU317
44	1d4a	A	GLY149, TYR155, HIS161
45	1d6m	A	GLU7, LYS8, TYR328, ARG330
46	1d8c	A	ASP270, GLU272, ARG338, ASP631
47	1daa	A	TYR31, LYS145, GLU177, LEU201
48	1dci	A	ASP176, GLU196, ASP204
49	1dd8	A	CYS163, HIS298, LYS328, HIS333, PHE390, PHE392

50	1dea	A	ASP72, ASP141, HIS143, GLU148
51	1dhp	A	LYS161, TYR133, ILE203
52	1di1	A	TYR92, PHE112, PHE178, LYS206, TRP333
53	1djx	A	HIS311, ASN312, HIS356, GLU390
54	1dl2	A	GLU132, ARG136, ASP275
55	1dli	A	THR118, GLU145, LYS204, ASN208, CYS260, ASP264
56	1dnk	A	TYR76, GLU78, HIS134, ASP212, HIS252
57	1dnp	A	GLU274, TRP277, ASN341
58	1do8	A	TYR112, ARG165, LYS183, ASP278, ASN421
59	1doc	A	HIS72, TYR201, PRO293, LYS297, TYR385
60	1dqs	A	LYS152, LYS250, GLU260, ARG264, ASN268, HIS275
61	1dty	A	ASP245, TYR17, TYR144, LYS274
62	1e4e	A	HIS99, VAL19, GLY311, ARG290, TYR315, LYS22, GLU250, HIS244
63	1e6e	A	HIS55, ASP159, ILE376, THR377
64	1e6u	A	TYR136, LYS140, SER107, SER108, CYS109, HIS179
65	1ea0	A	CYS1, ASN231, GLY232
66	1ecf	A	CYS1, ASN101, GLY102, GLY27, TYR258
67	1ef8	A	HIS66, GLY110, TYR140
68	1eh6	A	GLU172, HIS146, CYS145, TYR114, ASN137
69	1et0	A	THR38, LYS159, GLU193
70	1eug	A	PHE77, TYR66, HIS187
71	1eyq	A	THR48, TYR106
72	1ezf	A	TYR171, ARG228, ARG218, PHE288
73	1f2v	A	HIS43
74	1f7u	A	HIS159, LYS156, HIS162
75	1fbx	A	HIS112, HIS179
76	1fcb	A	TYR254, ASP282, HIS373
77	1fua	A	GLU73
78	1fui	A	GLU337, ASP361
79	1g72	A	ASP297
80	1ggt	A	TRP279, CYS314, HIS373, ASP396, TYR560
81	1gim	A	ASP13, HIS41, GLN224, LYS16
82	1gog	A	TYR495, HIS581, TYR272, HIS496, CYS228
83	1gpm	B	LYS381, ASP239, GLU183, HIS181, CYS86, GLY59, TYR87
84	1gsa	A	LYS160, ARG210, ARG225
85	1gtk	A	ASP84, LYS83, ARG131, ARG132, CYS242
86	1gu1	A	PRO15, ASN16, ARG23, TYR28, ASN79, ALA82, GLU104, HIS106, ARG113
87	1gzg	A	LYS205, LYS260
88	1h1z	A	ASP178, ASP38, SER11
89	1h3i	A	TYR335, TYR245, HIS297, HIS293, TYR305
90	1hfe	L	GLU156, GLU159, CYS178, SER198, LYS237, GLU240, GLU245
91	1hpl	A	SER152, HIS263, ASP176, PHE77, LEU153
92	1hs6	A	GLU271, ASP375
93	1hxq	A	ASN153, SER161, HIS164, HIS166, GLN168
94	1hyo	A	HIS133, GLU199, ARG237, GLN240, LYS253, GLU364
95	1hzd	A	ALA141, GLY186, GLU189, GLU209
96	1hzy	A	ASP233, HIS254, ASP301
97	1idt	A	LYS14, LYS74
98	1igs	A	LYS53, GLU51, LYS110, GLU159, ASN180, SER211
99	1ile	A	LYS591, LYS594, PRO46, TRP518, TRP558, ASP85, GLN554
100	1ir3	A	ASP1132, ARG1136
101	1itq	A	HIS152, ASP288

102	1ivh	A	MET135, SER136, GLU254
103	1j09	A	LYS246
104	1j21	A	SER173, ARG92
105	1jc5	A	GLU48, GLU141
106	1jdw	A	ASP170, ASP254, HIS303, ASP305, CYS407
107	1jfg	B	TYR93, ARG182, LYS232, ARG304, TYR305, THR96, LEU97, ASP100
108	1kc7	A	ARG92, LYS22, HIS455, ARG337, MET103, GLY101, TYR851, SER764, CYS831
109	1kei	A	GLU143, TYR157, ASP226, HIS231
110	1kq3	A	HIS255
111	1kzl	A	THR50, SER41, CYS48, THR148, HIS102
112	1l8s	A	GLY30, HIS48, TYR52, TYR73, ASP99
113	1lcb	A	GLU60, TYR146, CYS198, ARG218, ASP221, TRP82
114	1lij	A	ARG136, ASP318
115	1ljl	A	CYS10, THR11, GLY12, ASN13, SER14, CYS15, ARG16, SER17, CYS82, CYS89, ASP105
116	1lok	A	GLU151
117	1luc	A	HIS44, HIS45
118	1lwy	A	LYS249, ASP268
119	1lxa	A	HIS125, GLY143, ASP126
120	1m4l	A	ARG127, GLU270
121	1m6k	A	TRP160, ALA215, SER67, SER115, SER120
122	1m9c	A	ARG55, PHE60, GLN63, ASN102, PHE113, LEU122, HIS126
123	1mb9	A	TYR348, GLU382, LYS443
124	1mek	A	CYS36
125	1mg5	A	TYR152, LYS156, SER139
126	1mht	A	CYS81, ARG163, ARG165, GLU119
127	1mki	A	LYS77, TYR253, TYR201, VAL271
128	1mla	A	SER92, LEU93, HIS201, ARG117, GLN250, GLN11
129	1mns	A	LYS164, LYS166, ASP270, HIS297, GLU317
130	1mpy	A	HIS199, HIS246, TYR255
131	1muc	A	LYS167, LYS169, GLU327
132	1mum	A	ASP58, CYS123, ARG158, GLU188, ASN210
133	1n1d	A	LYS44, LYS46
134	1n20	A	TRP323, PHE578, ILE344, VAL452
135	1n62	B	CYS388, GLU763
136	1nba	A	ASP51, LYS144, ALA172, THR173, CYS177
137	1ndp	B	HIS122, LYS16, TYR56, ASN119, GLU133
138	1nfs	A	CYS67, GLU116, TRP161, TYR104
139	1nzy	A	PHE64, ALA86, HIS90, GLY114, TRP137, ASP145
140	1o8a	A	HIS353, ALA354, GLU384, HIS513, TYR523
141	1odm	A	PHE211
142	1ofd	A	CYS1, ARG31, PHE207, ASN227, GLY228, GLU903, GLN969, LYS972, GLN978
143	1onr	A	ASP17, GLU96, LYS132, THR156
144	1os7	A	ARG270
145	1oth	A	ARG141, HIS168, GLN171, ASP263, CYS303, ARG330
146	1oya	A	HIS191, ASN194, ASN251, TYR196, THR37
147	1p5d	X	LYS118, HIS109, HIS329, ARG20, ARG247
148	1pfq	A	TYR547, SER630, TYR631, ASP708, HIS740
149	1pjb	A	LYS74, TYR93, HIS95, GLU117
150	1pkn	A	ARG119, ARG72, LYS269, THR327

151	1pq5	A	HIS56, ASP99, GLN192, GLY193, ASP194, SER195
152	1ps1	A	PHE76, PHE77, ASN219, TRP308, HIS309
153	1ps9	A	GLU164, TYR166, ARG214, HIS252, GLN339
154	1ptd	A	HIS32, ASP33, ARG69, HIS82, ASP274
155	1pwh	A	LYS41, ALA222, CYS303
156	1pym	A	ARG159, SER46, LEU48, SER123, ASN122, HIS190
157	1q0n	A	ARG82, ARG92
158	1qfe	A	GLU86, HIS143, LYS170
159	1qh5	A	ASP58
160	1ql0	A	ARG57, HIS89, ASN119, GLU127
161	1qlh	A	SER48, HIS51
162	1qq5	A	ASP8, THR12, ARG39, ASN115, LYS147, SER171, ASN173, PHE175, ASP176
163	1qrr	A	TYR182, LYS186, THR145, HIS183, SER180
164	1qum	A	GLU261
165	1rdd	A	HIS124
166	1req	A	TYR89, HIS244, LYS604, ASP608, HIS610
167	1rhs	A	SER274, VAL251, LYS249, THR252, GLY250, ARG186, ARG248
168	1rql	A	ASP12, ALA45, MET49, LYS53, HIS56, ARG160
169	1ru4	A	ASN268, LYS273
170	1ruv	A	HIS12, LYS41, HIS119, PHE120, ASP121
171	1ryp	I	THR1, ASP17, ARG19, LYS33, GLY47, SER129, ASP166, SER169
172	1s6l	A	CYS96, ASP99, CYS159
173	1s9i	A	ASP194, ASP221
174	1sdw	A	HIS108, GLN170
175	1sg4	A	LEU66, GLY111, GLU136
176	1sml	A	ASP120, TYR191
177	1sox	A	TYR322
178	1szd	A	HIS135, ARG45, PHE44, ASP43, PRO42, ASP118, ASN116
179	1ti6	A	HIS144, ASP174, TYR404
180	1uag	A	LYS115, ASN138, HIS183
181	1ula	A	HIS86, GLU89, ASN243
182	1uw8	A	GLU162, ARG92
183	1v25	A	LYS439, TRP444
184	1v54	A	ASP91, TRP126, SER156, SER157, HIS240, GLU242, TYR244, SER255, HIS291, THR316, LYS319, ARG438
185	1v97	A	GLU802, ARG880, GLU1261
186	1vao	A	TYR108, ASP170, TYR503, ARG504, HIS422
187	1vas	A	THR2, ARG22, GLN23, ARG26
188	1vlb	A	GLU869
189	1vr7	A	SER63, GLU62, HIS68, CYS83
190	1w1o	A	HIS105, ASP169, GLU288
191	1xel	A	SER124, TYR149, LYS153
192	1xva	B	GLU15
193	1xx2	A	SER64, TYR150, SER318, GLU272, LYS67, LYS315
194	1yq4	A	PHE130, LEU263, GLU266, GLN251, HIS253, HIS364, ARG408, ARG297
195	1yrc	A	ARG186, ASP251, THR252, CYS357, LEU358, GLY359
196	1yt3	A	ASP155, TYR151
197	1ytw	A	GLU290, ASP356, HIS402, CYS403, ARG409, THR410
198	1z9h	A	TYR107, CYS110, PHE112, CYS113
199	1ze1	A	ASP39, TYR67
200	1znb	A	ASN193

201	2a84	A	HIS44, HIS47, SER196, SER197, ARG198
202	2adm	A	ASN105, PRO106, TYR108
203	2ahj	B	ARG56
204	2b3i	A	HIS39, HIS85, MET90, CYS82
205	2bbk	L	TRP108, ASP32, ASP76
206	2c3m	A	THR31, GLU64, ARG114, ASN996
207	2c7v	A	ARG14, ASP161, TYR174
208	2cpo	A	CYS29, ASP106, HIS105, GLU183
209	2dd5	C	CYS128, SER132, SER108
210	2eua	A	LYS420, GLU416, GLU284, LYS190, GLU240
211	2f9r	A	HIS47, HIS12, GLY48, ASP34, ASN252, ASP233, ASP52, TRP230, LYS93
212	2frv	B	CYS530, ARG463, GLU18, CYS65, CYS533, CYS68, SER486, HIS72
213	2gb0	A	THR48, LYS265, HIS269, CYS315, LYS348
214	2gmh	A	ARG331, THR367, SER82, CYS553, CYS559, CYS556, CYS528
215	2gsa	A	ASP245, LYS273
216	2gtr	A	LEU66, LEU115, ASP146
217	2jcw	A	HIS63, ARG143
218	2mas	A	ASP10, ASN168, HIS241
219	2pan	A	VAL51, LEU421, LEU476, ILE479, VAL25
220	2pfl	A	TRP333, CYS418, CYS419, GLY734
221	2pz0	A	ASP239, HIS17, HIS59, ARG18, LYS121
222	2qf7	A	ASP549, ASP655
223	2rjr	A	TYR63, GLY70
224	2sns	A	ARG35, GLU43, ARG87
225	2tmd	A	TYR169, HIS172, CYS30
226	2toh	A	HIS331, SER395
227	2ts1	A	THR40, HIS45, HIS48, LYS82, ARG86, GLN173, LYS230, LYS233, THR234, ASP194
228	2vck	A	ASP105, ASP206
229	3bpt	A	GLY98, GLY146, GLU169, ASP177
230	3csm	A	ARG16, ARG157, LYS168, GLU198, GLU246
231	3dfr	A	TRP21, ASP26, THR116
232	3kb9	A	PHE198, PHE95, PHE96, ARG338
233	3pva	A	CYS1, ASN175, TYR82, ARG228
234	5cox	A	GLN203, HIS207, TYR385
235	5eat	A	ARG264, ARG441, THR401, THR402, THR403, TYR527, ASP525, TYR520, ASP444, TRP273
236	5fit	A	GLN83, HIS96, HIS98
237	7atj	A	HIS42, ARG38, ASN70
238	7enl	A	GLU211, LYS345, GLU168, LYS396
239	135l	A	ASP52, GLU35
240	1a0j	A	ASP102, GLY193, HIS57, SER195
241	1a16	A	GLU383, HIS243, HIS350, HIS361
242	1a41	A	ARG223, HIS265, LYS167, TYR274
243	1a4i	A	GLN100, LYS56
244	1a4l	A	ASP295, GLU217, HIS238
245	1a4y	B	HIS114, HIS13, LYS40
246	1a65	A	CYS452, HIS451
247	1a69	A	ARG217, ASP204
248	1a6d	B	ASP391, ASP62, THR95, THR96
249	1a79	D	HIS125, LYS156, TYR115
250	1a8h	A	LYS297, LYS300

251	1a8q	A	ASP223, HIS252, MET95, SER94, TRP28
252	1a8s	A	ASP224, HIS253, SER94, THR95, TRP28
253	1a95	A	ASP88, ASP89, ASP92
254	1aam	A	ASP223, LYS258, TRP142
255	1ab4	A	ARG32, HIS78, TYR122
256	1abr	A	ARG167, GLU164
257	1agm	A	GLU179, GLU400, TRP120
258	1agy	A	ASP175, GLN121, HIS188, SER120, SER42
259	1aj0	A	ARG255, LYS221
260	1aj8	A	ASP312, HIS223, HIS262, SER193
261	1ak0	A	ARG48
262	1akm	A	ARG106, ARG319, ASP231, CYS273, GLN136, HIS133
263	1ald	A	ASP33, GLU187, GLU189, LYS146, LYS229, SER300, TYR363
264	1am2	A	ASN198, ASN74, HIS197, HIS75, SER1, THR72
265	1amy	A	ASP179, ASP289, GLU204
266	1aop	A	ARG153, ARG83, CYS483, LYS215, LYS217
267	1apx	D	ARG38, ASN71, HIS42
268	1aq0	B	GLU232, GLU280, GLU288, LYS283
269	1aql	A	ALA108, ALA195, ASP320, GLY107, HIS435, SER194
270	1ar1	A	GLU278, LYS354
271	1arz	A	HIS159, LYS163
272	1ast	A	GLU93, TYR149
273	1asy	A	ARG325, ARG531, ASP342
274	1at1	A	ARG105, ARG54, HIS134, LYS84, THR55
275	1aug	A	ARG91, CYS144, GLU81, HIS168
276	1aui	A	ARG122, ARG254, ASP121, HIS151
277	1auo	A	ASP168, GLN115, HIS199, LEU23, SER114
278	1avq	B	LYS131
279	1aw8	E	TYR58
280	1ax4	A	ASP133, HIS458, TYR72
281	1ay4	B	ASP222, LYS258, TRP140
282	1azw	A	ASP266, GLY43, HIS294, SER110, TRP111
283	1azy	B	ARG171, HIS85, LYS190
284	1b04	B	ALA114
285	1b2m	A	ARG77, GLU58, HIS40, HIS92, PHE100, TYR38
286	1b2r	A	ALA301, SER80
287	1b5d	A	ASP179, CYS148, GLU60
288	1b5q	A	GLU62
289	1b65	E	ASN218, GLY289, SER250, SER288, TYR146
290	1b66	B	CYS42, GLU133
291	1b6g	A	ASP124, ASP260, HIS289
292	1b73	A	ASP7, CYS178, CYS70, SER8
293	1b7y	A	ALA314, ARG204
294	1b8f	A	GLU414, TYR280
295	1b8g	A	ASP230, LYS273, TYR145
296	1b93	B	ASP101, ASP71, ASP91, GLY66, HIS19, HIS98
297	1b9h	A	ASP159, LYS188, PHE88
298	1bbs	B	ASP215, ASP32, SER35
299	1bd3	A	ARG137, ASP235, THR141
300	1be1	A	ASP14, HIS16
301	1bf2	A	ASP375, ASP510, GLU435
302	1bfd	A	GLU28, HIS281, HIS70

303	1bg6	A	ASP297, HIS202
304	1bgl	H	GLU461, GLU537
305	1bh2	A	ARG178, GLN204, GLU43, THR181
306	1bhg	A	GLU451, GLU540, TYR504
307	1bib	A	ARG118, ARG317, LYS183
308	1bix	A	ASP210, ASP283, HIS309, TYR171
309	1bjo	A	ASP174, LYS198, TRP102
310	1bjp	B	PHE50, PRO1
311	1bo1	B	ASP278, LYS150
312	1bol	A	GLU105, HIS109, HIS46
313	1boo	A	ASP96, PRO54, SER53
314	1bou	B	HIS195
315	1bqc	A	ASN127, GLU128, GLU225, HIS196, TYR198
316	1brm	A	CYS135, GLN162, HIS274
317	1bs0	A	ASP204, GLU175, HIS133, LYS236
318	1bs9	A	ASP175, GLN91, HIS187, SER90, THR13
319	1bu7	B	CYS400, PHE393, THR268
320	1bvv	A	GLU172, GLU78
321	1bvz	B	ASP325, ASP421, GLU354, TRP356
322	1bwd	B	ARG127, ASP108, ASP179, ASP229, CYS332, HIS227, HIS331
323	1bwz	A	CYS217, CYS73, GLU208, HIS159
324	1bxr	D	CYS269, HIS353
325	1bya	A	ASP101, GLU186
326	1bzc	A	ARG221, ASP181, CYS215, SER222
327	1c17	L	ASP61
328	1c2t	A	ASN106, ASP144, HIS108, SER135
329	1c3j	A	ASP100, GLU22
330	1c4t	C	HIS375, THR323
331	1c4x	A	ASP235, HIS263, SER110
332	1c4z	A	ARG506, ASP607, CYS820, GLU539, GLU550, HIS818
333	1c54	A	ARG65, GLU54, HIS85
334	1c82	A	ASN349, HIS399, TYR408
335	1cb7	B	GLU171
336	1cb8	A	ARG288, HIS225, TYR234
337	1cbg	A	ASN324, GLU183, GLU397
338	1cel	A	ASP214, GLU212, GLU217, HIS228
339	1cev	B	ASP126, GLU271
340	1cf2	R	CYS140, HIS219
341	1cfr	A	LYS190
342	1cg2	A	ASP141, GLU175, GLU176, GLU200, HIS112, HIS385
343	1cgk	A	ASN336, CYS164, HIS303, PHE215
344	1chd	A	ASP286, HIS190, MET283, SER164, THR165
345	1chk	A	ASP40, GLU22, THR45
346	1ci8	A	LYS78, SER75, TRP348, TYR181, VAL351
347	1cjy	A	ASP549, GLY197, GLY198, SER228
348	1ck7	A	ALA404
349	1cl1	A	TYR111
350	1cm0	A	CYS574, GLU570, ILE571, ILE637, LEU606, PHE563, PHE568, TYR640, VAL572
351	1cmx	A	ASP181, CYS90, GLN84, HIS166
352	1cns	A	GLU67, GLU89
353	1coy	A	ASN485, GLU361, HIS447

354	1cqg	A	ALA35, CYS32
355	1cqq	A	CYS147, GLU71, GLY145, HIS40
356	1ctn	A	GLU315
357	1cv2	A	ASP108, GLU132, HIS272, TRP109
358	1cvr	A	CYS244, GLU152, GLY212, HIS211
359	1cw0	A	ASP97, HIS69
360	1cwy	A	ASP293, ASP395, GLU340
361	1cz0	A	ARG61, CYS105, HIS98
362	1cz1	A	GLU192, GLU292
363	1czf	A	ASP180, ASP201, ASP202, HIS223
364	1d1q	B	ALA13, ARG19, ASP132, SER20
365	1d2r	A	LYS192, LYS195
366	1d2t	A	ARG183, ASP193, HIS150, HIS189
367	1d3g	A	LYS255, PHE149, SER215, THR218
368	1d4c	A	ARG401, ARG544, GLU377, HIS503, HIS504
369	1d5r	A	ARG130, ASP92, CYS124
370	1d6o	A	ASP37, ILE56, TYR82
371	1d8h	A	ARG393, LYS409, LYS456
372	1d8t	A	ASP21
373	1db3	A	GLU134, LYS160, THR132, TYR156
374	1dbf	A	ARG90
375	1dco	A	GLU58, GLU81, HIS62, HIS63, HIS80
376	1ddj	B	ALA741, ASP646, HIS603
377	1de3	A	GLU96, HIS137, HIS50
378	1de6	A	ASP302, HIS270, LYS236
379	1dek	A	ARG68, HIS206
380	1dgk	N	ASP657
381	1dhf	A	GLU30, LEU22
382	1dhr	A	LYS150, TYR146
383	1dii	A	ARG474, GLU380, HIS436, TYR473
384	1din	A	ASP171, CYS123, HIS202, ILE37, LEU124
385	1dio	L	ASP335, GLN296, GLU170, HIS143
386	1diz	A	ASP238, TRP272, TYR222
387	1dj0	B	ASP60
388	1dj1	A	ALA48, HIS52, TRP191
389	1djl	B	ARG925, TYR1006, TYR890
390	1dki	D	HIS195, SER47
391	1dl5	A	SER59
392	1dmu	A	LYS144
393	1do6	B	GLU14, LYS15
394	1dpg	B	ASP177, HIS240
395	1ds2	E	ASP102, GLY193, HIS57, SER195
396	1dub	E	ALA98, GLU144, GLU164, GLY141
397	1dup	A	ASP90, ASP92
398	1dve	A	ARG136, ASP140, GLY139, GLY143, HIS25, THR135, TYR58
399	1dwo	B	ASP208, HIS236, SER80, THR11
400	1dxe	A	ARG75, HIS50
401	1dzr	A	ASP170, HIS63
402	1e0c	A	ARG235, GLN231, HIS233, HIS234, SER236, THR232
403	1e19	B	LYS131, LYS215, LYS277
404	1e1a	A	GLU37, HIS287
405	1e2a	A	GLN80, HIS78, HIS82

406	1e2t	A	ARG64, CYS69, GLU39
407	1e3v	A	ASP100, ASP40, TYR16
408	1e5q	H	ASP126
409	1e7l	B	GLU65, HIS41, HIS43
410	1e94	D	GLY45, LYS33, SER124, THR1
411	1eb6	A	GLU129, TYR106
412	1ebf	A	ASP219, LYS223
413	1ec9	A	ASN237, HIS339, LYS205, LYS207
414	1ecl	A	ARG321, GLU9, TYR319
415	1eej	A	ARG125, CYS101, CYS98, TYR100
416	1eh5	A	ASP233, GLN116, HIS289, MET41, SER115
417	1ei5	A	ASN155, HIS287, LYS65, SER62, TYR153
418	1emd	A	ASP150, HIS177
419	1eq2	A	LYS178, SER116, TYR140
420	1esc	A	ASN106, GLY66, HIS283, SER14, TRP280
421	1eso	A	HIS61
422	1eu1	A	TRP116, TYR114
423	1euu	A	ASP92, GLU260, TYR370
424	1euy	A	ARG260, GLU34, LYS270
425	1evy	A	LYS210, THR267
426	1ex1	A	ASP285, GLU491
427	1exn	A	ARG86, LYS83
428	1exp	A	ASN169, ASP235, GLU127, GLU233, HIS205
429	1eyi	A	ASP68, ASP74, GLU98
430	1ez1	B	ARG362, ASP286, GLY162, SER160, THR287
431	1f2d	A	LYS51, TYR295, TYR269
432	1f48	A	GLY18, LYS21
433	1f6d	A	ARG215, ASP95, GLU117, GLU131, HIS213, HIS246
434	1f75	A	ASP29, HIS46
435	1f7l	A	HIS105, LYS62
436	1f8m	A	ARG228, CYS191, HIS180, HIS193
437	1f8r	A	HIS223, LYS326
438	1f8x	A	ASP72, ASP92, GLU98, TYR7
439	1fa0	A	LYS215
440	1fc4	B	HIS213, LYS244
441	1fcq	A	ASP111, GLU113, TRP301, TYR184, TYR227
442	1ff3	A	CYS198, CYS206, GLU94
443	1fft	A	ARG71, ASN124, ASN142, ASP135, ASP75, GLU286, HIS284, HIS419, HIS421, LYS362, MET79, PHE103, PHE420, SER145, SER299, SER315, THR149, THR201, THR204, THR211, THR359, TYR288, TYR61
444	1fgh	A	ARG447, ARG644, ASP100, ASP165, HIS101, SER642
445	1fgj	A	ASP267, HIS233, HIS268, TYR334, TYR467
446	1fhl	A	ARG45, GLU136, GLU246
447	1fnb	A	CYS272, GLU312, SER96
448	1foa	A	ASP291
449	1foh	A	ARG281, ASP54, PRO364, TYR289
450	1fps	A	ARG126, PHE253
451	1fq0	A	ARG49, GLU45, LYS133
452	1fr2	B	ARG5, GLU100, HIS102, HIS103
453	1fr8	A	ASP252, ASP318, GLU317
454	1fro	A	GLU172, GLU99
455	1fwk	D	THR183

456	1fy2	A	ALA121, GLU192, GLY88, HIS157, SER120
457	1g0d	A	ASP355, CYS272, HIS332, TYR515
458	1g24	A	GLU214
459	1g64	A	LYS41, THR43
460	1g6t	A	ASP313, GLU341, HIS385, LYS411
461	1g79	A	ARG197
462	1g8o	A	GLU317
463	1g8p	A	ARG289
464	1g99	A	ARG241, ARG91, ASP148, GLY212, SER10
465	1ga8	A	ASP130, ASP188, GLN189
466	1gal	A	HIS516
467	1gcb	A	ASN392, CYS73, GLN67, HIS369
468	1gcu	A	ARG171, GLU123, GLU126, GLU96, SER170, TYR97
469	1gdh	A	GLU269, HIS287
470	1ge7	B	GLU118, TYR133
471	1geq	A	ASP47, GLU36, TYR161
472	1glo	A	ASN184, GLN19, HIS164, SER25
473	1goj	A	GLY238
474	1gp1	A	GLN80, TRP158
475	1gp5	A	LYS213
476	1gpa	D	ARG569, LYS568, LYS574, THR676
477	1gpj	A	HIS84, SER48
478	1gpr	A	HIS68, HIS83, THR66
479	1gq8	A	ASP136, ASP157, GLN113, GLN135
480	1gqg	C	GLU73
481	1gt7	P	GLU117, GLU171
482	1guf	B	TYR79
483	1gxs	A	SER158, TRP270
484	1gz6	D	LYS168, TYR164
485	1h4g	B	GLU184, GLU94
486	1h54	B	GLU487
487	1h7a	A	ALA580, ASN311, CYS290, CYS79, GLU446
488	1h7o	A	LYS210, LYS263
489	1h7x	D	ALA671
490	1hdh	A	ARG55, ASP317, HIS115, HIS211, LYS113, LYS375
491	1hpm	A	LYS71
492	1hqc	A	ARG205, THR146
493	1hr6	A	ASP65
494	1hr7	H	GLN73
495	1hrd	A	ASP165, LYS125
496	1hrk	A	ARG164, ASP340, GLU343, GLU347, HIS263, TYR165
497	1hti	A	ASN11, GLU165, HIS95, LYS13
498	1hto	X	ARG339, ASP50, GLU327
499	1hv9	B	ARG18
500	1hy3	A	HIS107, LYS47, SER137
501	1hzf	A	CYS991, GLN994
502	1i19	B	ARG477, GLU311, GLU475
503	1i1e	A	ARG369, GLU267, TYR372
504	1i1i	P	GLU503, TYR613
505	1i6p	A	ARG46, ASP44
506	1i78	A	ALA99, ASP210, ASP83, ASP85, HIS212
507	1i8d	A	CYS48, HIS102, MET64, PHE2, SER41

508	1i8t	A	ARG170, ARG278, ASP348, GLU298
509	1idj	A	ARG176, ARG236, ASP154, LYS239
510	1ig8	A	ARG173, ASP211, SER158
511	1im5	A	ALA129, ASP10, CYS133, LYS94
512	1ima	A	GLU70, THR95
513	1iph	A	ASN201, HIS128, HIS392
514	1it4	A	ASP85, HIS64
515	1itx	A	ASP200, GLU204
516	1iu4	D	ASP255, CYS64, HIS274, LYS269, TRP272
517	1iyd	B	LYS159
518	1j00	A	ASN73, ASP154, GLY44, HIS157
519	1j2u	A	GLU122, HIS178
520	1j49	A	ARG236, ASP260, GLU265, HIS297
521	1j53	A	GLU14, GLU61, HIS162
522	1j70	C	ARG197, HIS201, HIS204
523	1j79	B	ASP250
524	1j7g	A	GLN78, PHE79, THR80
525	1jch	C	ARG545, ASP510, GLU517, HIS513
526	1jen	A	CYS82
527	1jfl	A	CYS194, CYS82
528	1jh6	A	HIS119, HIS42, MET117, SER121, TYR124
529	1jhf	A	ASP127, GLU152, LYS156, MET118, SER119
530	1jkm	A	ASP308, HIS338, SER202
531	1jm6	B	GLU1243, HIS1239
532	1jms	A	ASP434
533	1jnr	C	ARG265, ASN74, ASP361, GLU141, HIS398, SER449, TRP234
534	1joa	A	ARG303, HIS10
535	1jof	E	ARG196, ARG274, GLU212, HIS148
536	1jrp	D	ARG310, GLU730
537	1js4	B	ASP55, ASP58, GLU424
538	1jxh	B	GLY210, LYS176
539	1k0w	A	ASN120
540	1k30	A	ASP144, HIS139
541	1k32	D	ASP966, GLY918, HIS746, SER965
542	1k4l	A	ASP41, ASP99, CYS66, GLU174, HIS136, TYR94
543	1k4t	A	ARG488, ARG590, HIS632, LYS532
544	1k82	D	ARG258, GLU2, LYS56, PRO1
545	1kae	A	GLU326, HIS327
546	1kas	A	CYS163, GLU314, HIS303, HIS340, LYS335, PHE400
547	1kcz	A	HIS194, LYS331
548	1kdg	A	ASN732, HIS689
549	1kfu	L	ASN286, CYS105, GLN99, HIS262, TRP288
550	1kim	A	ARG163, ARG220, ARG222, GLU225, GLU83, LYS62
551	1kl7	A	LYS124
552	1knp	A	ARG290, HIS351, LEU386
553	1kny	A	GLU145, LYS149
554	1kp2	A	ARG106, ASP135
555	1kra	C	ARG336, ASP221, HIS219, HIS320
556	1ksj	A	GLN70
557	1kws	A	GLU281
558	1kyq	A	ASP141
559	1kyw	F	HIS269

560	1kzh	A	ARG146, ASP206, GLY82, LYS203, THR204
561	1l0o	B	ARG105, GLU46
562	1l1d	B	ARG493, ASP484, CYS440, CYS495, HIS480
563	1l1l	A	CYS119, CYS408, CYS419, GLU410
564	1l1r	A	ARG63, GLU100
565	1l6p	A	ASP68, CYS103, CYS109, PHE70, TYR42, TYR71
566	1l7d	D	ARG1327, ASP1335, GLN1332, SER1338
567	1l7n	B	ASP511, ASP513, ASP671, GLY600, LYS644, PHE512
568	1l7q	A	ALA117, ASP259, HIS287, TRP166, TYR118, TYR44
569	1l8t	A	ASP190, LYS44
570	1l9x	D	CYS110, HIS220
571	1lam	A	ARG336, LYS262
572	1lba	A	LYS128, TYR46
573	1lbu	A	ASP194, HIS192, HIS195, TYR189
574	1lml	A	GLU265
575	1lnh	A	ASN713
576	1ltq	A	ARG126, ASP165, ASP35, LYS15
577	1lvh	A	LYS145, SER114
578	1lws	A	LYS301, LYS403
579	1lz1	A	ASP53, GLU35
580	1m21	B	LYS123, SER202, SER226
581	1m54	B	LYS119
582	1mbb	A	ARG159, GLU325, SER229
583	1mfp	B	LYS1163, TYR1156
584	1mhy	D	CYS151, THR213
585	1mj9	A	GLU338, SER304
586	1mka	A	ASP84, CYS80, GLY79, HIS70, VAL76
587	1mlv	C	TYR287
588	1mok	D	CYS82, CYS87, HIS137, LEU78, PHE501
589	1moq	A	GLU481, GLU488, LYS485, LYS603
590	1mpx	A	ASP307, HIS340, SER174, TYR175, TYR82
591	1mqw	A	ARG64, GLU142
592	1mrq	A	ASP50, HIS117, LYS84, TYR55
593	1mt1	L	GLU109
594	1mt5	A	GLY239, GLY240, ILE238, LYS142, SER217, SER218, SER241
595	1mud	A	ASN138, GLU37
596	1mug	A	ASN18
597	1mvn	A	HIS90, SER175
598	1myr	A	ARG95, ASN328, GLN187, GLU409, SER190, TYR330
599	1n29	A	ASP91, GLY29, HIS47
600	1n2c	F	ASP129, LYS10
601	1n2t	A	ALA199, ALA223, ARG360, ASP197, GLN200, HIS114
602	1nbf	B	ASN218, ASP481, CYS223, HIS464
603	1ndh	A	TYR65
604	1ndi	B	HIS343, SER554
605	1nf9	A	ASP38
606	1nhx	A	ARG405, HIS264
607	1nid	A	ASP98, HIS255
608	1nir	B	HIS327, HIS369
609	1nkk	A	ARG165, HIS157, HIS63, SER132
610	1nln	A	CYS122, GLN115, GLU71, HIS54
611	1nlu	A	ASP170, ASP84, GLU80, SER287

612	1nml	A	GLU114
613	1nmw	A	CYS113, HIS157, HIS59
614	1nn4	A	ARG137, ASP9, CYS66, HIS10
615	1nsf	A	LYS549, LYS631, LYS708
616	1nsj	A	ASP126, CYS7
617	1nvm	A	HIS21, TYR291
618	1nvt	A	ASP102
619	1nw9	B	ARG178, CYS287, GLY238, HIS237
620	1nww	A	ARG99, ASN55, ASP101, ASP132, TYR53
621	1o04	C	GLU268, GLU399, LYS192, SER302
622	1o98	A	ARG261, ASP154, SER62
623	1o9i	A	GLU178
624	1oac	A	ASP383
625	1oas	B	LYS41, SER272
626	1oba	A	ASP10, ASP182, ASP92, GLU94
627	1odt	C	ALA181, ASP269, GLN182, HIS298
628	1oe8	A	TYR10
629	1ofg	B	LYS129, TYR217
630	1og1	A	ARG184, GLU159, GLU189, SER147
631	1ogo	X	ASP376, ASP395
632	1oh9	A	GLY11, GLY45, LYS217, LYS8
633	1ohh	C	GLN208, LYS175, LYS209
634	1ohv	D	ASP298, LYS329, PHE189
635	1oj4	A	ASP141, LYS10
636	1ok4	J	ASP24, LYS177, TYR146
637	1okg	A	ASP61, HIS75, SER255
638	1or8	A	GLU144, GLU153
639	1ord	A	ASP316, HIS223, LYS355
640	1oro	A	LYS103
641	1otg	A	ARG41, PHE35, PRO2
642	1oxa	A	GLU360, SER246
643	1oyg	A	ASP247, ASP86, GLU342
644	1ozh	D	MET394, MET422
645	1p1x	B	ASP1102, LYS1167, LYS1201
646	1p3d	A	LYS129
647	1p4n	A	ARG211, ASP108, GLU319, LYS36
648	1p7m	A	GLU38, TRP46, TYR16
649	1pad	A	ASN175, CYS25, HIS159
650	1pbg	A	GLU160, GLU375
651	1pd2	2	ARG14, TRP104, TYR8
652	1peg	A	TYR178, TYR283
653	1pfk	A	ARG171, ARG72, ASP127, GLY11, THR125
654	1pgs	A	ASP60, GLU206
655	1pii	A	ASN184, GLU163, GLU53, LYS114, LYS55, SER215
656	1pix	B	ALA457, GLY194, ILE417, VAL151
657	1pj5	A	ASP552, HIS225, TYR259
658	1pja	A	ASP228, GLN112, HIS283, LEU45, SER111
659	1pjh	B	ALA70, GLU158, LEU126
660	1pjq	B	ASP248, LYS270, MET382
661	1pma	N	GLY80, LYS66, SER167, SER35
662	1pmi	A	ARG304, GLN111, GLU294
663	1pnl	B	ALA69, ASN241, SER1

664	1pnt	A	ARG18, ASP129, CYS12, CYS17
665	1pow	B	ARG264, GLU483, PHE479
666	1pp4	A	ASN74, ASP192, GLY42, HIS195, SER9
667	1psd	A	GLU269, HIS292
668	1pud	A	ASP102
669	1pvd	B	ASP28, GLU477, HIS114, HIS115
670	1pvi	B	LYS70
671	1pwv	B	GLU687, TYR728
672	1pyl	B	ARG67, GLU56, HIS86
673	1pz3	A	GLU175
674	1q18	B	ASP100
675	1q3n	A	HIS202
676	1q3q	A	ASP393, ASP64, THR97, THR98
677	1q6l	B	ARG139, GLU112, HIS136, ILE37, LYS64, THR36
678	1q6x	B	HIS334, PRO108, SER550, TYR95
679	1q91	A	ASP41, ASP43
680	1qam	A	ASN101, GLU59, GLY38
681	1qaz	A	ARG239, ASN191, TYR246
682	1qb4	A	ARG396, ARG581, ARG713
683	1qba	A	ASP539, GLU540
684	1qd1	A	HIS82
685	1qe3	A	GLU310, HIS399, SER189
686	1qf6	A	ARG363
687	1qfl	A	CYS378, HIS348
688	1qfm	A	ASP641, HIS680, SER554
689	1qfn	A	ARG8, GLY10, LYS18, TYR13, TYR72
690	1qgn	H	ASP236, LYS261, TYR163
691	1qgx	A	ASP294, ASP49, THR147
692	1qh9	A	ARG41, ASP10, ASP180, SER118
693	1qhf	B	ARG59, GLU86, HIS181, HIS8
694	1qhg	A	ARG610, GLU224, LYS37
695	1qho	A	ASP228, ASP329, GLU256, HIS132
696	1qi9	B	HIS411, HIS418, HIS486, LYS341
697	1qk2	B	ARG174, ASP175, ASP221, TYR169
698	1qmh	B	HIS309
699	1qol	H	ALA51, ASP163, HIS148
700	1qrg	A	ASN202, GLN75, GLU62, GLU84
701	1qtn	A	ARG258, CYS360, GLY350, HIS317
702	1qv0	A	HIS175, HIS22, TRP92, TYR138, TYR190
703	1qwn	A	ASP204, ASP341
704	1qx3	A	CYS163, GLY122, HIS121
705	1qz9	A	ASP201, LYS227, PHE129
706	1r16	B	GLU179, GLU98
707	1r1j	A	ARG717, ASP650, GLU584, HIS711
708	1r30	B	ARG260, CYS53, CYS57, CYS60
709	1r44	B	ARG71, GLU181
710	1r4f	A	ALA260, ASN186, ASP10, TRP83
711	1r4z	A	ASP133, HIS156, ILE12, MET78, SER77
712	1r6w	A	ARG133, LYS235
713	1r76	A	ARG307, ASN172
714	1ra0	A	GLN156, GLU217
715	1ra2	A	ASP27, ILE5, ILE94, LEU28, LEU54, MET20, PHE31

716	1rba	A	ASN192, HIS287, HIS321, LYS166, LYS191
717	1rbl	A	ASP203, HIS294, HIS327, LYS175, LYS177, LYS201
718	1rgq	B	ASP84, GLY140, HIS60, SER142
719	1rhc	A	GLU108, HIS39, TRP43
720	1rk2	D	ALA252, ALA253, ASP255, GLY254
721	1ro7	A	ARG129, HIS188, TYR156, TYR162
722	1roz	A	GLU137, HIS288, LYS329
723	1rpt	A	ARG11, ARG15, ARG79, ASP258, HIS12, HIS257
724	1rpx	B	ASP185, ASP43, HIS41, HIS74
725	1rtf	B	ASP102, GLY193, HIS57, SER195
726	1rtu	A	GLU62, HIS101, HIS41
727	1rvv	J	HIS88
728	1s20	A	HIS44
729	1s2k	A	GLN53, GLU136
730	1s3i	A	ASP142, HIS106
731	1s76	D	LYS631
732	1s95	A	ARG275, ARG400, ASN303, ASP274, HIS304, HIS427
733	1s9c	C	ASP193, GLY216, HIS198, ILE213
734	1sca	A	ASP32, HIS64, SER221
735	1ses	B	ARG256, ARG271, ASP265, GLU258, SER261
736	1sll	A	ASP318, GLU595, TYR713
737	1slm	A	GLU202
738	1sme	A	ASP214, ASP34, SER37, THR217
739	1snn	B	ASN106, ASP30, CYS55, GLU185, SER147, TYR95
740	1snz	B	GLU307, HIS176
741	1ssx	A	ASP102, GLY193, HIS57, SER195, SER214
742	1stc	E	ASP166, LYS168, THR201
743	1std	A	ASP31, HIS110, HIS85, TYR30, TYR50
744	1su4	A	ASP351
745	1szj	G	CYS149, HIS176
746	1t0u	A	ARG223, GLU80, HIS8
747	1t7d	A	LYS145, SER88, SER90
748	1tde	A	ASP139, CYS135, CYS138
749	1tdj	A	LYS62, SER315
750	1thg	A	ALA132, ALA218, GLU354, HIS463, SER217
751	1tht	A	HIS241, SER114
752	1tml	A	ASP117, ASP265
753	1tmo	A	SER149
754	1tox	A	GLU148
755	1trk	A	HIS263, HIS30
756	1tyf	M	ASP171, GLY68, HIS122, MET98, SER97
757	1tys	A	ARG166, ASP169, GLU58, SER146, TYR94
758	1tz3	A	ALA250, ASP252, GLY249, GLY251
759	1u3f	B	ARG115
760	1u5u	B	ASN137, HIS67, THR66, TYR193
761	1u7u	A	ASP210
762	1u8v	C	HIS292
763	1uae	A	ARG397, ASN23, ASP305, CYS115
764	1uam	A	ASP169
765	1uaq	B	CYS91, GLU64, SER89
766	1uas	A	ASP130, ASP185
767	1uch	A	ASP184, CYS95, GLN89, HIS169

768	1uf7	A	ALA171, GLU46, LYS126
769	1ujn	B	ARG224, ASN228, GLU220, HIS235, LYS131, LYS210
770	1uk7	A	ALA103, ASP224, HIS252
771	1un1	B	ASP87, GLU85, GLU89
772	1uok	A	ASP199, ASP329, GLU255
773	1uqr	J	ARG107, ARG18, ASN11, GLU98, HIS100, TYR23
774	1uqt	B	ASP361, HIS154
775	1uro	A	ASP86, TYR164
776	1ush	A	ARG375, ARG379, ARG410, ASN116, ASP120, HIS117
777	1v04	A	HIS115, HIS134
778	1v0e	C	ARG596, ARG647, GLU581
779	1v0y	A	ASP202, ASP473, HIS170, HIS448
780	1vid	A	GLU199, LYS144
781	1vie	A	GLN67, ILE68, LYS32, TYR69
782	1vnc	A	HIS404, LYS353
783	1vom	A	ASN233, GLU459, GLY182, GLY457
784	1vq1	A	ASN197, PHE100, PRO198
785	1w0h	A	GLU136, HIS293
786	1w27	B	PHE400
787	1w2n	A	ARG74
788	1wd8	A	ASP350, ASP473, CYS645, HIS471
789	1wgi	B	ASP117
790	1wnw	B	ASN136, GLY140
791	1x7d	A	ASP228, GLU56
792	1x9h	B	ARG135, GLU203, HIS219, LYS298
793	1x9y	B	ASN360, CYS243, GLN237, HIS340
794	1xa8	C	CYS56
795	1xff	A	ASN98, CYS1, GLY99
796	1xgm	A	GLU187
797	1xik	A	TYR122
798	1xqd	A	ASP393, SER286
799	1xqw	A	ALA105, ASP244, GLY37, HIS271, TYR106
800	1xrs	B	LYS144
801	1xs1	C	ALA124, ARG126, GLU138
802	1xtc	A	ARG7, GLU110, GLU112, SER61
803	1xvt	A	ASP169
804	1xyz	A	ASP756, GLU645, GLU754, HIS723
805	1y9m	A	ASP41, GLU241
806	1ybq	B	ASN285
807	1ybv	A	ASN138, LYS182, SER164, TYR178
808	1ycf	B	HIS25, TYR195
809	1ygh	A	GLU173
810	1yon	A	LYS176
811	1ysc	A	ASP338, HIS397, SER146
812	1yve	L	GLU496
813	1zio	A	ARG127, ARG160, ARG171, ASP162, ASP163, LYS13
814	1zm2	B	GLU553
815	1zoi	B	ALA122, ASP227, HIS256, SER97, THR98, TRP31
816	1zrz	A	ASN374, ASP369, LYS371
817	1zym	A	HIS189, THR168
818	206l	A	ASP20, GLU11
819	2a0n	A	ASP11, ASP130

820	2abk	A	ASP138, LYS120
821	2ace	A	GLU327, HIS440, SER200
822	2acu	A	ASP43, HIS48, LYS77
823	2acy	A	ARG23, ASN41
824	2ag0	A	GLN113, GLU50, HIS29
825	2alr	A	LYS79, TYR49
826	2amg	A	ASP193, ASP294, GLU219
827	2apr	A	ASP218, ASP35, SER38, THR221
828	2ayh	A	ASP107, GLU105, GLU109
829	2bhg	A	ALA163, ASP84, HIS46, SER182
830	2bif	B	ALA256, ARG255, ARG305, ASN262, GLU325, HIS390
831	2bkr	A	ASP119, CYS163, HIS102, TRP103, TRP26
832	2bsx	A	ASP206
833	2bx4	A	CYS145, HIS41
834	2cnd	A	CYS242, PHE270, THR65
835	2cpu	A	ASN300, ASP197, GLU233
836	2dbt	C	ASN194, GLU147, GLU156
837	2dhn	A	GLU22, LYS100
838	2dln	A	ARG255, GLU15, GLY276, SER150, TYR216
839	2dor	B	CYS130, LYS43
840	2dw7	O	ASN55, ASP292, GLU341, HIS322, LYS182, LYS184
841	2ebn	A	ASP130, GLU132
842	2eng	A	ASP10, ASP121
843	2eql	A	ASP53, GLU35
844	2esd	D	ALA250, ASN154, CYS284
845	2f61	A	CYS342, GLU235, GLU340
846	2f9z	C	CYS27, HIS44, THR21
847	2fmn	B	ASP120, GLU28
848	2fok	A	LYS469
849	2hdh	A	ASN208, GLU170, HIS158, SER137
850	2hgs	A	ARG125, ARG450, GLY369, SER151
851	2hi7	B	ARG48, CYS104, CYS41, CYS44
852	2hsa	A	HIS185, HIS188, TYR190
853	2jxr	A	ASP215, ASP32, THR218, THR33
854	2lip	A	ASP264, GLN88, HIS286, LEU17, SER87
855	2nac	B	ARG284, ASN146, GLN313, HIS332
856	2nlr	A	GLU120
857	2nmt	A	ASN169, LEU171, LEU455, PHE170
858	2oat	A	ASP263, LYS292, PHE177
859	2ocp	C	ARG142, GLU70
860	2pec	A	ARG218, ASP131
861	2pgd	A	ASN187, GLU190, GLY130, LYS183
862	2phk	A	ASP149, LYS151
863	2pia	A	ASN44, CYS199, GLU223, SER58
864	2plc	A	ARG84, ASP278, ASP46, HIS45, HIS93
865	2pth	A	ASN10, ASP93, HIS20
866	2rnf	B	HIS116, HIS12, LYS40
867	2sqc	B	ARG127, ASP374, ASP377, CYS376, GLN262, GLU45, GLU93, HIS451, PHE365, PHE601, PHE605, TRP169, TRP312, TRP489, TYR495
868	2tdt	A	ASP141, GLU169, GLY166
869	2thi	A	CYS113, GLU241
870	2tpl	A	ARG381, ASP214, PHE123, TYR71

871	2tps	B	ARG59, LYS159, SER130
872	2xis	A	ASP57, GLU181, HIS54, LYS183
873	3cla	A	ARG18, ASP199, HIS195
874	3eca	D	ASP90, LYS162, THR12, THR89, TYR25
875	3mdd	B	GLU376
876	3nos	B	ARG187, CYS184, GLU361, TRP356
877	3pca	N	ARG457, TYR447
878	3r1r	A	ASN437, CYS225, CYS439, CYS462, GLU441
879	3s8r	B	ALA170, GLU624, VAL239, HIS192, ASN413
880	4kbp	D	HIS202, HIS295, HIS296
881	4mdh	B	ASP158, HIS186
882	7nn9	A	ARG220, ARG371, ASP151, GLU277, TYR412A
883	7odc	A	GLU274, HIS197, LYS69
884	8pch	A	CYS25, GLN19, HIS159

Average (Standard deviation) number of catalytic residues = 3.29 (1.86)

UB-137 dataset (137 proteins)

S.No.	PDB	Chain	Catalytic site residues
1	135l	A	ASP52, GLU35
2	1a4s	A	ASN166, GLU263, CYS297
3	1a4y	B	HIS114, HIS13, LYS40
4	1a6d	B	ASP391, ASP62, THR95, THR96
5	1a7u	A	ASP228, HIS257, SER98, MET99, PHE32
6	1a8q	A	ASP223, HIS252, MET95, SER94, TRP28
7	1ab4	A	ARG32, HIS78, TYR122
8	1agy	A	ASP175, GLN121, HIS188, SER120, SER42
9	1akm	A	ARG106, ARG319, ASP231, CYS273, GLN136, HIS133
10	1ako	A	ASN7, ASP151, ASN153, ASP229, HIS259
11	1ald	A	ASP33, GLU187, GLU189, LYS146, LYS229, SER300, TYR363
12	1am2	A	ASN198, ASN74, HIS197, HIS75, SER1, THR72
13	1aug	A	ARG91, CYS144, GLU81, HIS168
14	1auo	A	ASP168, GLN115, HIS199, LEU23, SER114
15	1azw	A	ASP266, GLY43, HIS294, SER110, TRP111
16	1azy	B	ARG171, HIS85, LYS190
17	1b04	B	ALA114
18	1b65	E	ASN218, GLY289, SER250, SER288, TYR146
19	1b6b	A	SER97, LEU111, HIS122, LEU124, TYR168
20	1b73	A	ASP7, CYS178, CYS70, SER8
21	1bbs	B	ASP215, ASP32, SER35
22	1be1	A	ASP14, HIS16
23	1bg6	A	ASP297, HIS202
24	1bo1	B	ASP278, LYS150
25	1bol	A	GLU105, HIS109, HIS46
26	1bqc	A	ASN127, GLU128, GLU225, HIS196, TYR198
27	1brm	A	CYS135, GLN162, HIS274
28	1bvz	B	ASP325, ASP421, GLU354, TRP356
29	1bwd	B	ARG127, ASP108, ASP179, ASP229, CYS332, HIS227, HIS331
30	1bwp	A	SER47, GLY74, ASN104, ASP192, HIS195
31	1bwz	A	CYS217, CYS73, GLU208, HIS159
32	1c17	L	ASP61
33	1c4x	A	ASP235, HIS263, SER110
34	1c4z	A	ARG506, ASP607, CYS820, GLU539, GLU550, HIS818
35	1c54	A	ARG65, GLU54, HIS85
36	1cbg	A	ASN324, GLU183, GLU397
37	1cfr	A	LYS190
38	1chd	A	ASP286, HIS190, MET283, SER164, THR165
39	1chk	A	ASP40, GLU22, THR45
40	1cns	A	GLU67, GLU89
41	1cqg	A	ALA35, CYS32
42	1ctn	A	GLU315
43	1cv2	A	ASP108, GLU132, HIS272, TRP109
44	1cwy	A	ASP293, ASP395, GLU340
45	1cz1	A	GLU192, GLU292
46	1d2r	A	LYS192, LYS195
47	1d6m	A	GLU7, LYS8, TYR328, ARG330
48	1db3	A	GLU134, LYS160, THR132, TYR156
49	1dco	A	GLU58, GLU81, HIS62, HIS63, HIS80

50	1dd8	A	CYS163, HIS298, LYS328, HIS333, PHE390, PHE392
51	1ddj	B	ALA741, ASP646, HIS603
52	1de3	A	GLU96, HIS137, HIS50
53	1di1	A	TYR92, PHE112, PHE178, LYS206, TRP333
54	1dup	A	ASP90, ASP92
55	1e2t	A	ARG64, CYS69, GLU39
56	1ecl	A	ARG321, GLU9, TYR319
57	1ei5	A	ASN155, HIS287, LYS65, SER62, TYR153
58	1esc	A	ASN106, GLY66, HIS283, SER14, TRP280
59	1eug	A	PHE77, TYR66, HIS187
60	1f2v	A	HIS43
61	1f8x	A	ASP72, ASP92, GLU98, TYR7
62	1fcq	A	ASP111, GLU113, TRP301, TYR184, TYR227
63	1fhl	A	ARG45, GLU136, GLU246
64	1fps	A	ARG126, PHE253
65	1g24	A	GLU214
66	1g8p	A	ARG289
67	1gcu	A	ARG171, GLU123, GLU126, GLU96, SER170, TYR97
68	1geq	A	ASP47, GLU36, TYR161
69	1ggt	A	TRP279, CYS314, HIS373, ASP396, TYR560
70	1glo	A	ASN184, GLN19, HIS164, SER25
71	1gpr	A	HIS68, HIS83, THR66
72	1hrd	A	ASP165, LYS125
73	1hzd	A	ALA141, GLY186, GLU189, GLU209
74	1i8d	A	CYS48, HIS102, MET64, PHE2, SER41
75	1idj	A	ARG176, ARG236, ASP154, LYS239
76	1iu4	D	ASP255, CYS64, HIS274, LYS269, TRP272
77	1j7g	A	GLN78, PHE79, THR80
78	1jfl	A	CYS194, CYS82
79	1jkm	A	ASP308, HIS338, SER202
80	1k30	A	ASP144, HIS139
81	1k32	D	ASP966, GLY918, HIS746, SER965
82	1kas	A	CYS163, GLU314, HIS303, HIS340, LYS335, PHE400
83	1kfu	L	ASN286, CYS105, GLN99, HIS262, TRP288
84	1kra	C	ARG336, ASP221, HIS219, HIS320
85	1l1l	A	CYS119, CYS408, CYS419, GLU410
86	1l6p	A	ASP68, CYS103, CYS109, PHE70, TYR42, TYR71
87	1l7d	D	ARG1327, ASP1335, GLN1332, SER1338
88	1lxa	A	HIS125, GLY143, ASP126
89	1lz1	A	ASP53, GLU35
90	1m9c	A	ARG55, PHE60, GLN63, ASN102, PHE113, LEU122, HIS126
91	1mek	A	CYS36
92	1mla	A	SER92, LEU93, HIS201, ARG117, GLN250, GLN11
93	1nn4	A	ARG137, ASP9, CYS66, HIS10
94	1onr	A	ASP17, GLU96, LYS132, THR156
95	1p1x	B	ASP1102, LYS1167, LYS1201
96	1pgs	A	ASP60, GLU206
97	1pjb	A	LYS74, TYR93, HIS95, GLU117
98	1pma	N	GLY80, LYS66, SER167, SER35
99	1ptd	A	HIS32, ASP33, ARG69, HIS82, ASP274
100	1pvi	B	LYS70
101	1pwv	B	GLU687, TYR728

102	1qfn	A	ARG8, GLY10, LYS18, TYR13, TYR72
103	1qx3	A	CYS163, GLY122, HIS121
104	1rba	A	ASN192, HIS287, HIS321, LYS166, LYS191
105	1s6l	A	CYS96, ASP99, CYS159
106	1s9c	C	ASP193, GLY216, HIS198, ILE213
107	1sll	A	ASP318, GLU595, TYR713
108	1snz	B	GLU307, HIS176
109	1t0u	A	ARG223, GLU80, HIS8
110	1tht	A	HIS241, SER114
111	1tyf	M	ASP171, GLY68, HIS122, MET98, SER97
112	1u7u	A	ASP210
113	1uch	A	ASP184, CYS95, GLN89, HIS169
114	1ujn	B	ARG224, ASN228, GLU220, HIS235, LYS131, LYS210
115	1uok	A	ASP199, ASP329, GLU255
116	1vas	A	THR2, ARG22, GLN23, ARG26
117	1vie	A	GLN67, ILE68, LYS32, TYR69
118	1wd8	A	ASP350, ASP473, CYS645, HIS471
119	1x9y	B	ASN360, CYS243, GLN237, HIS340
120	1xtc	A	ARG7, GLU110, GLU112, SER61
121	1xx2	A	SER64, TYR150, SER318, GLU272, LYS67, LYS315
122	1xyz	A	ASP756, GLU645, GLU754, HIS723
123	1zoi	B	ALA122, ASP227, HIS256, SER97, THR98, TRP31
124	1zym	A	HIS189, THR168
125	2alr	A	LYS79, TYR49
126	2bkr	A	ASP119, CYS163, HIS102, TRP103, TRP26
127	2bx4	A	CYS145, HIS41
128	2eng	A	ASP10, ASP121
129	2eql	A	ASP53, GLU35
130	2f9z	C	CYS27, HIS44, THR21
131	2fok	A	LYS469
132	2gtr	A	LEU66, LEU115, ASP146
133	2pec	A	ARG218, ASP131
134	2plc	A	ARG84, ASP278, ASP46, HIS45, HIS93
135	2pth	A	ASN10, ASP93, HIS20
136	2ts1	A	THR40, HIS45, HIS48, LYS82, ARG86, GLN173, LYS230, LYS233, THR234, ASP194
137	3pva	A	CYS1, ASN175, TYR82, ARG228

Average (Standard deviation) number of catalytic residues = 3.64 (1.58)
