# Supplementary material for: CSmetaPred: a consensus method for prediction of catalytic residues
Source: BMC Bioinformatics. 2017 Dec 22;18:583. doi: 10.1186/s12859-017-1987-z (PMC5741869; doi:10.1186/s12859-017-1987-z)

Additional file 2 for manuscript titled

"CSmetaPred: A consensus method for prediction of catalytic residues"

Preeti Choudhary[1], Shailesh Kumar[1,2], Anand Kumar Bachhawat[1] and Shashi Bhushan Pandit[*1]

[1]Department of Biological Sciences
Indian Institute of Science Education and Research, Mohali
Knowledge City, Sector 81, SAS Nagar,
Manuali PO 140306, India

[2]Current Address: Laboratory of Biochemistry and Genetics,
National Institute of Diabetes and Digestive and Kidney Diseases,
National Institutes of Health, Bethesda, MD 20892

*To whom correspondence should be addressed
Shashi Bhushan Pandit
Assistant Professor
Department of Biological Sciences
Indian Institute of Science Education and Research (IISER) – Mohali,
Knowledge City, Sector-81, SAS Nagar, Manauli PO 140306, India.
Ph: +91-172-22932382
Fax: +91-172-2240266, 2240124
Email: shashibp@iisermohali.ac.in

S1 Text: Extended Methods and Results sections

1. Compilation of EF-Fold, POOL-148 and PW-79 dataset

From earlier works, we took EF-Fold, POOL-160, and PW-79 datasets along with their respective catalytic residues definition. Enzymes with catalytic site present in more than one Protein Data Bank (pdb) chain are removed from these datasets. Obsolete pdb entry was either replaced with updated pdb entry or removed from the dataset. Subsequently, we renamed datasets as POOL-148, EF-Fold-164 and PW-79 depending on number of pdb entries in them, which are 148, 164 and 79 respectively. These 3 datasets are merged to construct a non-redundant (60% sequence identity) EF_POOL_PW dataset using CD-HIT. EF_POOL_PW dataset consists of 286 proteins.

2. Procedure to rank residues from CRpred, DISCERN and WCN

We used residue SVM score in CRpred to rank residues. The WCN and DISCERN residues are ranked based on WCN and DISCERN residue scores respectively.

3. Procedure to rank non-polar residues from EXIA2

EXIA2 server explicitly ranks amino acids having catalytic functional side chain based on *rank score* that includes polar/charged amino acids (R, N, D, C, Q, E, H, K, S, T, Y) and tryptophan. However, all residues should be ranked in order to facilitate EXIA2 comparison with meta-predictors and other methods. To rank rest non-functional side chain (non-polar) amino acids including glycine, we have followed second phase approach of EXIA2 as mentioned in Huang et al.([1]. From the EXIA2 server results page, for every residue in the ranked order, we fetch its neighbouring non-polar residues with WCN score > 0.9 and rank these neighbours based on WCN score. Importantly, neighbours follow the order list from EXIA2. For instance, neighbours of ranked 1 residues are ranked before rank 2 residue neighbours. Every non-polar residue is ranked only once, *i.e.* in its first occurrence as neighbour to a residue. This results in the ranked list of non-polar residues, which is referred to as NP-1. Next, we calculate WCN score for residues neither ranked in EXIA2 results page nor listed as neighbours in NP-1. These set of non-polar residues are ranked on WCN score that is referred to as NP-2. For final ranked list of residues, we first take EXIA2 ranked list of residues followed by NP-1 and NP-2.

4. Comparison of CSmetaPred predicted ranks of known catalytic residues with the best possible rank from all methods

We compared catalytic residue predicted ranks from CSmetaPred to their best possible ranks derived from scores of all of its constituent methods (EXIA2, DISCERN CRpred and CATSID). Here, the best possible rank is the minimum of ranks assigned to residues in five scores (zS_{rs}, zS_{wcn}, zS_{ca}, zS_{di} and zS_{cr}). Initially, we did not consider CATSID for the best rank analysis because it provides template matched to the query and since not all the residues could be matched to a template, we could derive residue scores (residue rank subsequently) only for

subset of residues that could lead to numerically low ranks. Apart from ranking only a subset of residues, an additional issue with ranking residues in CATSID hits is that more than one residue can have same score and identical ranks. In this analysis we have used CSAMAC dataset with 2912 catalytic residues. We restricted analysis for residues having the best rank ≤ 20. Importantly, most (94.8%) of catalytic residues have the best possible rank ≤ 20. Of these residues, ~26% and ~74% of catalytic residues showed no change/decrease (better performance) and increase (poor performance) in ranks with respect to the best rank respectively. For ~74% of residues with higher (poorer) ranks than the best possible rank cases, these do not have large increase in ranks as exhibited by mean and median increase in rank of 10.1 and 4 respectively. The detailed analysis of cases with large increase in CSmetaPred ranks showed that in most instances only one or two methods have a high residue scores, whereas other methods scores are relatively low, which lead to a decrease in the meta-score with subsequent increase in their ranked position.

5. Examples of large increase/decrease in CSmetaPred predicted ranks with respect to the best possible ranks from all methods

As discussed before, the best possible rank analysis provides the upper bound of meta-approach implemented in CSmetaPred. We analyzed CSmetaPred predicted ranks of some catalytic residues, which show large increase (poor predicted CSmetaPred ranks) or decrease (better ranks from CSmetaPred) in ranked positions with respect to the best possible rank. One of the catalytic residues (LYS-150) of enzyme phosphatidylinositol phosphate kinase (1b01B) is ranked at 28, 17, 25 and 17 by EXIA2, CRpred, WCN and DISCERN respectively (CATSID did not provide rank for this residue) that improves to rank 8 by CSmetaPred. Even though LYS-150 is not top residue in all methods, it is among top ranked residues and has consistent normalized residue scores of 1.1230, 2.4744, 1.4825 and 1.8339 from EXIA2, CRpred, WCN and DISCERN respectively. An example of increase in CSmetaPred predicted ranks is residue LYS-591 from enzyme isoleucyl-trna synthetase (1ileA) that is ranked at 120, 96, 569 and 6 by EXIA2, CRpred, WCN and DISCERN respectively. This residue (LYS-591) is ranked at 110 in CSmetaPred predicted ranks, mostly because normalized scores are not consistent and only one method (DISCERN) assigns relatively high scores as shown by normalized scores of 0.3521, 1.0424, -0.5579 and 2.647 from EXIA2, CRpred, WCN and DISCERN respectively. We have provided predicted ranks and scores of all benchmark proteins in our webserver.

6. Methodology used for homology modelling and construction of template library

The template library (LIB_TEMP) is constructed using PISCES server [2] with following criteria: resolution ≤2 Å, sequence length 40-1000 residues and non-redundant at 60% sequence identity.

The proteins from CSAMAC dataset having sequence identity from 40-90% and ≥ 70% coverage with templates from template library constitutes the dataset of proteins for homology modelling. To construct dataset for modelling we searched full-length protein sequences of each pdb entry from CSAMAC dataset against LIB_TEMP using *profile_build()* module of MODELLER to identity protein sequences, which have templates with sequence identity ranging from 40 to 90% and coverage ≥ 70% with respect to query sequence. This resulted in a set 335 protein used for homology modelling. We built models using MODELLER by taking only one template for a given query sequence. To identify template for 335 proteins, we parsed their *profile_build()* result and removed any template having sequence identity <40% and >90% or coverage <70% to query sequence. Further, based on sequence identity between query sequence (335 proteins) and templates we categorized query-template alignments into following sequence identity bins: 40–50%, 50–60%, 60–70%, 70–80% and 80-90%. Within each sequence identity category, we selected the best template, having maximum sequence identity, for a given query sequence to built model using MODELLER. Thus, resulted in 235, 135, 53, 22 and 23 models in 40–50%, 50–60%, 60–70%, 70–80% and 80-90% sequence identity category. For modelling, the alignment between query and template is constructed using *align2d()* module. The *automodel* class of MODELLER is used to build 10 different models. These models are ranked based on DOPE score. The model with lowest DOPE energy score is used as representative model in catalytic residue prediction. The present modelling strategy is a conservative structure prediction in template-based modelling category using MODELLER because multiple templates have been shown to improve quality of predicted structures.

7. Experimental study to investigate the role of predicted catalytic residue of E. coli γ-glutamylcysteine synthase enzyme

The top 20 predicted catalytic residues are shown in Table S8 (additional file 2). Among these H150, E328, E29, E27, D60 and E67 ranked at 2, 3, 5, 6, 8 and 12 respectively have been suggested to bind Mg^{2+} ion [3]. Hence, these were excluded from the mutational studies. From the remaining residues, we selected R330 (rank 1), R235 (rank 11), Y131 (rank 16) and R132 (rank 20) for mutational studies to investigate their role in catalysis.

8. Cloning, expression, and purification of mutant *gshA*

Different mutants of EcgshA were constructed by site overlap extension PCR using primer sets listed in Table S10 (Additional file 2). For protein expression, all mutant genes were cloned in pET23a expression vector at Sma1 and BamH1 sites. Protein was expressed and purified using the protocol as discussed earlier [4].

9. γ -GCS activity assay

The enzyme activity of mutant was assayed using protocol mentioned earlier [4]. Briefly, 1 µg purified protein was used to initiate the reaction in a standard reaction mixture of 800 µl contained 150 mM Tris/HCl, pH 8.0, 40 mM $MgCl_2$,

150 mM KCl, 2 mM sodium phosphoenol pyruvate, 20 mM sodium L-glutamate, 15 mM L-cysteine, 4 mM sodium ATP, 4 units of LDH (rabbit muscle type II) 1.6 units of PK (rabbit muscle), and 0.24 mM NADH. The change in NADH absorbance was monitored at 340 nm using a spectrophotometer (UV-1800, Shimadzu, UV spectrophotometer) for 5 minutes. The reaction was carried out at 25^0C. To determine the Km for cysteine, L-glutamate and ATP were used at 20 mM and 4 mM concentration respectively. The calculation of enzyme activity was done using GraphPad Prism software.

10.References

1. Chien YT, Huang SW: **Accurate Prediction of Protein Catalytic Residues by Side Chain Orientation and Residue Contact Density**. *PloS one* 2012, **7**.
2. Wang G, Dunbrack RL, Jr.: **PISCES: recent improvements to a PDB sequence culling server**. *Nucleic acids research* 2005, **33**(Web Server issue):W94-98.
3. Hibi T, Nii H, Nakatsu T, Kimura A, Kato H, Hiratake J, Oda J: **Crystal structure of gamma-glutamylcysteine synthetase: insights into the mechanism of catalysis by a key enzyme for glutathione homeostasis**. *Proceedings of the National Academy of Sciences of the United States of America* 2004, **101**(42):15052-15057.
4. Kumar S, Kasturia N, Sharma A, Datt M, Bachhawat AK: **Redox-dependent stability of the gamma-glutamylcysteine synthetase enzyme of Escherichia coli: a novel means of redox regulation**. *The Biochemical journal* 2013, **449**(3):783-794.

Table S2: **Table summarizing quantitative measures for ROC and PR curves.** Quantitative comparison of average ROC curves using AUCROC and MAS as single value measures for ROC and AUCPR and MAP are used to quantitatively compare average PR curves (see methods section). Median and average ranks of catalytic residues are also summarized.

Method	AUCROC	AUCPR	MAS	MAP	Median Rank	Average rank
**CSAMAC dataset (884 protein)**						
CSmetaPred_poc	0.967	0.347	0.968	0.514	6.0	12.0
CSmetaPred	0.960	0.324	0.961	0.489	7.0	14.4
EXIA2	0.908	0.167	0.910	0.317	14.5	33.0
CRpred	--	--	--	--	14.0	21.2
DISCERN	0.900	0.103	0.901	0.226	23.0	36.3
WCN	0.785	0.034	0.786	0.081	53.4	68.6
**EF_POOL_PW dataset (286 protein)**						
CSmetaPred_poc	0.974	0.366	0.975	0.531	5.5	9.9
CSmetaPred	0.970	0.338	0.972	0.502	6.3	11.1
EXIA2	0.926	0.172	0.927	0.333	12.8	25.9
CRpred	--	--	--	--	11.6	17.9
DISCERN	0.916	0.110	0.918	0.241	21.0	30.6
WCN	0.777	0.031	0.779	0.073	55.1	71.9
**POOL-148 dataset (148 protein)**						
CSmetaPred_poc	0.975	0.426	0.976	0.571	5.5	9.2
CSmetaPred	0.971	0.403	0.972	0.547	6.0	10.2
EXIA2	0.919	0.192	0.920	0.342	14.0	26.0
CRpred	--	--	--	--	13.2	17.9
DISCERN	0.911	0.117	0.913	0.241	22.5	30.7
WCN	0.793	0.034	0.795	0.078	52.9	64.4
**PW-79 dataset (79 protein)**						
CSmetaPred_poc	0.972	0.457	0.973	0.599	5.0	9.6
CSmetaPred	0.969	0.445	0.970	0.584	5.0	10.0
EXIA2	0.918	0.219	0.920	0.378	12.2	24.8
CRpred	--	--	--	--	12.5	16.7
DISCERN	0.914	0.129	0.916	0.261	20.5	27.5
WCN	0.772	0.035	0.775	0.084	46.5	62.2
**EF-Fold-164 dataset (164 protein)**						
CSmetaPred_poc	0.969	0.329	0.970	0.506	5.5	11.6
CSmetaPred	0.966	0.300	0.967	0.476	6.5	13.0
EXIA2	0.930	0.161	0.931	0.322	12.3	25.8
CRpred	--	--	--	--	11.0	17.2
DISCERN	0.913	0.104	0.914	0.237	18.0	32.6
WCN	0.759	0.028	0.761	0.069	59.9	80.5
**macie-254 dataset (254 protein)**						
CSmetaPred_poc	0.961	0.335	0.962	0.486	8.0	15.4
CSmetaPred	0.947	0.308	0.949	0.458	9.8	20.2
EXIA2	0.899	0.172	0.900	0.304	19.6	38.2
CRpred	--	--	--	--	19.3	26.6
DISCERN	0.886	0.097	0.887	0.201	28.3	44.3
WCN	0.791	0.039	0.793	0.082	57.7	68.7
**csalit-688 dataset (688 protein)**						

CSmetaPred_poc	0.971	0.366	0.972	0.534	5.3	10.4
CSmetaPred	0.966	0.343	0.967	0.509	6.0	12.0
EXIA2	0.911	0.169	0.913	0.325	12.7	31.0
CRpred	--	--	--	--	12.3	19.1
DISCERN	0.905	0.107	0.906	0.237	21.0	33.8
WCN	0.785	0.032	0.787	0.081	51.8	67.6
**UB-137 dataset (137 protein)**						
CSmetaPred_poc	0.974	0.468	0.976	0.620	5.0	7.9
CSmetaPred	0.970	0.433	0.971	0.582	5.6	9.0
EXIA2	0.908	0.220	0.910	0.378	14.7	25.0
CRpred	--	--	--	--	9.8	15.3
DISCERN	0.905	0.148	0.907	0.288	19.5	30.0
WCN	0.786	0.042	0.788	0.093	46.0	58.9

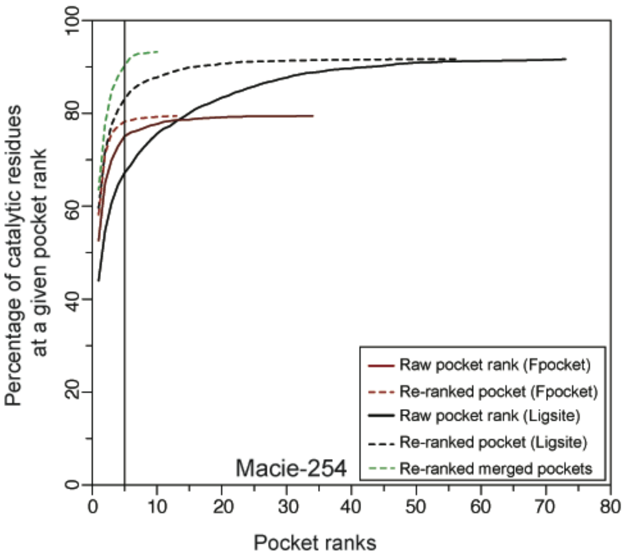

Figure S1: Cumulative distribution of catalytic residues present in predicted pockets. Plot showing cumulative distribution of catalytic residues within a given pocket rank on macie-254 dataset for: Pockets output from LIGSITE/Fpocket, re-ranked pockets using *poc_sc* score and merged top 5 re-ranked pockets. The vertical line shows that at pocket rank 5 both LIGSITE and Fpocket have achieved close to the maximum catalytic residues identified within predicted pockets. The drastic increase in catalytic residues fraction after re-ranking in LIGSITE could also be due to merging of pockets within LIGSITE.

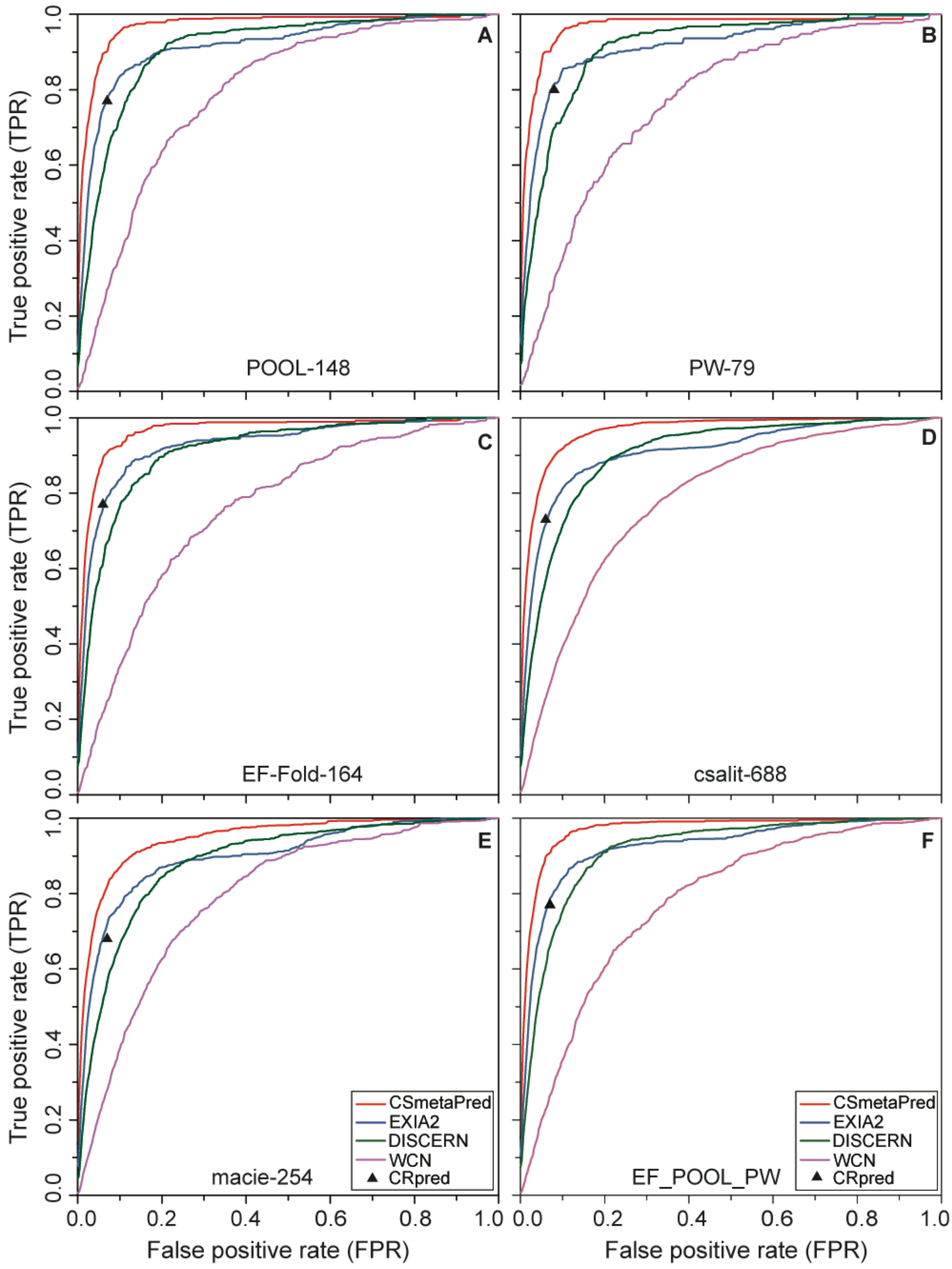

Figure S2: **Average ROC plots for all datasets.** Average ROC plots to show comparison among various predictors (EXIA2, DISCERN and WCN) on POOL-148, PW-79, EF-Fold-164, csalit-688, macie-254 and EF_POOL_PW datasets. CRpred SVM performance is shown as filled triangle.

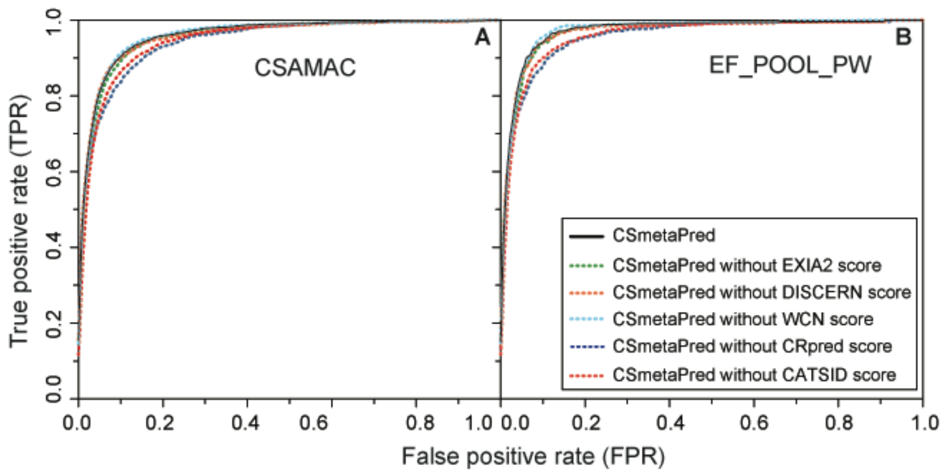

Figure S3: **Average ROC plots for modified CSmetaPred, wherein one score is excluded from meta-score computation.** Average ROC curves showing effect of individual method on the performance of CSmetaPred using CSAMAC (A) and EF_POOL_PW (B) datasets. All four methods contribute to different extent towards improving meta-score based ranking in CSmetaPred. It is apparent from ROC curves that excluding CRpred or CATSID residue score has maximum effect on prediction performance. This suggests that these two methods have major contribution in meta-score.

Table S3: **Summary of p-values obtained from Wilcoxon signed ranked statistical test.** Summary of p-values from Wilcoxon signed-rank test computed on AveS (MAS) and AP (MAP) measures to estimate statistical significance of performance difference between CSmetaPred and its constituent methods (EXIA2, DISCERN, and WCN).

Methods<br>Datasets	EXIA2	DISCERN	WCN
**CSAMAC dataset**			
MAS	<0.0001	<0.0001	<0.0001
MAP	<0.0001	<0.0001	<0.0001
**EF_POOL_PW dataset**			
MAS	<0.0001	<0.0001	<0.0001
MAP	<0.0001	<0.0001	<0.0001
**POOL-148 dataset**			
MAS	<0.0001	<0.0001	<0.0001
MAP	<0.0001	<0.0001	<0.0001
**PW-79 dataset**			
MAS	<0.0001	<0.0001	<0.0001
MAP	0.00002	<0.0001	<0.0001
**EF-Fold-164 dataset**			
MAS	<0.0001	<0.0001	<0.0001
MAP	<0.0001	<0.0001	<0.0001
**macie-254 dataset**			
MAS	<0.0001	<0.0001	<0.0001
MAP	<0.0001	<0.0001	<0.0001
**csalit-688 dataset**			
MAS	<0.0001	<0.0001	<0.0001
MAP	<0.0001	<0.0001	<0.0001
**UB-137 dataset**			
MAS	<0.0001	<0.0001	<0.0001
MAP	<0.0001	<0.0001	<0.0001

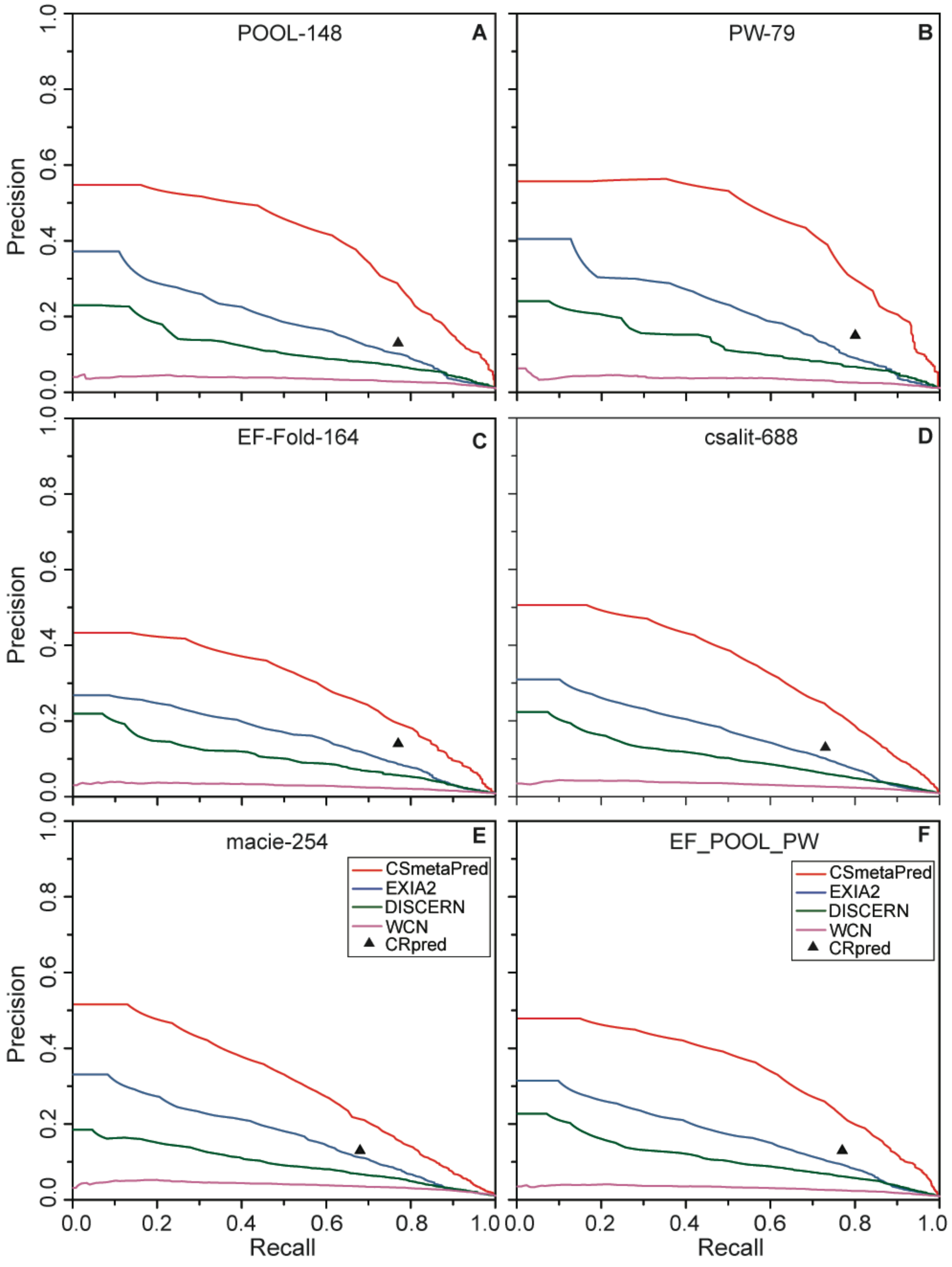

Figure S4: Average PR curves for benchmarking datasets. The figure showing average PR curves for CSmetaPred and other predictors (EXIA2, DISCERN and WCN) on EF_POOL_PW and five individual datasets. CRpred SVM performance is shown as filled triangle.

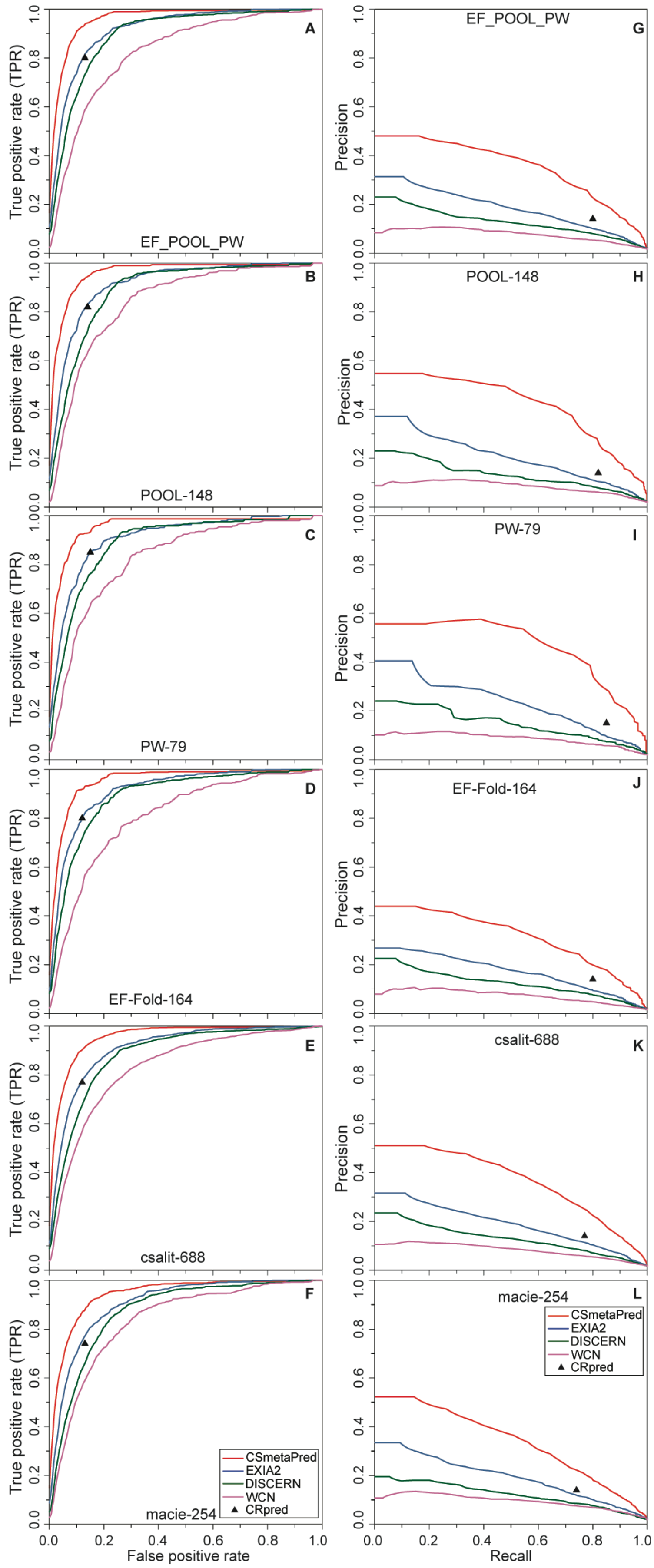

Figure S5: **Average ROC and PR curves for predictors considering ranked list of only polar/charged residues.** Average ROC (A-F) and average PR (G-L) curves on EF_POOL_PW and 5 datasets, when only polar/charged amino acids are ranked. CRpred SVM performance is shown as filled triangle.

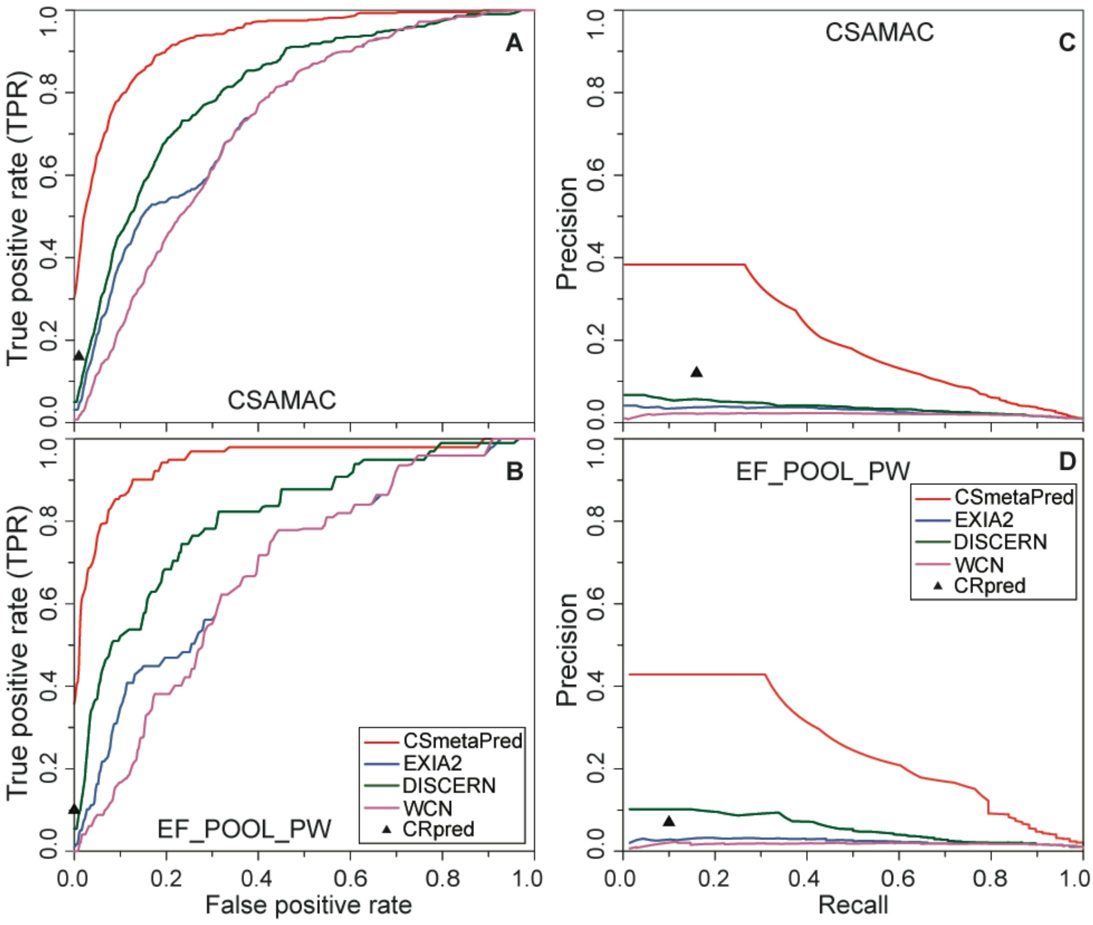

Figure S6: Average ROC and PR curves for predictors considering ranked list of only non-polar residues. Figure showing average ROC (A, B) and average PR (C, D) curves for various predictors (EXIA2, DISCERN and WCN) when only non-polar amino acids are ranked from CSAMAC and EF_POOL_PW datasets. CRpred SVM performance is shown as filled triangle.

Table S4: **Quantitative comparison of average PR and ROC curves for various methods when either polar/charged or non-polar residues are ranked separately.** Comparison of ROC/PR curves quantitative measures when only (A) polar/charged amino acids and (B) non-polar amino acids are ranked. Quantitative measure of ROC is AUCROC and MAS, whereas PR curves are compared using AUCPR and MAP. Median and average ranks of catalytic residues are also summarized.

A) Polar/charged amino acids

Method	AUCROC	AUCPR	MAS	MAP	Median rank	Average rank
**CSAMAC Polar dataset (873 protein)**						
CSmetaPred_poc	0.958	0.371	0.961	0.545	5.0	8.0
CSmetaPred	0.95	0.347	0.953	0.519	5.5	9.4
EXIA2	0.908	0.185	0.911	0.343	10.5	16.9
CRpred	--	--	--	--	11.0	15.7
DISCERN	0.879	0.126	0.883	0.265	15.0	22.7
WCN	0.829	0.084	0.832	0.186	20.3	28.1
**EF_POOL_PW Polar dataset (286 protein)**						
CSmetaPred_poc	0.964	0.382	0.967	0.555	4.9	7.3
CSmetaPred	0.959	0.354	0.962	0.528	5.1	8.3
EXIA2	0.916	0.184	0.919	0.349	9.8	16.4
CRpred	--	--	--	--	10.0	14.3
DISCERN	0.894	0.129	0.898	0.268	15.0	20.0
WCN	0.825	0.078	0.828	0.170	21.4	29.9
**POOL-148 Polar dataset (148 protein)**						
CSmetaPred_poc	0.966	0.446	0.968	0.601	4.5	6.8
CSmetaPred	0.961	0.420	0.963	0.577	4.9	7.6
EXIA2	0.914	0.207	0.917	0.364	9.8	15.6
CRpred	--	--	--	--	10.0	14.0
DISCERN	0.888	0.133	0.892	0.266	16.5	20.0
WCN	0.840	0.085	0.844	0.175	20.5	26.4
**PW-79 Polar dataset (79 protein)**						
CSmetaPred_poc	0.962	0.479	0.964	0.630	3.7	6.8
CSmetaPred	0.958	0.465	0.960	0.615	4.0	7.2
EXIA2	0.909	0.235	0.913	0.400	7.7	14.6
CRpred	--	--	--	--	10.0	13.2
DISCERN	0.889	0.148	0.893	0.292	12.5	17.5
WCN	0.824	0.087	0.828	0.185	18.5	25.0
**EF-Fold-164 Polar dataset (164 protein)**						
CSmetaPred_poc	0.960	0.342	0.962	0.526	5.0	8.3
CSmetaPred	0.955	0.315	0.957	0.498	5.5	9.5
EXIA2	0.915	0.172	0.919	0.338	9.6	16.6
CRpred	--	--	--	--	9.5	13.9
DISCERN	0.891	0.124	0.894	0.266	13.8	21.3
WCN	0.807	0.073	0.811	0.162	22.8	33.9
**macie-254 Polar dataset (251 protein)**						
CSmetaPred_poc	0.954	0.365	0.957	0.522	6.2	9.5
CSmetaPred	0.942	0.338	0.945	0.494	7.0	11.7
EXIA2	0.904	0.193	0.908	0.334	13.0	18.5
CRpred	--	--	--	--	13.8	18.8
DISCERN	0.872	0.122	0.876	0.240	18.5	25.9

WCN	0.834	0.095	0.837	0.188	22.5	28.3
**csalit-688 Polar dataset (679 protein)**						
CSmetaPred_poc	0.962	0.389	0.965	0.565	4.5	7.1
CSmetaPred	0.955	0.365	0.958	0.538	5.0	8.3
EXIA2	0.910	0.187	0.913	0.350	9.5	16.2
CRpred	--	--	--	--	10.0	14.6
DISCERN	0.883	0.129	0.886	0.275	14.0	21.5
WCN	0.830	0.082	0.834	0.190	19.5	27.5
**UB-137 Polar dataset (136 protein)**						
CSmetaPred_poc	0.968	0.498	0.971	0.650	4.0	5.6
CSmetaPred	0.962	0.462	0.966	0.611	4.5	6.2
EXIA2	0.906	0.241	0.910	0.406	9.0	13.4
CRpred	--	--	--	--	7.3	11.5
DISCERN	0.887	0.177	0.891	0.334	12.2	19.4
WCN	0.830	0.108	0.834	0.220	17.9	24.2

B) Non-polar amino acids

Method	AUCROC	AUCPR	MAS	MAP	Median rank	Average rank
**CSAMAC Non-polar dataset (193 protein)**						
CSmetaPred_poc	0.951	0.265	0.953	0.537	3.3	8.5
CSmetaPred	0.929	0.204	0.931	0.465	4.5	11.8
EXIA2	0.753	0.029	0.757	0.111	25.0	37.3
CRpred	--	--	--	--	13.0	18.9
DISCERN	0.808	0.037	0.812	0.150	21.0	30.7
WCN	0.725	0.020	0.729	0.068	32.3	42.0
**EF_POOL_PW Non-polar dataset (49 protein)**						
CSmetaPred_poc	0.953	0.301	0.955	0.588	2.0	6.3
CSmetaPred	0.945	0.260	0.948	0.537	3.0	7.7
EXIA2	0.706	0.024	0.710	0.090	30.0	38.1
CRpred	--	--	--	--	11.0	15.2
DISCERN	0.812	0.057	0.816	0.195	15.5	30.9
WCN	0.678	0.018	0.682	0.055	37.0	43.6
**POOL-148 Non-polar dataset (29 protein)**						
CSmetaPred_poc	0.974	0.353	0.976	0.641	1.5	4.6
CSmetaPred	0.971	0.328	0.973	0.611	2.0	4.8
EXIA2	0.725	0.029	0.729	0.111	26.0	34.9
CRpred	--	--	--	--	8.0	12.4
DISCERN	0.826	0.065	0.830	0.214	10.0	28.5
WCN	0.694	0.020	0.699	0.058	32.0	40.6
**PW-79 Non-polar dataset (18 protein)**						
CSmetaPred_poc	0.986	0.401	0.987	0.656	1.3	3.9
CSmetaPred	0.982	0.390	0.984	0.645	1.3	3.6
EXIA2	0.689	0.020	0.694	0.079	27.5	39.7
CRpred	--	--	--	--	10.5	13.6
DISCERN	0.868	0.060	0.873	0.189	8.5	29.7
WCN	0.673	0.016	0.678	0.061	28.5	43.1
**EF-Fold-164 Non-polar dataset (27 protein)**						
CSmetaPred_poc	0.938	0.291	0.941	0.572	3.0	7.4
CSmetaPred	0.929	0.235	0.932	0.503	3.0	9.6
EXIA2	0.676	0.020	0.680	0.085	37.5	45.0
CRpred	--	--	--	--	15.5	16.7

DISCERN	0.792	0.044	0.795	0.160	21.0	35.5
WCN	0.645	0.014	0.649	0.043	43.0	50.4
**macie-254 Non-polar dataset (68 protein)**						
CSmetaPred_poc	0.945	0.179	0.948	0.417	6.0	10.6
CSmetaPred	0.902	0.140	0.905	0.354	8.5	18.4
EXIA2	0.783	0.031	0.786	0.113	22.0	36.9
CRpred	--	--	--	--	15.8	24.3
DISCERN	0.771	0.031	0.775	0.118	28.0	39.0
WCN	0.752	0.022	0.756	0.066	33.3	40.6
**csalit-688 Non-polar dataset (133 protein)**						
CSmetaPred_poc	0.951	0.333	0.954	0.610	2.0	7.6
CSmetaPred	0.941	0.267	0.944	0.543	3.0	8.6
EXIA2	0.738	0.028	0.742	0.108	25.0	36.4
CRpred	--	--	--	--	9.0	15.5
DISCERN	0.824	0.042	0.828	0.173	16.0	26.7
WCN	0.709	0.019	0.714	0.063	31.0	42.0
**UB-137 Non-polar dataset (33 protein)**						
CSmetaPred_poc	0.938	0.214	0.941	0.452	4.3	7.1
CSmetaPred	0.922	0.162	0.926	0.366	5	8.9
EXIA2	0.716	0.028	0.721	0.088	26.0	31.0
CRpred	--	--	--	--	11.0	13.7
DISCERN	0.778	0.045	0.783	0.168	17.0	28.5
WCN	0.691	0.022	0.696	0.064	31.0	35.7

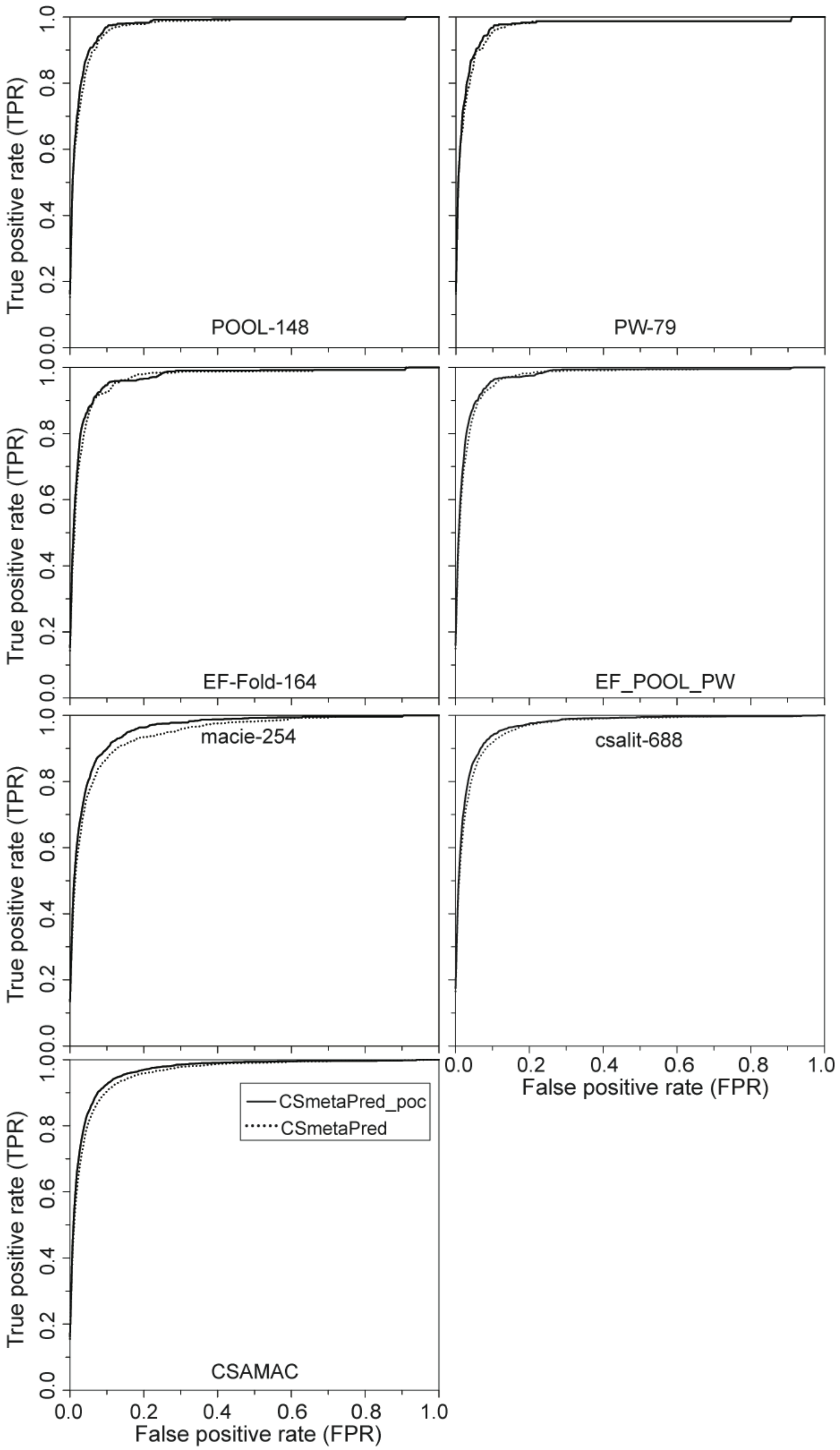

Figure S7: CSmetaPred_poc comparison with CSmetaPred using average ROC plots. Average ROC plots showing comparison of prediction performance between CSmetaPred and CSmetaPred_poc on all datasets.

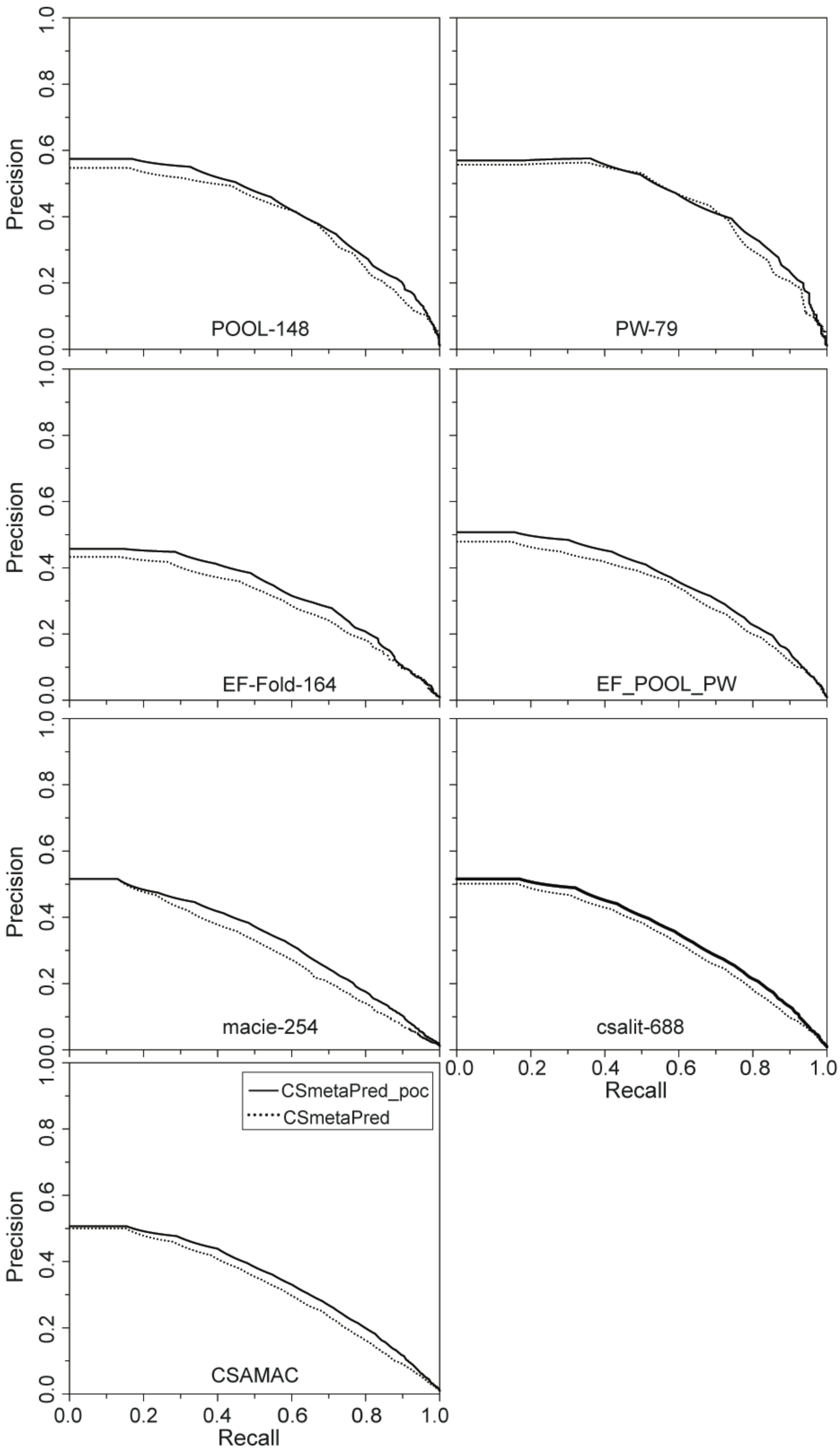

Figure S8: **CSmetaPred_poc comparison with CSmetaPred using average PR curves.** Average PR curves showing comparison of prediction performance between CSmetaPred_poc and CSmetaPred on all datasets.

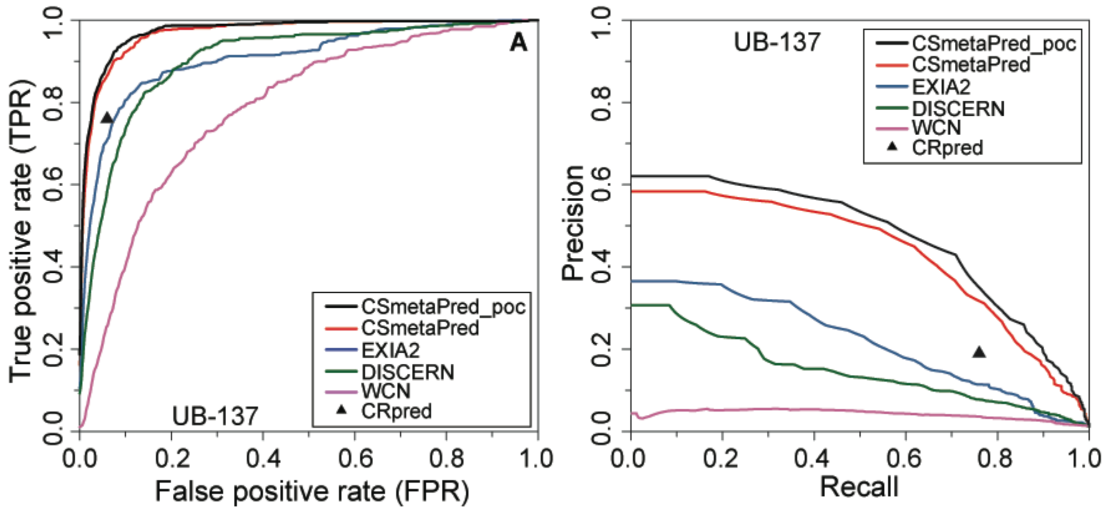

Figure S9: Average ROC and PR curves for various predictors on UB-137 datasets. Average ROC (A) and average PR (B) curves for meta-predictors and other predictors (EXIA2, DISCERN and WCN) on UB-137 dataset. CRpred SVM performance is shown as filled triangle.

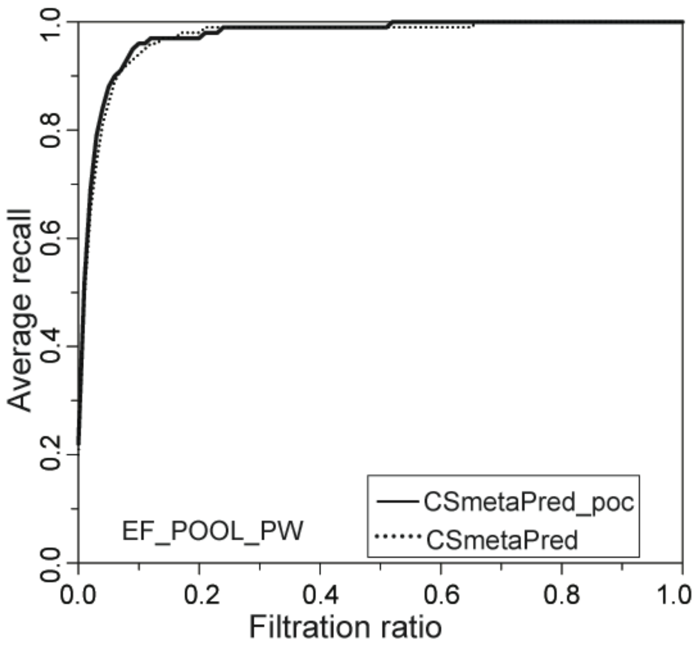

Figure S10: Filtration ratio plotted as a function of average recall. Average recall plotted as a function of filtration ratio for proteins in EF_POOL_PW dataset.

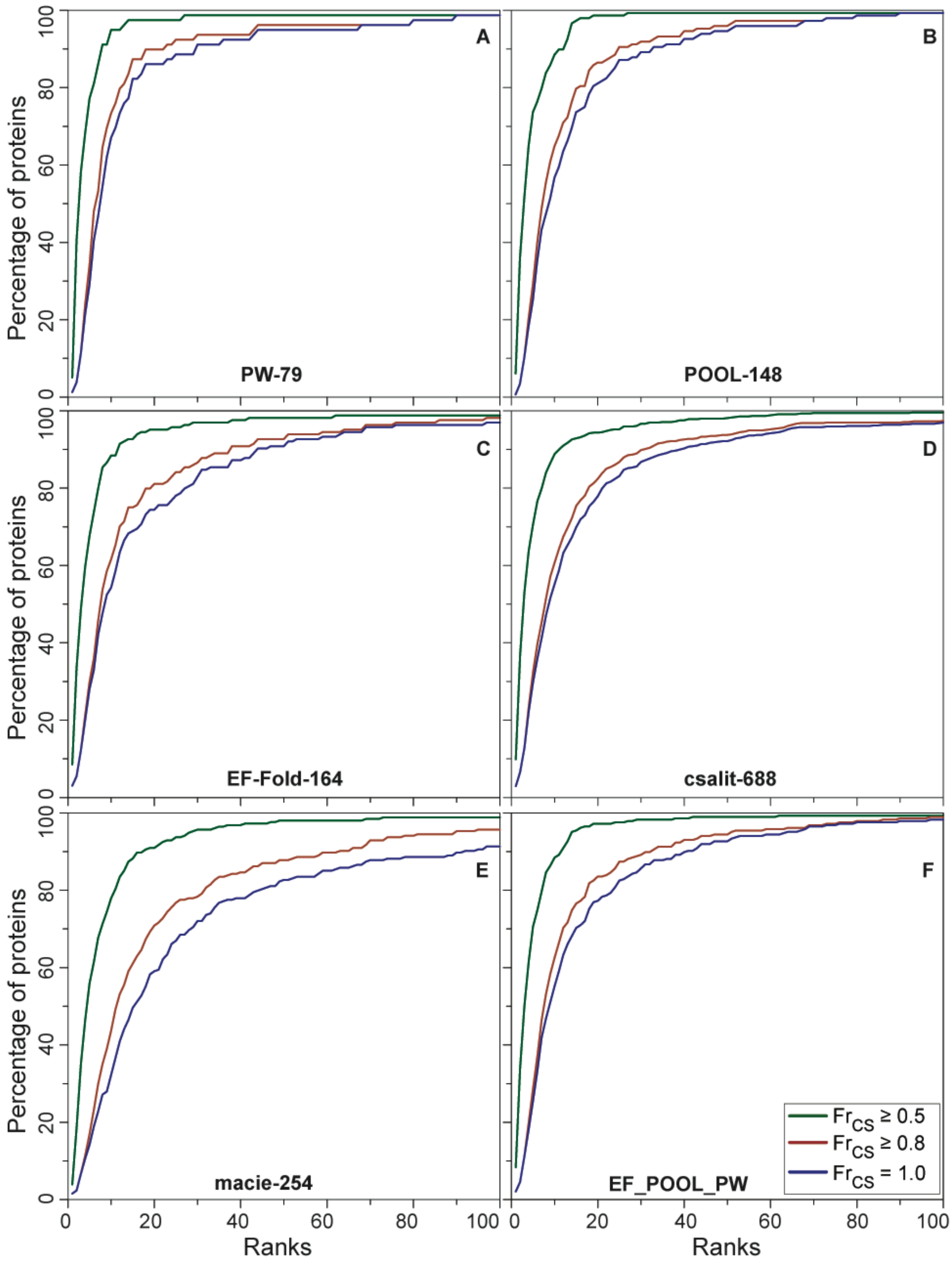

Figure S11: Fraction of proteins with catalytic residue coverage plotted as a function of ranks. Cumulative fraction of proteins (in percent) having at least 0.5, 0.8 and 1.0 catalytic residue coverage at various ranks ≤ 100.

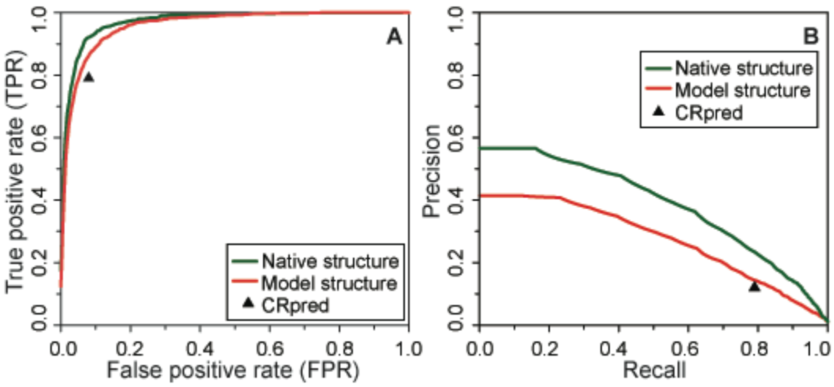

Figure S12: Comparison of CSmetaPred_poc prediction performance on models and their respective native structures. Figure showing prediction performances of CSmetaPred_poc on models and their respective native structures assessed using average ROC (A) and PR (B) curves. CRpred SVM performance is shown with filled triangle.

Table S5: **Comparison of CSmetaPred_poc prediction performance on modelled and their respective native structures.** Summary of quantitative analysis of ROC and PR curves using AUCROC/MAS and AUCPR/MAP respectively, for CSmetaPred_poc prediction on model and their native structures. Median and average ranks of catalytic residues are also summarized.

Method	AUCROC	AUCPR	MAS	MAP	Median rank	Average rank
**Modelled structures (468 structures)**						
CSmetaPred_poc	0.959	0.274	0.961	0.441	8.4	15.2
**Native structures (468 structures)**						
CSmetaPred_poc	0.971	0.386	0.973	0.542	6.3	10.6

Table S6: **Comparison of catalytic residues rank obtained from various methods on set of pdb entries mostly from benchmark dataset.** Summary of known catalytic residues ranks given by various predictors for pdb entries mostly from previous and present works.

PDB Chain	Catalytic residue	CSmetaPred_poc (Rank)	CSmetaPred (Rank)	EXIA2 (Rank)	CRpred (Rank)	DISCERN (Rank)
2y7fA	H46	2	2	1	6	10
	H48	4	6	2	12	19
	E143	6	12	11	4	11
	R226	1	1	3	2	1
	D231	5	7	4	9	4
	S82	14	23	6	19	75
	T106	17	30	17	41	69
	E230	3	3	5	3	5
1b73A	S8	3	5	9	12	12
	D7	2	3	8	3	13
	C178	1	2	2	6	11
	C70	4	1	1	4	1
1aukA	K302	4	4	8	15	16
	K123	3	3	16	10	1
	H229	2	2	2	8	6
	H125	1	1	1	11	3
	D281	7	8	34	1	40
	S150	13	26	23	36	276
	R73	8	10	12	5	5
1d6oA	D37	2	3	9	8	1
	I56	5	7	77	12	18
	Y82	4	6	11	5	15
1w27B	F400	1	1	464	8	27
1mpyA	H199	3	3	7	7	4
	H246	2	2	3	1	2
	Y255	5	6	5	24	28

1nspA	K16	6	6	5	8	19
	N119	5	5	7	12	1
2acyA	R23	2	3	4	3	1
	N41	8	10	2	9	16
1uokA	E255	2	2	4	2	37
	D199	3	3	3	2	80
	D329	4	4	2	19	36
1a7uA	H257	1	1	1	1	11
	S98	3	2	2	17	35
	D228	5	4	3	3	54
	M99	8	8	146	70	3
	F32	13	13	149	35	36
2plcA	H45	1	1	1	1	1
	H93	2	2	3	3	6
	D278	3	3	8	4	21
	D46	4	4	4	6	19
	R84	12	12	14	33	26
1nvmA	H21	5	6	14	28	1
	Y291	6	8	5	53	9
2admA	N105	1	1	1	1	7
	P160	4	5	189	21	2
	Y108	8	7	2	53	11
1jdwA	C407	1	1	2	2	3
	H303	2	2	1	3	1
	D254	3	3	11	9	8
	D305	7	7	8	7	5
1mekA	C36	4	4	5	2	8

3ecaA	12T	1	1	1	10	5
	T89	2	2	3	16	3
	K162	4	4	5	1	13
	D90	3	3	2	12	7
	Y25	17	30	36	25	108
1aa6A	R333	5	7	2	10	23
	H141	8	15	1	5	428
	K44	76	95	96	18	335
1dcoA	H62	1	1	1	4	1
	H63	4	4	2	3	4
	H80	3	3	3	1	3
	D89	2	2	5	2	5
	E81	20	45	20	7	50
	E58	42	31	16	25	13
1hrkA	H263	1	1	1	1	5
	E343	2	2	5	4	16
	E347	25	26	20	8	13
	D340	18	22	7	3	61
	R164	10	9	22	2	31
	Y165	6	5	8	11	4
3wywB	S11	3	4	4	12	21
	H52	28	18	20	14	4
	E66	8	13	3	3	13
1lxaA	H125	2	3	1	2	2
	D126	5	8	10	9	20
	G143	49	84	167	69	124

Table S7: Catalytic residue prediction for protein structures deposited in PDB after development of CSmetaPred. Meta-predictor prediction performance on pdb entries, with experimentally known catalytic residues, submitted in RCSB PDB database subsequent to development of meta-approach method. Catalytic residue ranks from CSmetaPred_poc is summarized in the table.

S. No.	PDB	UNIPROT	Catalytic residue (pdb residue number [sequence number])*	CSmetaPred_poc Rank
1	2n6jA	Q183R7	E185	1
			E143	2
			H146	3
			H142	4
			Y178	8
2	2nbqA	Q9UH17	H253	2
			E255	3
			C284	4
			C289	7
3	4ufoA	Q41415	H300	1
			D105	2
			L106 [W106]	3
			Y235	4
			Y154	9
			F33	10
4	2ruqA	Q13526	H13 [H59]	1
			H111 [H157]	2
			A67 [C113]	3
			K17 [K63]	8
			Q85 [Q131]	11
			T106 [T152]	17
			S69 [S115]	19
5	4zamA	P0AD64	S70	11
			K234	7
6	5b6aA	Q9HT57	D225	1
7	5b6sA	A8NI40	D109	2
			D224	5
			E276	9
8	5cqmX	P16442	C303[E303]	1
9	5dn5A	P15931	E184	5
			E223	81
10	5e2jA	Q9AJS0	D143	3
			E515	6
			D146	15
11	5e9eA	P42527	D766	3
12	5c17A	Q7DJN2	C165	1
			S105	2
			C102	3

13	4ywiA	Q07412	E165	4
14	5c0uA	P77072	C159<br>D99<br>C96	1<br>2<br>3
15	5ej3A	P26515	E177<br>E87	2<br>3
16	5g2gA	P07445	D40<br>Y16	1<br>5
17	5gmtA	E7FLQ2	Y142<br>Y190<br>K99	3<br>9<br>13
18	5j9qE	Q12692	Q338 [E338]	13
19	5jadA	Q13093	H351<br>S273<br>D296	6<br>8<br>13
20	5jmdA	Q89YS4	Y301<br>H431<br>N247	1<br>3<br>6
21	5ccdA	Q9ZMY2	E175<br>E13	1<br>6
22	4wyiA	O81770	H155	2
23	4x22A	Q8F5I5	H97 [H96]<br>E169 [E168]	1<br>4
24	4pixA	P21816	H155<br>Y157<br>S153	1<br>2<br>9
25	5idiA	B9K7M5	E164<br>G349 [E349]	2<br>10
26	5j7xA	B8N653	R337<br>D63	2<br>5
27	5j8cA	Q9H7Z6	S316 [C316]<br>Q350 [E350]	1<br>16
28	5lb1A	I6Y9J2	C354<br>H336	1<br>2
29	5kf6A	F7X6I3	C844	5
30	5h38A	F5HBQ9	C219	3
31	5haiA	P05364	G64 [S64]	18
32	5grrA	A0A0R6L508	T285	21
33	5ezqA	P27282	C477	21
34	5jciA	Q652L6	R320<br>Y349	22<br>49
35	5d0nA	Q195N6	G298	42
36	4z85A	A4UVY1	Y193<br>C194	74<br>137

*If the residue name or number from 'ATOM' record of PDB id does not match with Uniprot sequence residue name/number is also mentioned.

Table S8: **Predicted catalytic residues of γ -glutamylcysteine synthase.** List of top 20 predicted catalytic residues of γ-glutamylcysteine synthase from *E. coli* (pdbid: 1v4gA) by CSmetaPred_poc.

**Rank**	**Residue name**	**Residue number**
1	ARG	330
2	HIS	150
3	GLU	328
4	ARG	304
5	GLU	29
6	GLU	27
7	LYS	306
8	ASP	60
9	GLU	296
10	ASP	333
11	ARG	235
12	GLU	67
13	ARG	32
14	ASN	297
15	LYS	128
16	TYR	131
17	HIS	44
18	ASN	152
19	TRP	100
20	ARG	132

Figure S13: *In vivo* complementation assay of GshA wild type and mutant enzymes. Figure showing in vivo complementation assay of predicted catalytic residues mutants of GshA enzyme. *Saccharomyces cerevisiae* strain ABC1195 plasmids bearing WT gshA or the different cysteine binding residues gshA mutant gene cloned under TEF promoter. The transformants were grown overnight in SD+GSH medium and used to re-inoculate secondary culture. Cells were harvested at OD_{600} = 0.6 and serially diluted (0.2 to 0.0002 OD_{600}). 10 μl was spotted on SD medium with or without GSH as sole source of organic sulphur. The vector pTEF416 and EcGshA were used as negative and positive control respectively.

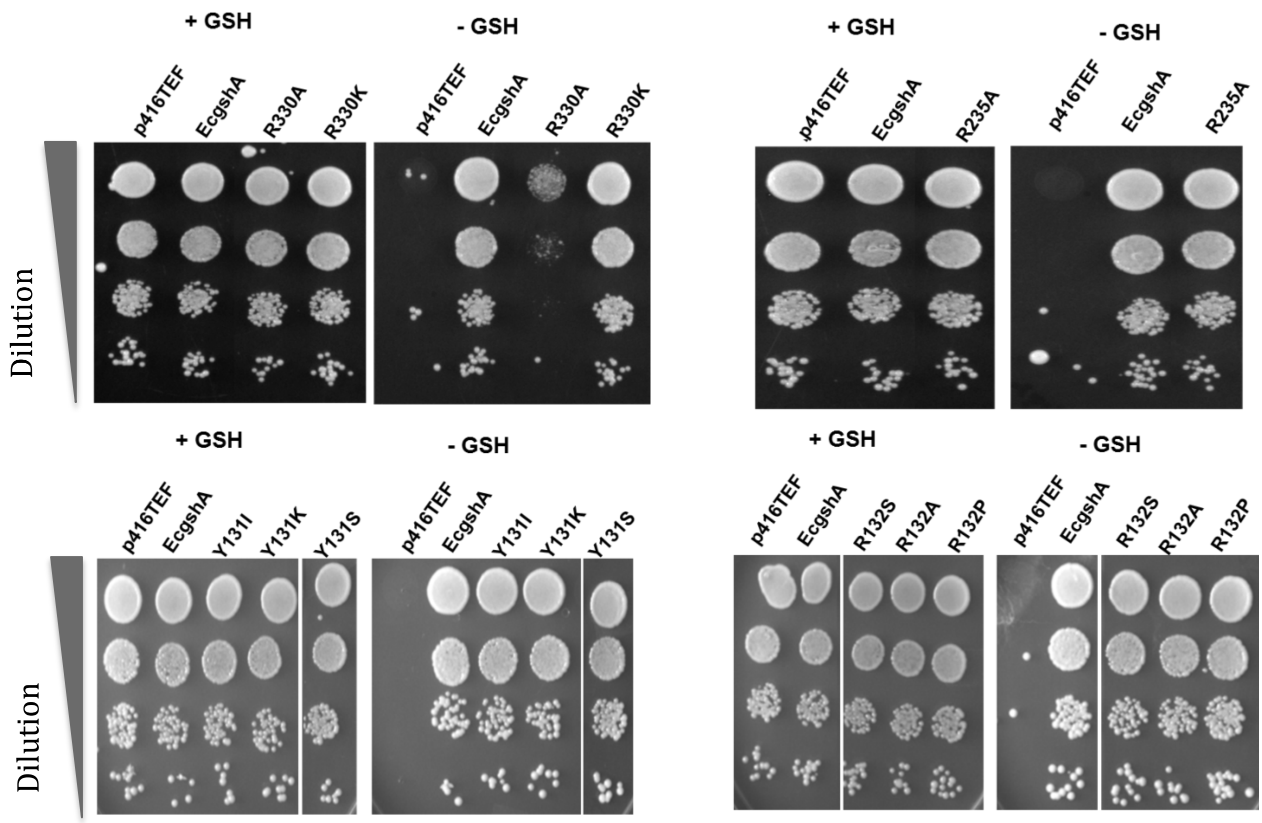

Table S9: **Summary of relative enzymatic activity of GshA mutants.** Table showing enzyme activity (*in vitro*) of GshA mutants calculated with respect to wild type activity of enzyme.

**Mutant**	**Relative enzyme activity (in % with respect to wild type)**
**Wild type**	100
**R330**	
R330K	Not determined
R330A	0
**Y131**	
Y131S	1.3
**R132**	
R132A	0.4

Table S10: **Primers used for cloning of GshA gene from *E. coli*.** List of primers sets used in site overlap extension PCR in cloning mutant GshA.

**Primer name**	**Primer Sequence (5' to 3')**
R235A FW	CGTATGCGACCTCTCTTGCTTTGAGCGATCTCGGCTATACC
R235A Rev	GCCGAGATCGCTCAAAGCAAGAGAGGTCGCATACGGCAGG
R330A Fw	GAATATATTGAAGTGGCTTCGCTGGACATCAACCCGTTCTCGC
R330A Rev	CGGGTTGATGTCCAGCGAAGCCACTTCAATATATTCAATGCCG
R330K Fw	GAATATATTGAAGTGAAGTCGCTGGACATCAACCCGTTCTCGC
R330K Rev	GGGTTGATGTCCAGCGACTTCACTTCAATATATTCAATGCCGC
Ec Y131NNK Rev	GATTTTTCAGCCCTTCACGMNNCAGCGTTTTAAAGCGTCCGGTG
Ec Y131NNK Fw	GCTTTAAAACGCTGNNKCGTGAAGGGCTGAAAAATCGCTACGG
Ec Y131NNK Rev	GATTTTTCAGCCCTTCACGMNNCAGCGTTTTAAAGCGTCCGGTG
Ec Y131NNK Fw	GCTTTAAAACGCTGNNKCGTGAAGGGCTGAAAAATCGCTACGG
Ec R132NNK Fw	GCTTTAAAACGCTGTATNNKGAAGGGCTGAAAAATCGCTACGG
Ec R132NNK Rev	GATTTTTCAGCCCTTCMNNATACAGCGTTTTAAAGCGTCCGG
EcGshACHis BamH1 Rev	ACCTAGGGATCCTTAATGATGGTGATGGTGATGGGCGTGTTTTTCCAGCCACACC
EcGshASma1 Fw	GATATCCCCGGGATGATCCCGGACGTATCACAGG

Fw: Forward and Rev: Reverse

Figure S14: Residues rank comparison between EXIA2 server and in-house recoded EXIA2. Plot showing residues rank comparison from EXIA2 server output and in-house recoded EXIA2 for (A) all residues, and (B) catalytic residues. The Pearson correlation coefficient between ranks for all residues obtained from EXIA2 and in-house program is 0.86, and the same for catalytic residues is 0.55.

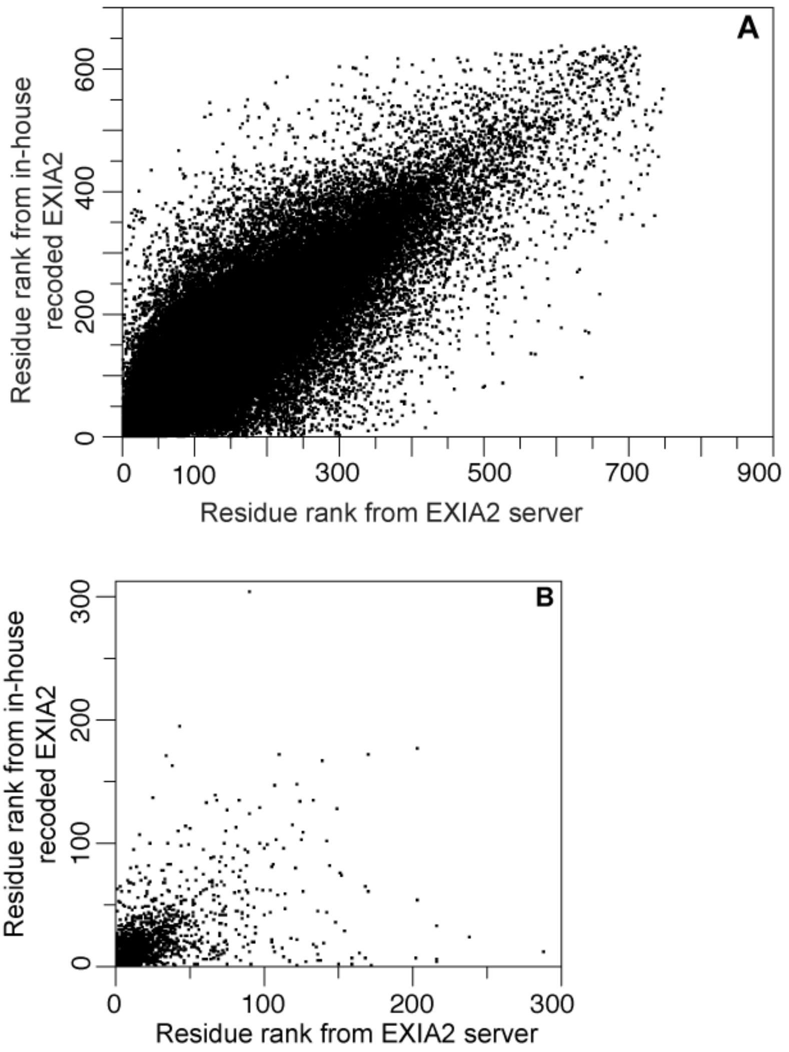

Supplement: Supplementary file 2 — Extended Methods and Results sections. Table S2. Table summarizing quantitative measures for ROC and PR curves. Quantitative comparison of average ROC curves using AUCROC and MAS as single value measures of ROC and AUCPR and MAP are used to quantitatively compare average PR curves (see Methods section). Median and average ranks of catalytic residues are also summarized. Table S3. Summary of p-values obtained from Wilcoxon signed ranked statistical test. Summary of p-values from Wilcoxon signed-rank test computed on AveS (MAS) and AP (MAP) measures to estimate statistical significance of performance difference between CSmetaPred and its constituent methods (EXIA2, DISCERN, and WCN). Table S4. Quantitative comparison of average PR and ROC curves for various methods when either polar/charged or non-polar residues are ranked separately. Comparison of ROC/PR curves quantitative measures when only (A) polar/charged amino acids and (B) non-polar amino acids are ranked. Quantitative measure of ROC is AUCROC and MAS, whereas PR curves are compared using AUCPR and MAP. Median and average ranks of catalytic residues are also summarized. Table S5. Comparison of CSmetaPred_poc prediction performance on modelled and their respective native structures. Summary of quantitative analysis of ROC and PR curves using AUCROC/MAS and AUCPR/MAP respectively, for CSmetaPred_poc prediction on model and their respective native structures. Median and average ranks of catalytic residues are also summarized. Table S6. Comparison of catalytic residues rank obtained from various methods on set of pdb entries mostly from benchmark dataset. Summary of known catalytic residues ranks given by various predictors for pdb entries mostly from previous and present works. Table S7. Catalytic residue prediction for protein structures deposited in PDB after development of CSmetaPred. Meta-predictor prediction performance on pdb entries, with experimentally known catalytic residues, submitted in RCSB PDB datab [file 12859_2017_1987_MOESM2_ESM.pdf]
